# scAI: an unsupervised approach for the integrative analysis of parallel single-cell transcriptomic and epigenomic profiles

**DOI:** 10.1186/s13059-020-1932-8

**Published:** 2020-02-03

**Authors:** Suoqin Jin, Lihua Zhang, Qing Nie

**Affiliations:** 1 Department of Mathematics, University of California, Irvine, CA 92697 USA; 2 The NSF-Simons Center for Multiscale Cell Fate Research, University of California, Irvine, CA 92697 USA; 3 Department of Developmental and Cell Biology, University of California, Irvine, CA 92697 USA

**Keywords:** Integrative analysis, Single-cell multiomics, Simultaneous measurements, Sparse epigenomic profile

## Abstract

Simultaneous measurements of transcriptomic and epigenomic profiles in the same individual cells provide an unprecedented opportunity to understand cell fates. However, effective approaches for the integrative analysis of such data are lacking. Here, we present a single-cell aggregation and integration (scAI) method to deconvolute cellular heterogeneity from parallel transcriptomic and epigenomic profiles. Through iterative learning, scAI aggregates sparse epigenomic signals in similar cells learned in an unsupervised manner, allowing coherent fusion with transcriptomic measurements. Simulation studies and applications to three real datasets demonstrate its capability of dissecting cellular heterogeneity within both transcriptomic and epigenomic layers and understanding transcriptional regulatory mechanisms.

## Background

The rapid development of single-cell technologies allows for dissecting cellular heterogeneity more comprehensively at an unprecedented resolution. Many protocols have been developed to quantify transcriptome [1], such as CEL-seq2, Smart-seq2, Drop-seq, and 10X Chromium, and techniques that measure single-cell chromatin accessibility (scATAC-seq) and DNA methylation have also become available [2]. More recently, several single-cell multiomics technologies have emerged for measuring multiple types of molecules in the same individual cell, such as scM&T-seq [3], scNMT-seq [4], scTrio-seq [5], sci-CAR-seq [6], and scCAT-seq [7]. The resulting single-cell multiomics data has potential of providing new insights regarding the multiple regulatory layers that control cellular heterogeneity [8, 9].

Gene expression is often regulated by transcription factors (TFs) via interaction with cis-regulatory genomic DNA sequences located in or around target genes [10, 11]. Epigenetic modifications, including changes in chromatin accessibility and DNA methylation, play crucial roles in the regulation of gene expression [12, 13]. Many tools have been developed for the integrative analysis of transcriptomic and epigenomic profiles in bulk samples [14–16]. For example, Zhang et al. integrated the analysis of bulk gene expression, DNA methylation, and microRNA expression using joint nonnegative matrix factorization [16]. Argelaguet et al. [17] presented MOFA, a generalization of principal component analysis (PCA) which is applicable to both bulk and single-cell datasets [18, 19].

Single-cell multiomics data are inherently heterogenous and highly sparse [9]. Although many integration methods initially developed for bulk data might be applicable to such data, it has become increasingly clear that new and different computational strategies are required due to unique characteristics of single-cell data [9]. In particular, scATAC-seq data are extremely sparse (e.g., over 99% zeros in sci-CAR-seq) and nearly binary [20], thus making it difficult to reliably identify accessible (or methylated) regions in a cell.

A growing number of methods have been developed for scRNA-seq data integration [21–23]. However, only few methods have been proposed for integrating multiomics profiles, and these methods were designed for data measured in different cells (i.e., not the same single cells) but sampled from the same cell population [22–25]. MATCHER used a Gaussian process latent variable model to compute the “pseudotime” for every cell in each omics layer and to predict the correlations between transcriptomic and epigenomic measurements from different cells of the same type [24]. A coupled nonnegative matrix factorization method performed clustering of single cells sequenced by scRNA-seq and scATAC-seq through constructing a “coupling matrix” for regulatory elements and gene associations [25]. Recently, Seurat (version 3) [22] and LIGER [23] were developed for integrating scRNA-seq and single-cell epigenomic data. Both of these methods first transform the epigenomic data into a synthetic scRNA-seq data through estimating a “gene activity matrix,” and then identify “anchors” between this synthetic data and scRNA-seq data through aligning them in a low-dimensional space. The gene activity matrix is created by simply summing all counts within the gene body +2 kb upstream. Such strategy may introduce improper synthetic data due to complex transcriptional regulatory mechanisms between gene expression and chromatin accessibility. The improper synthetic data may further lead to imperfect alignment when they are applied to parallel transcriptomic and epigenomic profiles, and likely affect downstream analysis. Moreover, the inference of interactions between transcriptomics and epigenetics often requires both measurements from the same single cell [8].

Here, we present a single-cell aggregation and integration (scAI) approach to integrate transcriptomic and epigenomic profiles (i.e., chromatin accessibility or DNA methylation) that are derived from the same cells. Unlike existing integration methods [16, 17, 22, 24–26], scAI takes into consideration the extremely sparse and near-binary nature of single-cell epigenomic data. Through iterative learning in an unsupervised manner, scAI aggregates epigenomic data in subgroups of cells that exhibit similar gene expression and epigenomic profiles. Those similar cells are computed through learning a cell-cell similarity matrix simultaneously from both transcriptomic and aggregated epigenomic data using a unified matrix factorization model. As such, scAI represents the transcriptomic and epigenomic profiles with biologically meaningful low-rank matrices, allowing identification of cell subpopulations; simultaneous visualization of cells, genes, and loci in a shared two-dimensional space; and inference of the transcriptional regulatory relationships. Through applications to eight simulated datasets and three published datasets, and comparisons with recent multiomics data integration methods, scAI is found to be an efficient approach to reveal cellular heterogeneity by dissecting multiple regulatory layers of single-cell data.

## Results

### Overview of scAI

To deconvolute heterogeneous single cells from both transcriptomic and epigenomic profiles, we aggregate the sparse/binary epigenomic profile in an unsupervised manner to allow coherent fusion with transcriptomic profile while projecting cells into the same representation space using both the transcriptomic and epigenomic data. Using the normalized scRNA-seq data matrix *X*_1_ (*p* genes in *n* cells) and the single-cell chromatin accessibility or DNA methylation data matrix *X*_2_ (*q* loci in *n* cells) as an example, we infer the low-dimensional representations via the following matrix factorization model:

1 $$ {\min}_{W_1,{W}_2,H,Z\ge 0}\alpha {\left\Vert {X}_1-{W}_1H\right\Vert}_F^2+{\left\Vert {X}_2\left(Z\circ R\right)-{W}_2H\right\Vert}_F^2+\lambda {\left\Vert Z-{H}^TH\right\Vert}_F^2+\gamma \sum \limits_j{\left\Vert {H}_{.j}\right\Vert}_1^2, $$

where *W*_1_ and *W*_2_ are the gene loading and locus loading matrices with sizes *p* × *K* and *q* × *K* (*K* is the rank), respectively. Each of the *K* columns is considered as a factor, which often corresponds to a known biological process/signal relating to a particular cell type. ${W}_1^{ik}$ and ${W}_2^{ik}$ are the loading values of gene *i* and locus *i* in factor *k*, and the loading values represent the contributions of gene *i* and locus *i* in factor *k*. *H* is the cell loading matrix with size *K* × *n* (*H*_*.j*_ is the *j*th column of *H*), and the entry *H*^*kj*^ is the loading value of cell *j* when mapped onto factor *k*. *Z* is the cell-cell similarity matrix. *R* is a binary matrix generated by a binomial distribution with a probability *s*. *α*, *λ*, *γ* are regularization parameters, and the symbol ∘ represents dot multiplication. The model aims to address two major challenges simultaneously: (i) the extremely sparse and near-binary nature of single-cell epigenomic data and (ii) the integration of this binary epigenomic data with the scRNA-seq data, which are often continuous after being normalized.

#### Aggregation of epigenomic profiles through iterative refinement in an unsupervised manner

To address the extremely sparse and binary nature of the epigenomic data, we aggregate epigenomic data of similar cells based on the cell-cell similarity matrix *Z*, which is simultaneously learned from both transcriptomic and epigenomic data iteratively. Epigenomic data can be simply aggregated by *X*_2_*Z*. However, this strategy may lead to over-aggregation, for example, in one subpopulation, similar cells exhibit almost the same aggregated epigenomic signals, which improperly reduces the cellular heterogeneity. To reduce such over-aggregation, a binary matrix *R*, generated from a binomial distribution with probability *s*, is utilized for randomly sampling of similar cells. After normalizing *H* with the sum of each row equaling 1 in each iteration step and *Z°R* with the sum of each column equaling 1, then the aggregated epigenomic profiles are represented by *X*_2_(*Z* ∘ *R*). The *i*th column of *X*_2_(*Z* ∘ *R*) represents the weighted combination of epigenomic signals from some cells similar to the *i*th cell. These strategies not only enhance epigenomic signals, but also maintain cellular heterogeneity within *and* between different subpopulations.

#### Integration of binary and count-valued data via projection onto the same low-dimensional space

**Fig. 1:**
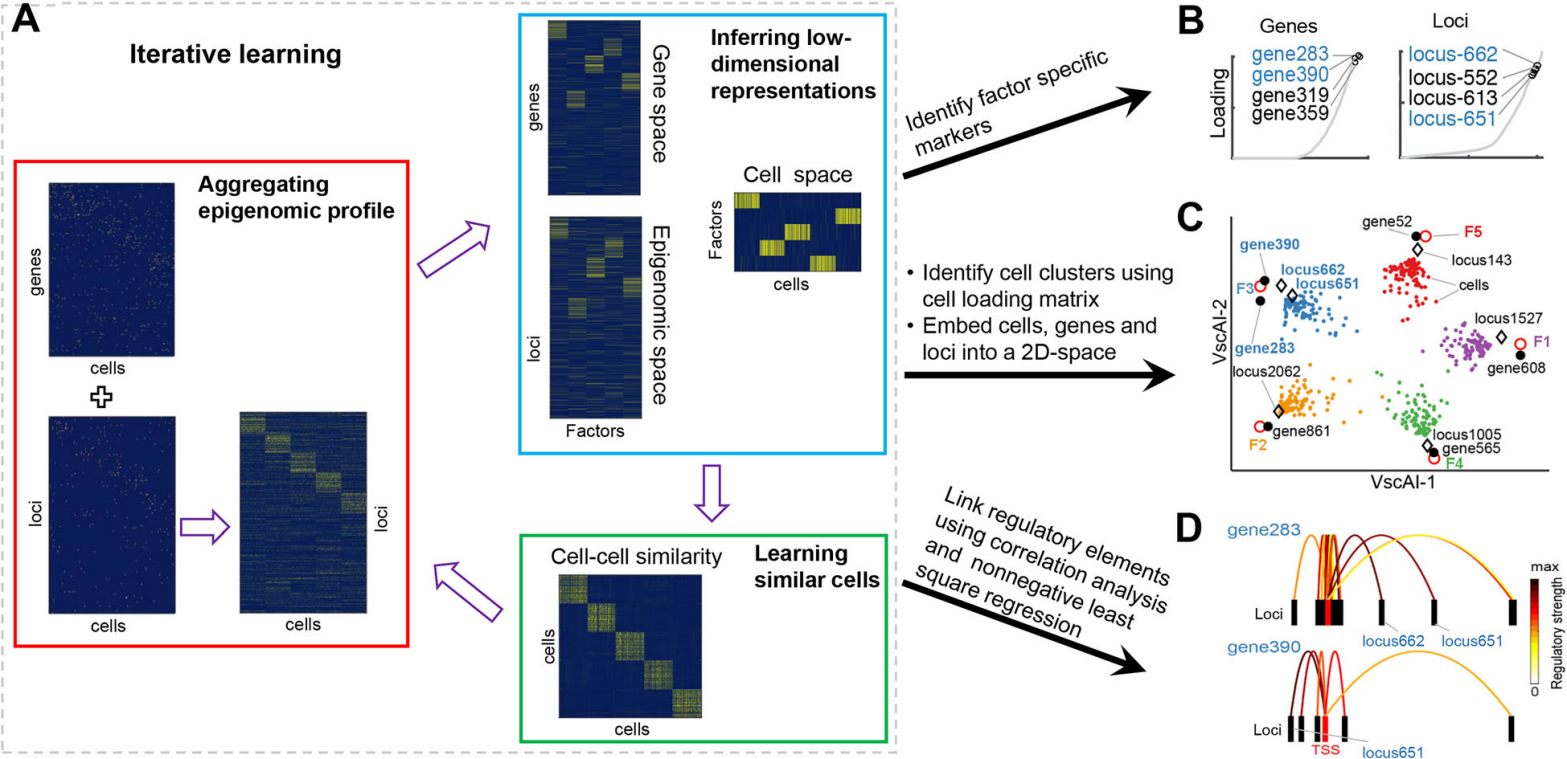
Overview of scAI. **a** scAI learns aggregated epigenomic profiles and low-dimensional representations from both transcriptomic and epigenomic data in an iterative manner. scAI uses parallel scRNA-seq and scATAC-seq/single cell DNA methylation data as inputs. Each row represents one gene or one locus, and each column represents one cell. In the first step, the epigenomic profile is aggregated based on a cell-cell similarity matrix that is randomly initiated. In the second step, transcriptomic and aggregated epigenomic data are simultaneously decomposed into a set of low-rank matrices. Entries in each factor (column) of the gene loading matrix (gene space), locus loading matrix (epigenomic space), and cell loading matrix (cell space) represent the contributions of genes, loci, and cells for the factor, respectively. In the third step, a cell-cell similarity matrix is computed based on the cell loading matrix. These three steps are repeated iteratively until the stop criterion is satisfied. **b** scAI ranks genes and loci in each factor based on their loadings. For example, four genes and loci are labeled with the highest loadings in factor 3. **c** Simultaneous visualization of cells, marker genes, marker loci, and factors in a 2D space by an integrative visualization method VscAI, which is constructed based on the four low-rank matrices learned by scAI. Small filled dots represent the individual cells, colored by true labels. Large red circles, black filled dots, and diamonds represent projected factors, marker genes, and marker loci, respectively. **d** The regulatory relationships are inferred via correlation analysis and nonnegative least square regression modeling of the identified marker genes and loci. An arch represents a regulatory link between one locus and the transcription start site (TSS) of each marker gene. The arch colors indicate the Pearson correlation coefficients for gene expression and loci accessibility. The red stem represents the TSS region of the gene, and the black stem represents each locus

Through aggregation, the extremely sparse and near-binary data matrix *X*_2_ is transformed into the signal-enhanced continuous matrix *X*_2_(*Z* ∘ *R*), allowing coherent fusion with transcriptomic measurements (Fig. 1a). These two matrices are projected onto a common coordinate system represented by the first two terms in the optimization model (Eq. (1)). In this way, cells are mapped onto a *K*-dimensional space with the cell loading matrix *H*, and the cell-cell similarity matrix *Z* is approximated by *H′H*, as represented by the third term in Eq. (1). The sparseness constraint on each column of *H* is added by the last term of Eq. (1).

#### Downstream analysis using the inferred low-dimensional representations

scAI simultaneously decomposes transcriptomic and epigenomic data into multiple biologically relevant factors, which are useful for a variety of downstream analyses (Fig. 1b–d). (1) The cell subpopulations can be identified from the cell loading matrix *H* using a Leiden community detection method (see the “Methods” section). (2) The genes and loci in the *i*th factor are ranked based on the loading values in the *i*th columns of *W*_1_ and *W*_2_ (see Fig. 1b and the “Methods” section). (3) To simultaneously analyze both gene and loci information associated with cell states, we introduce an integrative visualization method, VscAI. By combining these learned low-rank matrices (*W*_1_, *W*_2_, *H*, and *Z*) with the Sammon mapping [27] (see the “Methods” section), VscAI simultaneously projects genes and loci that separate the cell states into a two-dimensional space alongside the cells (Fig. 1c). (4) Finally, the regulatory relationships between the marker genes and the chromosome regions in each factor or cell subpopulation are inferred by combining the correlation analysis and the nonnegative least square regression modeling of gene expression and chromatin accessibility (see Fig. 1d and the “Methods” section). Overall, these functionalities allow the deconvolution of cellular heterogeneity and reveal regulatory links from transcriptomic and epigenomic layers.

### Model validation and comparison using simulated data

To evaluate scAI, we simulated eight single-cell datasets with the sparse count data matrix *X*_1_ and the sparse binary data matrix *X*_2_ (i.e., paired scRNA-seq and scATAC-seq). To recapitulate the properties of the single-cell multiomics data (e.g., a high abundance of zeros and binary epigenetic data), we generated bulk RNA-seq and DNase-seq profiles from the same sample with MOSim [28]. Then, we added the effects of dropout and binarized the data. A detailed description of the simulation approach and the simulated data are shown in Additional file 1: Supplementary methods (*Simulation datasets*) and Additional file 2: Table S1. These datasets encompass eight scenarios with different transcriptomic/epigenomic properties: different sparsity levels (dataset 1), different noise levels (dataset 2), missing clusters in the epigenomic profiles (i.e., clusters defined from gene expression do not reflect epigenetic distinctions) (dataset 3), missing clusters in the transcriptomic profiles (i.e., clusters defined from epigenetic profile do not reflect gene expression distinctions) (dataset 4), discrete cell states (dataset 5), a continuous biological process (dataset 6), imbalanced cluster sizes with the same number of clusters defined from both transcriptomic and epigenomic profiles (dataset 7), and imbalanced cluster sizes with missing clusters in the epigenomic profiles (dataset 8).

**Fig. 2:**
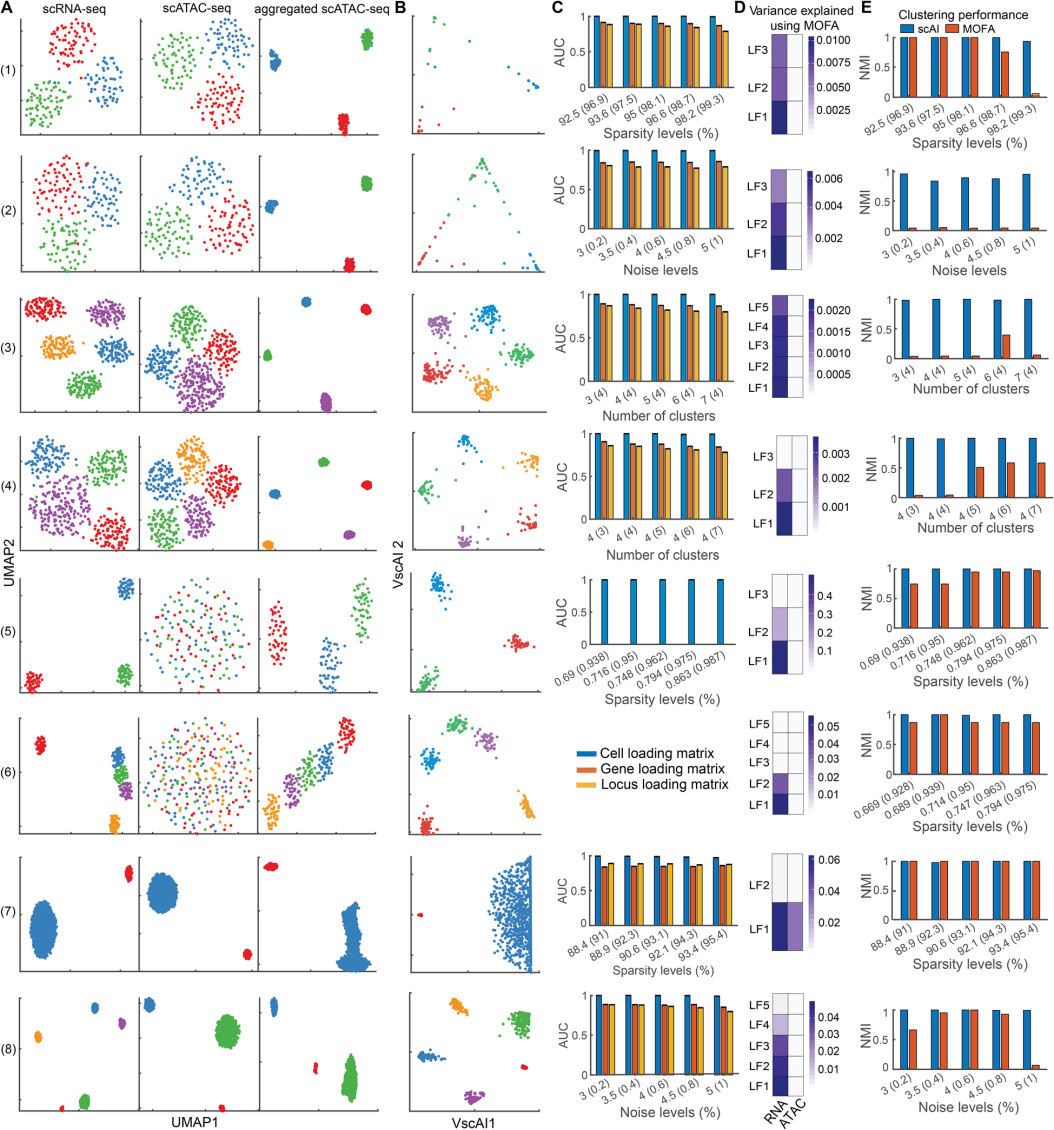
Performance of scAI and its comparison with MOFA using eight simulated datasets. **a** 2D visualization of cells by applying UMAP to scRNA-seq, scATAC-seq, and aggregated scATAC-seq data obtained from scAI. Each row shows one example of each scenario from the simulated datasets. Cells are colored based on their true labels. **b** Cells are visualized by VscAI. **c** Accuracy of scAI (evaluated by AUC) in reconstructing cell loading (blue color), gene loading (orange color), and locus loading (yellow color) matrices, respectively. For each scenario, we generated a set of simulated data using five different parameters, which are indicated on the x-labels. The numbers outside and inside the brackets represent the parameters in the simulated scRNA-seq and scATAC-seq data, respectively. We applied scAI to each dataset 10 times with different seeds and then calculated the average AUCs with respect to the ground truth of the loading matrices. Datasets 5 and 6 were generated based on real datasets, which do not have ground truth of the gene/locus loading matrices. **d** Variance explained by each latent factor (LF) using MOFA. **e** Comparison of the accuracy (evaluated by normalized mutual information, NMI) of scAI and MOFA in identifying cell clusters

First, we compared the visualization of cells using the scRNA-seq data, scATAC-seq data, and aggregated scATAC-seq data, respectively (Fig. 2a). Due to the inherent sparsity and noise in the data, the cells were not well separated in the scRNA-seq data and the scATAC-seq data using Uniform Manifold Approximation and Projection [29] (UMAP) (Fig. 2a) and t-SNE (Additional file 2: Figure S1), in particular for datasets 5 and 6. However, the cell subpopulations were clearly distinguishable in the low-dimensional space when using the aggregated scATAC-seq data generated by scAI for all eight different scenarios (Fig. 2a). In addition, the cell subpopulations were well separated when visualized by VscAI, which embedded cells in two dimensions by leveraging the information from both scRNA-seq and scATAC-seq data (Fig. 2b). For dataset 3 and dataset 4, in which one cluster was missing in either the transcriptomic or the epigenomic data alone, scAI was able to reveal all the anticipated clusters. For example, in dataset 4, only four clusters were revealed in the scRNA-seq data, but five clusters were embedded in the scATAC-seq data (the fourth row of Fig. 2a). Without the addition of the scATAC-seq information, four clusters were detected (Additional file 2: Figure S2), whereas the integration of both the scRNA-seq and the scATAC-seq data revealed five clusters. In the first five datasets, the cell states are discrete whereas dataset 6 depicts a continuous transition process at five different time points. The continuous transitions in these five cell states were well characterized by scAI with the aggregated scATAC-seq data but could not be captured by using only the sparse scATAC-seq data with UMAP (the sixth row of Fig. 2a) and t-SNE (Additional file 2: Figure S1). For the datasets 7 and 8 with imbalanced cluster sizes, scAI accurately revealed all the expected clusters. In particular, three cell clusters were observed in the low-dimensional space of both scATAC-seq and aggregated scATAC-seq data in the dataset 8 (the eighth row of Fig. 2a). However, five cell clusters were well distinguished after integrating with scRNA-seq data, as shown in the VscAI space (the eighth row of Fig. 2b).

Next, we used the area under receiver operating characteristic curve (AUC) to quantitively evaluate the accuracy of scAI in reconstructing cell loading matrix *H*, gene loading matrix *W*_1_, and locus loading matrix *W*_2_, which were used for identifying cell clusters, factor-specific genes, and loci in the downstream analyses, respectively. scAI was found to perform robustly and accurately with different sparsity levels and noise levels (Fig. 2c). For example, even with the sparsity levels of *X*_1_ and *X*_2_ at 98% and 99.6% in dataset 1, and 79.4% and 97.5% in dataset 5, scAI was able to reconstruct these loading matrices with high accuracy (Fig. 2c).

Moreover, to study whether stronger noise or the initial data with less discriminative patterns have effects on the performance of scAI, we added stronger noise and sparsity levels, and also made the initial data less discriminative among clusters by increasing the parameter value *coph*, on the simulation dataset 8. We found that the noise levels and parameter *coph* values have little effects on the reconstructed loading matrices. The sparsity level affects the performance if it is larger than some threshold (e.g., the sparsity of scRNA-seq and scATAC-seq data is larger than 98.9% and 99.5%, respectively), as shown in Additional file 2: Figure S3.

Finally, we applied MOFA [17], a multiomics data integration model designed for bulk data and single-cell data, to the eight datasets (Fig. 2d, e). MOFA decomposes multiomics data matrices into several weight matrices and one factor matrix using a statistically generalized principal component analysis method. For all the datasets except for dataset 7, the factors learned by MOFA only accounted for the variability of the scRNA-seq data, and could not capture the variance in the scATAC-seq data (Fig. 2d). We compared scAI with MOFA on cell clustering (Fig. 2e), finding MOFA does not perform as well as scAI for these simulation datasets (Fig. 2e).

The analysis on simulation data indicates scAI’s potential in aggregating scATAC-seq data, identifying important genes and loci, and uncovering discrete and continuous cell states in single-cell transcriptomic and epigenomic data with inherently high sparsity and noise levels.

### Identifying subpopulations with subtle transcriptomic differences but strong chromatin accessibility differences

To evaluate scAI in capturing cell subpopulations in complex tissues, we analyzed 8837 cells from mammalian kidney using the paired chromatin accessibility and transcriptome data [6]. In a previous study, a semi-supervised clustering method was applied to the scRNA-seq data, and then, aggregated epigenomic profiles were generated based on the identified cell clusters [6]. As such, the cellular heterogeneity induced by epigenetics was unable to be captured in this method.

**Fig. 3:**
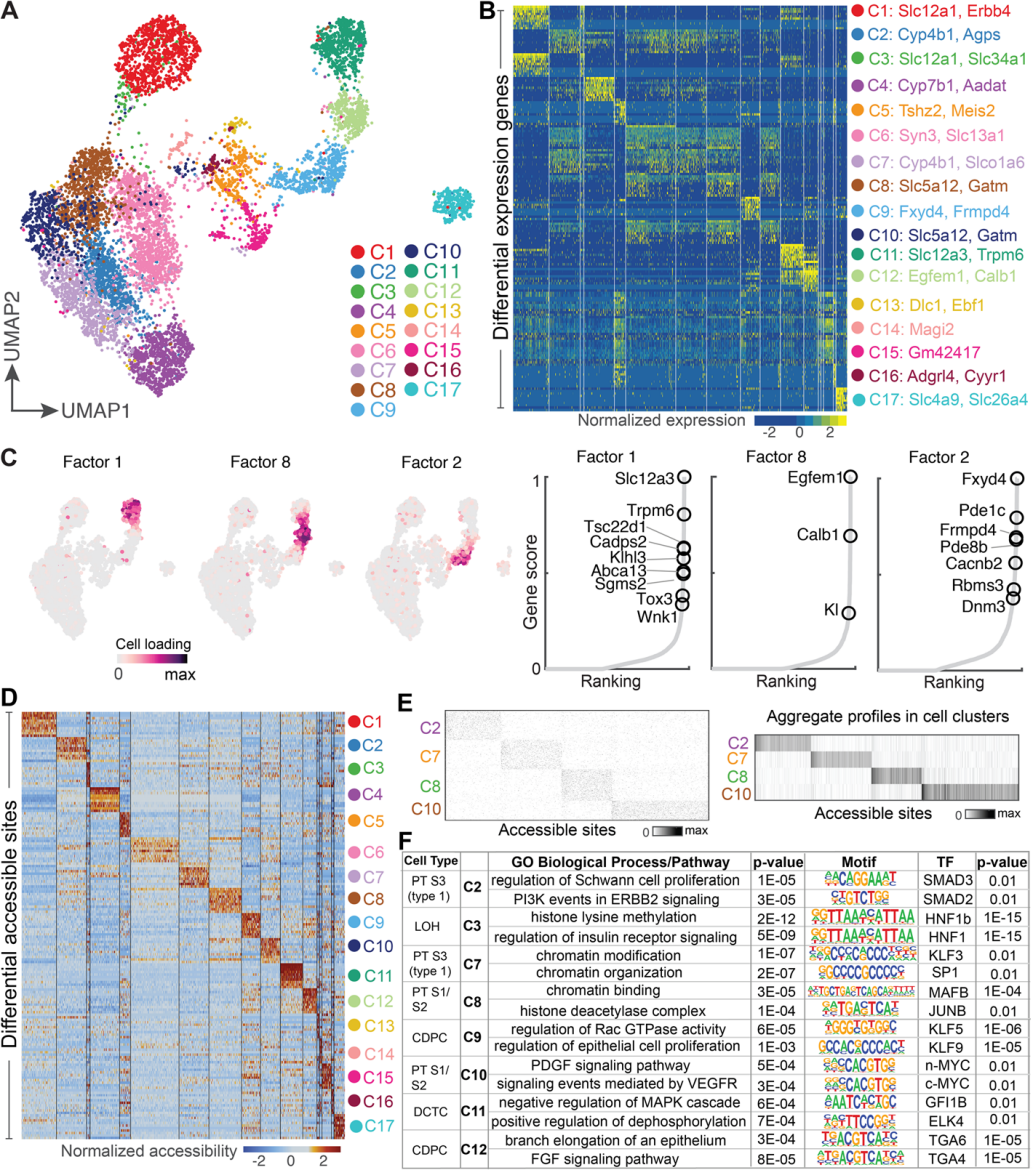
Identifying new epigenomics-induced subpopulations by simultaneously analyzing transcriptomic and epigenomic profiles in mouse kidney. **a** UMAP visualization of cells, which are colored by the inferred subpopulations. **b** Heatmap of differentially expressed genes. For each cluster, the top 10 marker genes and their relative expression levels are shown. Selected genes for each cluster are color-coded and shown on the right. **c** UMAP plots show the cell cluster-specific patterns of the identified factors (left), and the ranking plots show the top marker genes in the corresponding factors (right). In the projected factor pattern plots, cells are colored based on the loading values in the factor from the inferred cell loading matrix. In gene ranking plots, genes are ranked based on the gene scores in the factor from the gene loading matrix. Labeled genes are representative markers. **d** Heatmap showing the relative chromatin accessibility of cluster-specific loci. **e** Heatmap of the raw chromatin accessibility of individual cells (left) and the average chromatin accessibility of cell clusters (including C2, C7, C8, and C10) (right) using differential accessible loci among the cell clusters. **f** Regulatory information of eight identified cell clusters. The identities of these subpopulations were shown on the most left

scAI identified 17 subpopulations with either distinct gene expression or chromatin accessibility profiles with the default resolution parameter equaling 1 (see the “Methods” section; Fig. 3a, b, d; Additional file 1). Compared to the original findings [6], our integrative analysis of transcriptomic and chromatin accessibility profiles indicated that the known cell types such as Collecting Duct Principal Cells (CDPC) were much more heterogeneous. We identified two subpopulations of CDPC (C9 and C12, Additional file 2: Figure S4a) that were captured by factor 2 and factor 8, respectively (Fig. 3c). Gene loading analysis of these two factors revealed that Fxyd4 and Frmpd4 are the specific markers of C9, while Egfem1 and Calb1 are the specific makers of C12 (Fig. 3c, and Additional file 2: Figure S4b and c). Importantly, while some identified subpopulations showed only subtle differences in their transcriptomic profiles, they exhibited distinct patterns in their epigenomic profiles (Fig. 3b, d). For example, C2 and C7 (subpopulations of proximal tubule S3 cells (type 1)), and C8 and C10 (subpopulations of proximal tubule S1/S2 cells) have similar gene expression profiles (Fig. 3b), but, exhibit strong differential accessibility patterns (Fig. 3e). The average signals of each locus across cells in each subpopulation are significantly different (Fig. 3e).

To further characterize these differential accessible loci and identify the specific transcriptional regulatory mechanisms of these epigenetics-induced subpopulations, we performed gene ontology enrichment and motif discovery analysis using GREAT and HOMER, respectively (Fig. 3f). Notably, for the two subpopulations C8 and C10 of proximal tubule S1/S2 cells, the C8-specific accessible loci were related to the chromatin binding and histone deacetylase complex, and were further enriched for binding motifs of MAFB and JUNB, both of which are known regulators of proximal tubule development [30]. Differential accessible loci of C10 were enriched in VEGFR signaling pathway, consistent with the role in the maintenance of tubulointerstitial integrity and the stimulation of proximal tubule cell proliferation [31].

Moreover, we applied chromVAR [32] to analyzing the differential accessible loci between C2 and C7, and C8 and C10, respectively. chromVAR calculates the bias corrected deviations in accessibility. For each motif, there is a value for each cell, which measures how different the accessibility for loci with that motif is from the expected accessibility based on the average of all the cells. By performing hierarchical clustering of the calculated deviations of top 30 most variable TFs, we found that these TFs were divided into 2 clusters, and each TF cluster was specific to 1 particular cell subpopulation, which was found to be consistent with the clustering by scAI (Additional file 2: Figure S5).

### Revealing underlying transition dynamics by analyzing transcription and chromatin accessibility simultaneously

**Fig. 4:**
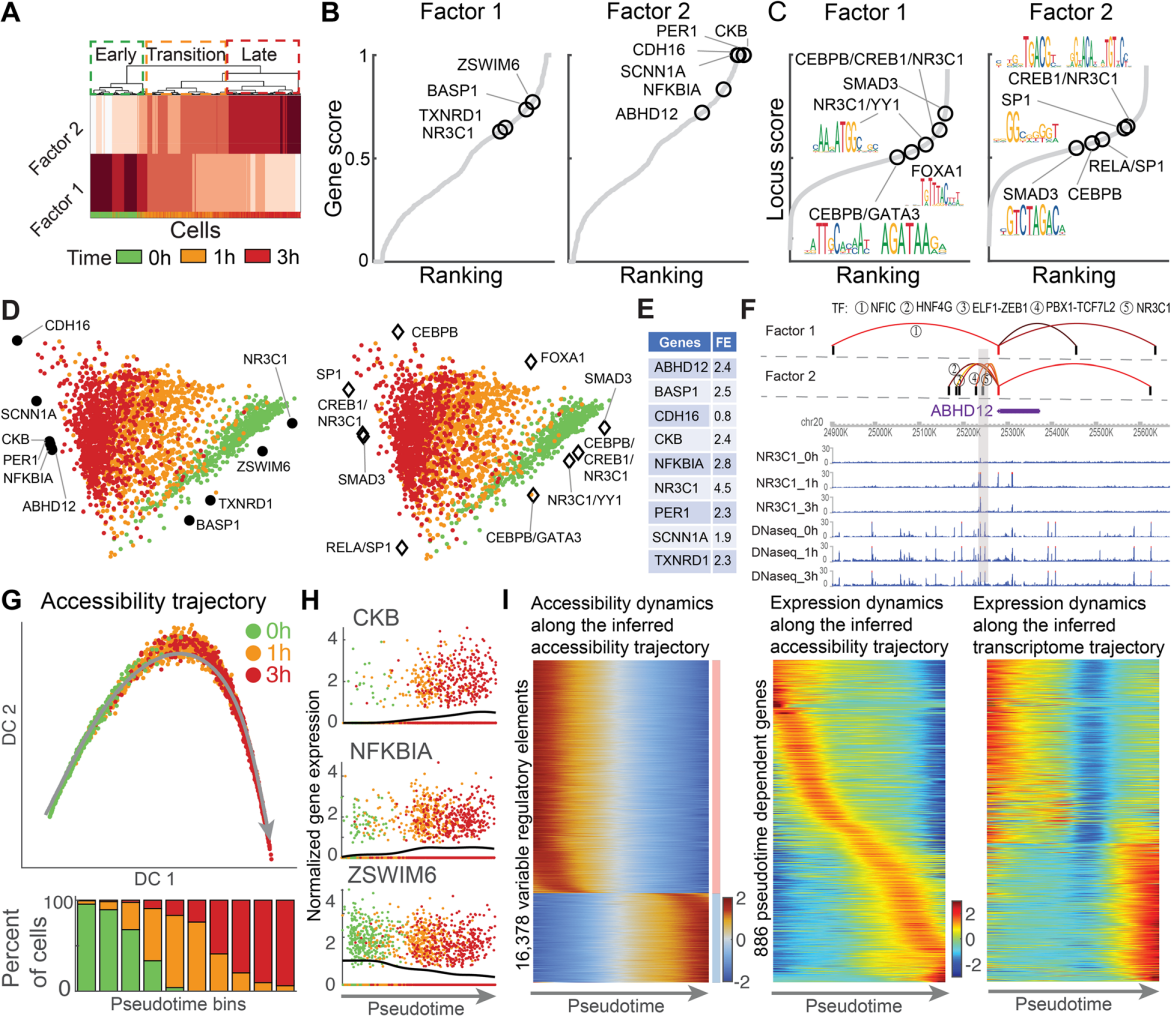
Revealing cellular heterogeneity and regulatory links of dexamethasone-treated A549 cells. **a** Heatmap of the cell loading matrix *H* obtained by scAI. Cells are ordered and divided into early, transition, and late stages based on hierarchical clustering. The bar at the bottom indicates the collection time of each cell. **b** Genes are ranked in each factor based on gene scores calculated from gene loading matrix, in which the known markers are indicated. **c** Loci are also ranked based on locus scores from locus loading matrix, in which the motifs and the corresponding logo of some TFs of the known marker genes are indicated. The binding TFs of the known marker genes and the chromosome loci of these motifs were found from hTFtarget database. **d** Visualization of cells by VscAI. Known marker genes (left panel) and motifs related with these marker genes (right panel) were projected onto the same low-dimensional space. The same motifs such as SMAD3 and NR3C1 are shown in two opposite positions, as they are enriched in different loci. These loci were located within 10 kb of marker genes’ regulatory regions, which were extracted from the database (http://bioinfo.life.hust.edu.cn/hTFtarget/) in lung tissue. Here, we visualized the motifs instead of individual loci for easier understanding. **e** The fold enrichment (FE) values of inferred regulatory links of the known markers, which were validated by the hTFtarget database. **f** Inferred regulatory links of gene ABHD12 for each factor and the epigenome browser visualization of DNase-seq data and NR3C1 ChIP-seq data derived from chromatin regions near TSS of ABHD12. The red stem represents the TSS region of the gene, and the black stem represents each locus. Five regulators which correspond to the inferred regulatory links were indicated. The gray region shows the distinct regulatory links (regulated by NR3C1) between 0 h and 1 and 3 h. **g** The pseudotemporal chromatin accessibility trajectory was inferred with the aggregated scATAC-seq data. Cells were visualized in the first two diffusion components (DCs). The gray line is the fitted principal curve. Bottom: the percentages of cells at the three time points during the inferred pseudotime, which was divided into 10 bins. **h** Inferred pseudotime of three key genes. The black line indicates the fitted expression levels using cubic splines. **i** Left: “Rolling wave” plot shows the normalized smoothed accessibility data for the pseudotime-dependent accessible loci clustered into two groups. Middle: the normalized smoothed gene expression data for the pseudotime-dependent genes along the inferred accessibility trajectory using the aggregated scATAC-seq data. Loci and genes are ordered based on the onset of activation. Right: the corresponding gene dynamics along the cellular trajectory inferred only using scRNA-seq data

Next, we applied scAI to data from lung adenocarcinoma-derived A549 cells after 0, 1, and 3 h of 100 nM dexamethasone (DEX) treatment, including scRNA-seq and scATAC-seq data from 2641 co-assayed cells [6]. scAI revealed two factors, where factor 1 was enriched with cells from 0 h and factor 2 was enriched with cells from 3 h (Fig. 4a). Factor-specific genes and loci were identified by analyzing the gene and locus loading matrices (Fig. 4b). Among them, known markers of glucocorticoid receptor (GR) activation [33–35] (e.g., CKB and NKFBIA) were enriched in factor 2, and markers of early events after treatment [36] (e.g., ZSWIM6 and NR3C1) were enriched in factor 1. We collected TFs of these known markers from hTFtarget database (http://bioinfo.life.hust.edu.cn/hTFtarget/). Interestingly, the TF motifs, such as FOXA1 [37], CEBPB [38], CREB1, NR3C1, SP1, and GATA3 [39], also had high enrichment scores in the inferred factors (Fig. 4c), in agreement with that these motifs are key transcriptional factors of GR activation markers [40]. Particularly, CEBPB binding was shown positively associated with early GR binding [41], and GR binds near CREB1 binding sites that makes enhancer chromatin structure more accessible [42]. In the low-dimensional space visualized by VscAI, markers of early events, such as ZSWIM6 and NR3C1, were located near cells from 0 h, while markers of GR activation, such as CKB, NKFBIA, and ABHD12, were located near cells from 3 h (Fig. 4d), providing a direct and intuitive way to interpret the data.

To systematically assess the top ranked genes and loci in the identified factors, we performed pathway enrichment analysis of genes with MSigDB [43] and loci with GREAT [44]. As expected, several processes relevant to GR activation were uncovered, such as the “neurotrophin signaling pathway,” a pathway previously reported to have a direct effect on GR function [45]. The “Fc epsilon RI signaling pathway” was enriched in factor 2 (Additional file 2: Figure S6a), which is in good agreement with that the reduction of Fc epsilon RI levels might be one of the favorable anti-allergic functions of glucocorticoids in mice [46]. Furthermore, processes such as “genes involved in glycogen breakdown (glycogenolysis),” “genes involved in glycerophospholipid biosynthesis,” and “pentose and glucuronate interconversions” were enriched in the nearby genes of the factor-specific loci (Additional file 2: Figure S6b).

While the DEX treatment of A549 cells is known to increase both transcription and promoter accessibility for markers of GR activation [6], little is known on the regulatory relationships. We inferred regulatory links between cis-regulatory elements and target marker genes using perturbation-based correlation analysis and further identified bounded TFs that regulate target marker genes using nonnegative least square regression (see the “Methods” section). To assess the accuracy of the inference, we evaluated whether these regulatory relationships were enriched in an independent database of TF-target relationships for human (hTFtarget, http://bioinfo.life.hust.edu.cn/hTFtarget/) (see the “Methods” section). Encouragingly, high enrichment of the inferred regulatory relationships for the key markers of GR activation was observed (Fig. 4e), and the inferred regulatory relationships were able to be validated using ChIP-seq and DNase-seq data from ENCODE (https://www.encodeproject.org/). For the GR activation marker ABHD12 that was highly enriched in factor 2, we identified distinct regulatory links between factor 1 (enriched with cells from 0 h) and factor 2 (enriched with cells from 3 h). Among its regulators, the glucocorticoid receptor NR3C1 was revealed in factor 2 (Fig. 4f). Visualizing the chromatin signals of ChIP-seq data of NR3C1 and DNase-seq data using WashU Epigenome Browser (https://epigenomegateway.wustl.edu/browser), we found that most cis-regulatory elements are located in the open regions of the DNase-seq data, and that NR3C1 exhibits signals within 50 kb of the transcription start site (TSS) of ABHD12 at 1 and 3 h but no signals at 0 h in the ChIP-seq data. This is consistent with our prediction on the regulation between NR3C1 and ABHD12 existing in factor 2, but not in factor 1.

scAI provides an unsupervised way to aggregate sparse scATAC-seq data from similar cells through iterative refinement, which facilitates and enhances the direct analysis of scATAC-seq data. We next assess the performance of the aggregated scATAC-seq data in comparison with the raw scATAC-seq or scRNA data, in terms of the identification of cell states, the low-dimensional visualization of cells, and the reconstruction of the pseudotemporal dynamics. The previous study [6] identified two clusters that comprised a group of untreated cells and a group of DEX-treated cells, in which treated cells collected from 1 and 3 h form one cluster. Our analysis recovered three cell states, including an early state enriched by cells from 0 h, a transition state enriched by cells from 1 h, and a late state enriched by cells from 3 h (Fig. 4a). Due to the high sparsity (96.8% for scRNA-seq and 99.2% for scATAC-seq) and the near-binary nature of the scATAC-seq data, dimension reduction methods, such as t-SNE, were found to fail to distinguish the different cell states (Additional file 2: Figure S6c). However, scAI uncovered distinct cell subpopulations, as seen in the low-dimensional space, based on the aggregated data (Additional file 2: Figure S6c).

We next study the pseudotemporal dynamics of A549 cells using our previously developed method scEpath [47]. Compared to the trajectory inferred using only the scRNA-seq data, which lacks well-characterized GR activation trends for cells measured at three different time points (Additional file 2: Figure S6d), a clear and consistent trajectory was inferred when using the aggregated scATAC-seq data (Fig. 4g, h). We identified pseudotime-dependent genes and loci that were significantly changed along the inferred trajectories. The pseudotemporal dynamics of these genes along the trajectory inferred using only the scRNA-seq data were found to be discontinuous, in contrast to the aggregated scATAC-seq data obtained from scAI led to continuous trajectory (Fig. 4i). Previously, we used the measure scEnergy to quantify the developmental process [47]. Here, we found no significant differences in the single-cell energies between different time points when only using the scRNA-seq data. However, significantly decreased scEnergy values were seen during treatment according to the aggregated scATAC-seq data (Additional file 2: Figure S6e).

Overall, the aggregated scATAC-seq data by scAI can better characterize the dynamics of DEX treatment, and scAI suggests new mechanisms regarding the GR activation process in DEX-treated A549 cells, including a transition state and differential cis-regulatory relationships.

### Uncovering coordinated changes in the transcriptome and DNA methylation along a differentiation trajectory

To study data with simultaneous single-cell methylome and transcriptome sequencing [3, 8, 48], we applied scAI to a dataset obtained from 77 mouse embryonic stem cells (mESCs), including 13 cells cultured in “2i” media and 64 serum-grown cells, which were profiled by parallel single-cell methylation and transcriptome sequencing technique scM&T-seq [3]. The DNA methylation levels were characterized in three different genomic contexts, including CpG islands, promoters, and enhancers, which are usually linked to transcriptional repression [49, 50].

**Fig. 5:**
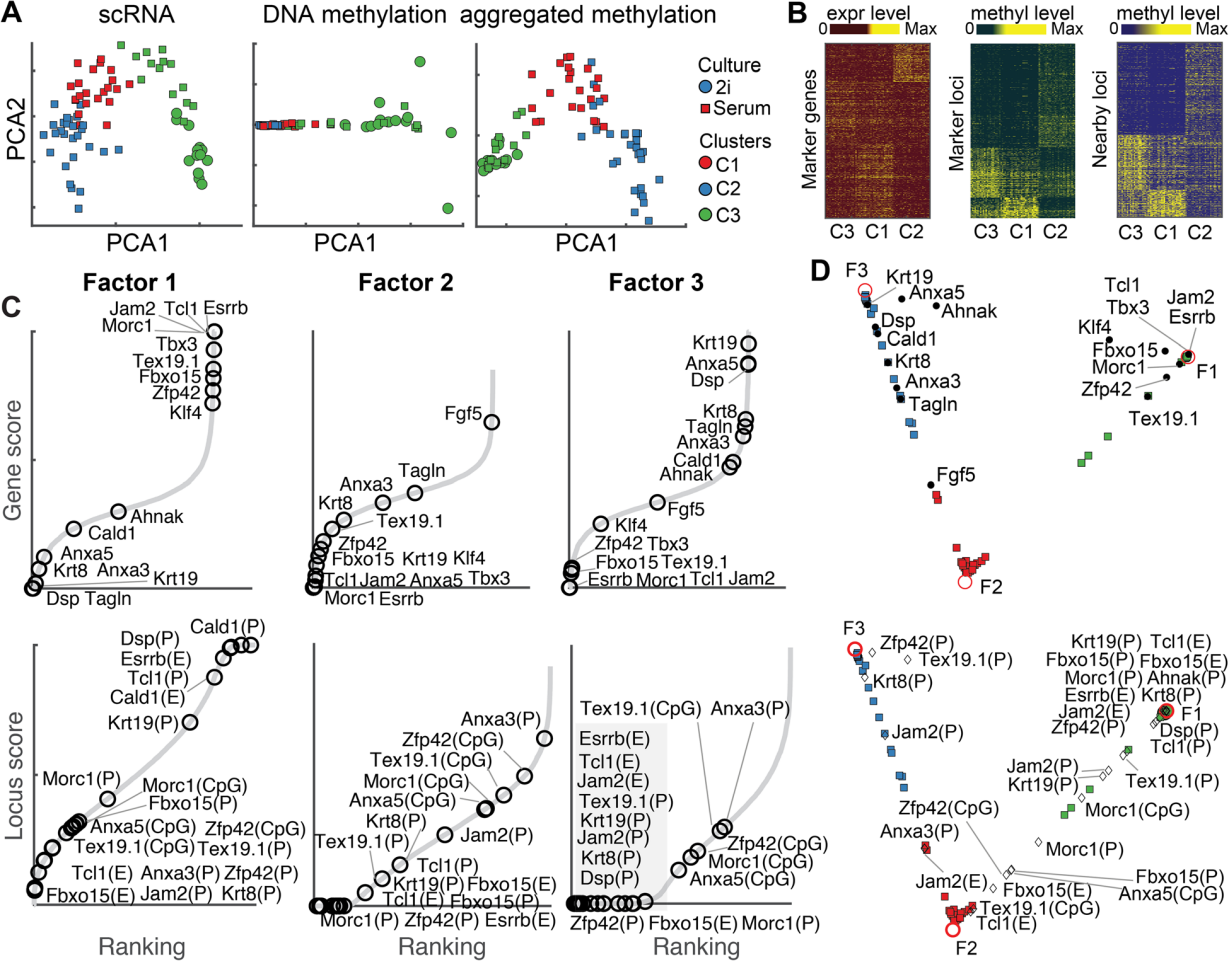
Uncovering coordinated changes between the transcriptome and DNA methylation within an embryonic stem cell differentiation trajectory. **a** Comparisons of principal component analyses (PCA) of scRNA-seq data, single-cell DNA methylation data, and aggregated single-cell DNA methylation data learned by scAI. Cells are colored based on the cell subpopulations identified using scAI. Marker shapes denote the culture conditions. **b** Heatmap of the expression level and methylation level of cluster-specific marker genes (left), loci (middle), and loci within 500 kb of the TSS of marker genes (right) in the three cell clusters. **c** Genes and loci are ranked based on their enrichment scores in each factor. Labeled genes (top) are known pluripotency markers or differentiation markers. Labeled loci (bottom), including CpG sites, enhancers, and promoters, are located within 500 kb of the TSS of marker genes in each factor. **d** VscAI visualization of cells and known pluripotency markers and differentiation markers derived from transcriptome (top) and DNA methylation (bottom) data

Because DNA methylation data are sparse and binary, direct dimensional reduction may fail to capture cell subpopulations (Fig. 5a). scAI was able to distinguish cell subpopulations after aggregation (Fig. 5a), showing three subpopulations, C1, C2, and C3. Among them, C3 was captured by factor 1 with cells cultured in “2i” media and a few serum-grown cells, while C1 and C2 were captured by factors 2 and 3, respectively, with other serum-grown cells (Additional file 2: Figure S7).

Based on the top gene and locus loadings in each factor, we identified 688, 877 and 422 marker genes and 2164, 953 and 4461 differential methylated loci in C1, C2, and C3, respectively, with distinct gene expression and methylation patterns among these three groups (Fig. 5b). Moreover, methylation levels of loci near marker genes also showed group-specific patterns (Fig. 5b). Several known pluripotency markers (e.g., Essrb, Tcl1, Tbx3, Fbxo15, and Zpf42) exhibited the highest gene enrichment scores in factor 1 but the lowest gene enrichment scores in factors 2 and 3. In contrast, differentiation markers, such as Krt8, Tagln, and Krt19, exhibited higher gene enrichment scores in factor 3 but lower enrichment scores in factors 1 and 2 (Fig. 5c). Factor 2 exhibited an intermediate state with a relatively low expression level of both pluripotency and differentiation markers. Interestingly, several new marker genes of this intermediate state were observed, such as Fgf5, an early differentiation marker involved in neural differentiation in human embryonic stem cells [51]. Factor-specific loci located in the CpG, promoter, and enhancer regions of marker genes are also shown in Fig. 5c. The pluripotency markers Essrb and Tcl1 had higher gene enrichment scores, and their corresponding CpG, promoter, and enhancer regions had higher locus enrichment scores in factor 1. This relationship is consistent with the fact that some DNA methylation located in the CpG, promoter, and enhancer regions exhibit a negative relationship with the expression level of target genes.

A continuous differentiation trajectory, which was characterized by the differentiation of naïve pluripotent cells (NPCs) into primed pluripotent cells and ultimately into differentiated cells (DCs), was observed using VscAI (Fig. 5d). Additionally, the embedded genes and factors showed how specific genes and factors contribute to the differentiation trajectory. For example, pluripotency markers, such as Zpf42, Tex19.1, Fbxo15 Morc1, Jam2, and Esrrb [52, 53], were visually close to factor 1, while differentiation markers, such as Krt19 and Krt8 [54], were close to factor 3 (Fig. 5d). Interestingly, although both pluripotency and differentiation markers were not highly expressed in the early differentiated state in factor 2, some methylated loci of these markers (e.g., CpG regions of Zfp42 and Tex19.1, enhancer region of Jam2 and Tcl1, and promoter region of Anxa3) were enriched in factor 2 (Fig. 5d). These observations might be because their other regions (CpG, enhancer, or promoter) are methylated or DNA methylation is not the main driven force for transcriptional silencing. Overall, scAI shows coordinated changes between transcriptome and DNA methylation along the differentiation process.

### Comparison with three multiomics data integration methods

We next compared scAI with three recent single-cell integration methods, MOFA [17], Seurat (version 3) [22], and LIGER [23], on A549 and kidney datasets. Similar to the observations on the simulation datasets (Fig. 2d), MOFA cannot capture the variations in the scATAC-seq data as the variances explained by the learned factors in the scATAC-seq data were nearly zero (Additional file 1: Supplementary methods (*Details of data analysis by MOFA*) and Additional file 2: Figure S8a-e). While Seurat and LIGER were designed for connecting cells measured in different experiments, we applied them to the two co-assayed single-cell multiomics data to test whether they are able to make links between co-assayed cells. We assessed the comparison using two metrics: (a) entropy of batch mixing and (b) silhouette coefficient. The entropy of batch mixing measures the uniformity of mixing for two samples in the aligned space [55], for which scRNA-seq and scATAC-seq profiles were treated as two batches, and a higher entropy value means better alignment. The silhouette coefficient quantifies the separation between cell groups using distance matrices calculated from a low-dimensional space [55], for which cell group labels were taken from the original study [6] and a higher silhouette coefficient indicates better preservation of the differences and structures between different cell groups.

**Fig. 6:**
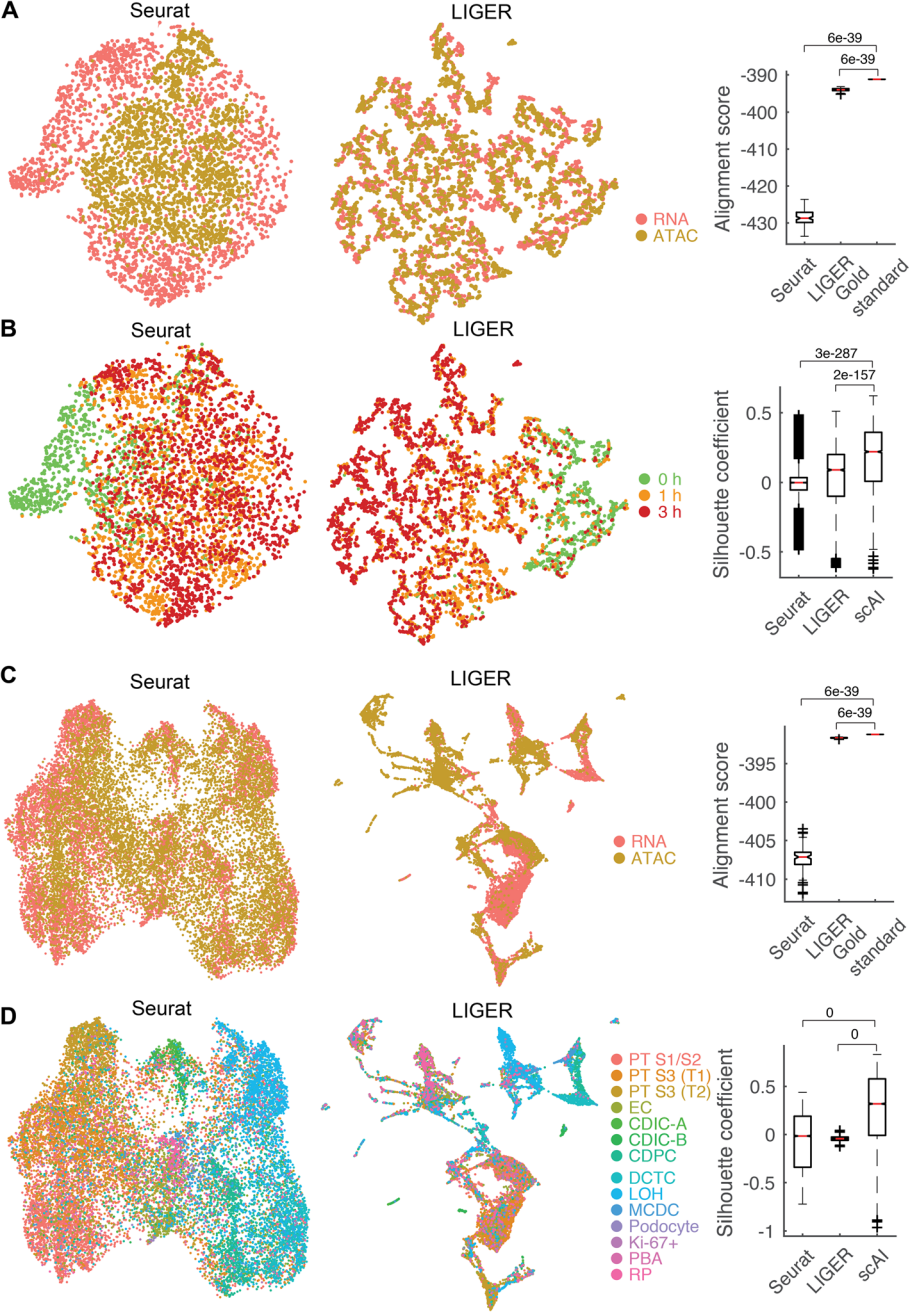
Comparisons with multiomics data integration methods. **a** t-SNE visualizations of scRNA and scATAC-seq data from co-assayed A549 cells, colored by measurements (RNA vs. ATAC) after integration with Seurat (left) and LIGER (middle). Right panel: comparisons of alignment score (quantified by the entropy of batch mixing) from perfect alignment (termed as gold-standard) with that computed from the aligned t-SNE space using Seurat and LIGER. *p* values are from the Wilcoxon rank-sum test. **b** Cells are colored by the data collection times. Right panel: comparisons of silhouette coefficient computed from the t-SNE coordinates of each cell generated by scAI with that computed from the aligned t-SNE space using Seurat and LIGER. **c**, **d** UMAP visualizations of scRNA and scATAC-seq data from co-assayed mouse kidney cells colored by measurements (RNA vs. ATAC) (**c**) and published cell labels (**d**) after integration with Seurat and LIGER. The alignment score and silhouette coefficient were also shown

The t-SNE analysis shows the co-assayed cells were aligned better by LIGER than Seurat when the two methods were applied to A549 dataset (Fig. 6a). This observation is further confirmed by computing the entropy of the batch mixing based on the aligned t-SNE space. We also computed the entropy of perfect alignment (i.e., the t-SNE coordinates of each pair of co-assayed cells are the same), and found that LIGER showed higher entropy value than Seurat, but lower entropy than the perfect alignment (Fig. 6a). In addition, we explored the quality of time point-based grouping of cells on the t-SNE space. Cells from 1 and 3 h were mixed together on the t-SNE space generated by Seurat, while there was a gradual change of cells from 0 to 3 h on the t-SNE space generated by LIGER (Fig. 6b). We also performed t-SNE on the cell loading matrix inferred by scAI (Additional file 2: Figure S8f), and found that scAI was able to capture the gradual change of cells transitioning from 0 to 3 h. Quantitatively, scAI produced significantly higher silhouette coefficients than those from both Seurat and LIGER (Fig. 6b).

In the kidney dataset, by computing the entropy of the batch mixing based on the aligned UMAP space, we observed significantly lower entropy of Seurat and LIGER than that of the perfect alignment (Fig. 6c). We then also calculated the silhouette coefficient using the UMAP space for all three methods (Fig. 6d and Additional file 2: Figure S8f). Again, significantly higher silhouette coefficients were observed in scAI, in comparison with those in Seurat and LIGER (Fig. 6d). Together, these results suggest that integration methods designed for measurements in different cells (e.g., Seurat and LIGER) may not accurately identify correspondences between the co-assayed cells, leading to errors in downstream analysis, and the integration of parallel single-cell omics data needs specialized methods, such as scAI, to deal with the epigenomic data with inherently high sparsity and to better preserve intrinsic differences between cell subpopulations.

### Comparison with methods using single omics data

To evaluate the significance of the parallel profiling of multiomics over single omics data, we compared scAI with methods that use only transcriptomic data or only epigenomic data on both simulation and real datasets. Specifically, we compared scAI with two methods designed for only scRNA-seq data, including Seurat and SC3 [56], and two methods designed for only scATAC-seq data, including Signac (https://satijalab.org/signac/) and scABC [57]. On simulation datasets, we evaluated the performance of cell clustering using normalized mutual information (NMI). On real datasets, we compared the clustering based on those four methods with prior labels using UMAP.

On simulation datasets, we observed comparable NMI values between scAI and SC3, but slightly lower values of Seurat (Additional file 2: Figure S9). For the clustering of scATAC-seq data, both Signac and scABC showed significantly lower NMI values compared to those by scAI using both scRNA-seq and scATAC-seq data. On A549 real datasets, by visualizing cells in UMAP, we found that both Seurat and SC3 were unable to detect the transition stage and distinguish cells from 1 and 3 h. Cell clusters identified by Signac and scABC using scATAC-seq data alone were found to be inconsistent with the prior labels (Additional file 2: Figure S10a). On kidney dataset, Seurat was unable to distinguish the DCTC cells and CDPC cells, and Signac and scABC were also producing clusters inconsistent with prior labels (Additional file 2: Figure S10b). On mESC dataset, while both Seurat and SC3 correctly identified the cell subpopulations, clusters identified by Signac and scABC also mixed together in UMAP (Additional file 2: Figure S10c). Overall, scAI is able to consistently identify the expected clusters and also the clusters with subtle transcriptomic differences but strong chromatin accessibility differences (as shown in kidney dataset), showing the importance of integrating parallel single-cell multiomics data.

## Discussion

A key challenge in analyzing single-cell multiomics data is to integrate and characterize multiple types of measurements coherently in a biologically meaningful manner. Often, different components in such multiomics measurements exhibit fundamentally different features, for example, some data are binary and inherently sparse whereas the other are more akin to a continuous distribution after normalization [9]. We presented an unsupervised method, scAI, for integrating scRNA-seq data and single-cell chromatin accessibility or DNA methylation data obtained from the same single cells. scAI learned three sets of low-dimensional representations of high-dimensional data: the gene, locus, and cell loading matrices describing the relative contributions of genes, loci, and cells in the inferred factors, and the cell-cell similarity matrix used for aggregating sparse epigenomic data. These learned low-rank matrices allow direct identification of cell subpopulations/states and the associated marker genes or loci that characterize each subpopulation, and provide a convenient visualization of cells, genes, and loci in the same low-dimensional space. Simultaneous analyses of the gene and locus loading matrices enable inference of the regulatory relationships between the transcriptome and the epigenome. Together, scAI provides an effective and biologically meaningful way to dissect heterogeneous single cells from both transcriptomic and epigenomic layers.

The sparse and binary nature of single-cell ATAC-seq or DNA methylation data poses a computational challenge in analysis. Aggregation has been a primary method for analyzing such data [20]. For example, Cicero, an algorithm used for predicting cis-regulatory DNA interactions from scATAC-seq data, aggregates similar cells using a *k*-nearest neighbors approach based on a reduced dimensional space (e.g., t-SNE and DDRTree) [58]. However, as shown in our simulated data and real co-assayed data, dimensional reduction techniques often fail to capture cell similarity from the chromatin accessibility or DNA methylation profiles. To deal with this difficulty, scAI first combines sparse epigenomic profiles from subgroups of cells that exhibit similar gene expression *and* epigenomic profiles. These similar cells are analyzed by learning a cell-cell similarity matrix based on a matrix factorization model. The differences between such learned similarity matrix and the similarity matrix computed using only scRNA-seq or only aggregated scATAC-seq data were also investigated (Additional file 1 (*Comparison of cell-cell similarity matrix*) and Additional file 2: Figure S11 and Figure S12). Our iterative and unsupervised approach combines information from multiple-omics layers by taking advantages of the strengths in optimization models.

To investigate whether scAI might make epigenomic data seemingly more distinct than they actually are, we employed the following two strategies on simulation datasets. Firstly, we compared the aggregated scATAC-seq data obtained from scAI with the raw ATAC-seq data prior to making them sparse and binarization (termed as bulk ATAC-seq data hereafter) in two ways: the direct visualization of loci patterns using heatmap and the low-dimensional visualization of cells using UMAP. The bulk ATAC-seq data and the aggregated scATAC-seq data were found to exhibit the same loci patterns (Additional file 2: Figure S13a). Both bulk ATAC-seq and aggregated scATAC-seq data were found to be distinct across clusters (Additional file 2: Figure S13b). These observations were consistent across all the eight simulation datasets. Secondly, we randomly permuted scATAC-seq data across all cells before applying scAI to the scRNA-seq data and the permuted scATAC-seq data. We found that the aggregated permuted scATAC-seq data were still distinct across clusters in some cases in UMAP (Additional file 2: Figure S13c), partly because there were still differential accessibility patterns across these clusters after permutation (Additional file 2: Figure S13d). Next, we considered an extreme case where all the values of scATAC-seq data are equal and found aggregated scATAC-seq data did not produce any artificial clusters, partly due to our normalization strategy in which scAI aggregates scATAC-seq profile after normalizing Z°R with the sum of each column equaling 1. On the other hand, scAI is able to identify cell clusters with high accuracy on all simulation datasets (Fig. 2e). Our analysis suggests scAI robustly maintains cellular heterogeneity within *and* between different subpopulations when it enhances epigenomic signals.

To investigate whether scAI introduces high portion of false positives during differential accessibility analysis using aggregated scATAC-seq data, we calculated the percentage of false positive differential accessible loci based on the aggregated scATAC-seq data by comparing them to the differential accessible loci identified using the bulk ATAC-seq data. Specifically, the percentage of false positives was defined as the percentage of differential accessible loci that were not in the set of differential accessible loci identified using the bulk ATAC-seq data. We adopt the Wilcoxon rank sum test for accessibility of cells in each subpopulation and the remaining cells. We found that the percentages of false positive differential accessible loci were less than 7% on simulation datasets (Additional file 2: Figure S14). A direct visualization for the datasets 7 and 8 with imbalanced cluster size shows consistent loci patterns and highly overlapped differential accessible loci between the aggregated scATAC-seq data and bulk ATAC-seq data (Additional file 2: Figure S15). These results suggest that the aggregation strategy has a good control of false positives for differential accessibility analysis.

The single-cell multiomics data are sparse and have large amounts of missing values. The scRNA-seq data have two states: non-zero and zero values. The zero values might be either non-expressed values or due to dropout events [59]. The single-cell methylation data have three states: methylated, unmethylated, and missing values. While replacing missing values by zeros and adopting a model that can potentially impute the missing values, a strategy used in scAI, might improve downstream analysis due to the fact that the large portions of missing values contain true zero values, such approach likely has several limitations. First, it might introduce false signals when the missing values might actually correspond to non-zero signals. Second, such approach cannot distinguish methylated and missing states for the DNA methylation data. One way to address such difficulty is to throw away the missing values, which is particularly useful for the methylation data (e.g., scM&T-seq [3]) because it allows to distinguish methylated and missing values. One powerful approach is to use probabilistic models, such as MOFA [17] and its successor MOFA+ [19], which do not include those missing value regions when computing the likelihood. In principle, we can throw away the missing values in scAI by incorporating a binary matrix into the second term of our model (Eq. (1)), an approach similar to incomplete nonnegative matrix factorization model [60].

Comparing with recent methods, such as MOFA [17], Seurat [22], and LIGER [23], scAI is able to capture cell states with higher accuracy for the multiomics data in which only gene expression *or* chromatin accessibility may be discriminated between cell states, for example, to uncover novel cell subpopulations with distinct epigenomic profiles but similar transcriptomic profiles, as seen in the kidney dataset. Such capability of identifying cell subpopulation exhibiting only distinct epigenetic profiles will facilitate further analysis of epigenetics in controlling cell fate decision and may help to reveal important transcriptional regulatory mechanism [61]. Similar to uncovering new cell subpopulation, scAI can uncover new cell transition states induced by epigenetics as seen in the analysis of the dexamethasone-treated A549 cell dataset [6], and identify co-regulations coordinated between transcriptome and DNA methylation, as seen in the mESC dataset.

For the methods (e.g., Seurat and LIGER) that are designed for integrating single-cell data measured in different cells, in principal, they can be applied to the parallel single-cell multiomics data. However, we found that these two methods yield deficient alignment between co-assayed cells, as seen in the A549 and kidney datasets. Such alignment errors might affect downstream analysis such as inferring regulatory links. Moreover, these two methods, unlike scAI, need to transform other types of features such as chromatin accessibility or DNA methylation into gene level, which leads to limited resolution and cannot make full use of epigenomic information. As parallel single-cell multiomics data becomes more widely available, methods like scAI will be essential to make sense of this new type of data.

Parallel single-cell sequencing provides a great opportunity to infer the regulatory links between transcriptome and epigenome [9]. In this study, the regulatory links between chromatin regions and marker genes were inferred by combining the correlation analysis and the nonnegative least square regression, as seen in the A549 dataset. Because many factors such as chromatin regulators, histone modification, and the microenvironment can affect the transcriptional regulation [62], more complex and accurate models are needed to improve the accuracy of regulatory relationship inference. While it remains to be done, scAI provides a computational tool for integrating parallel single-cell omics data, including visualization, clustering, differential expression/chromatin accessibility analysis, and regulatory relationship inference.

## Conclusions

Here, we present scAI, which is one of the first computational methods for the integrative analysis of single-cell transcriptomic and epigenomic profiles that are measured in the same cell. scAI was shown to be an effective tool to characterize multiple types of measurements in a biologically meaningful manner, dissect cellular heterogeneity within both transcriptomic and epigenomic layers, and understand transcriptional regulatory mechanisms. Due to rapid development of single-cell multiomics technologies, scAI will facilitate the integrative analysis of the current and upcoming multiomics data profiled in the Human Cell Atlas as well as the Pediatric Cell Atlas [63].

## Methods

### Optimization algorithm for scAI

The optimization problem (Eq. (1)) is solved by a multiplicative update algorithm, which updates variables *W*_1_, *W*_2_, *H*, and *Z* iteratively according to the following equations (Additional file 1: Supplementary methods (*Details of scAI*) and Additional file 2: Figure S16):

$$ {W}_1^{ij}\leftarrow {W}_1^{ij}\frac{{\left({X}_1{H}^T\right)}^{ij}}{{\left({W}_1H{H}^T\right)}^{ij}} $$

$$ {W}_2^{ij}\leftarrow {W}_2^{ij}\frac{{\left({X}_2\left(Z\circ R\right){H}^T\right)}^{ij}}{{\left({W}_2H{H}^T\right)}^{ij}} $$

$$ {H}^{ij}\leftarrow {H}^{ij}\frac{{\left(\alpha {W}_1^T{X}_1+{W}_2^T{X}_2\left(Z\circ R\right)+\lambda H\left(Z+{Z}^T\right)\right)}^{ij}}{{\left(\left(\alpha {W}_1^T{W}_1+{W}_2^T{W}_2+2\lambda H{H}^T+\gamma e{e}^T\right)H\right)}^{ij}} $$

$$ {Z}^{ij}\leftarrow {Z}^{ij}\frac{{\left(\left({X}_2^T{W}_2H\right)\circ R+\lambda {H}^TH\right)}^{ij}}{{\left(\left({X}_2^T{X}_2\left(Z\circ R\right)\right)\circ R+\lambda Z\right)}^{ij}}, $$

where ${W}_I^{ij},I=1,2$ represent the entry in the *i*th row and *j*th column of *W*_1_ (*p* × *K*) and *W*_2_ (*q* × *K*). *H*^*ij*^ and *Z*^*ij*^ represent the *i*th row and the *j*th column of *H* (*K* × *n*) and *Z* (*n* × *n*). *e* (*K* × 1) represents a vector with all elements being 1. In each iteration step, *H* is scaled with the sum of each row equaling 1.

In this algorithm, we initialize *W*_1_, *W*_2_, *H*, and *Z* using a 0–1 uniform distribution and generate a binary matrix *R* using a Bernoulli distribution with a probability *s*. *α* and *λ* are parameters to balance each term, and *γ* is a parameter to control sparsity of each row of *H*. The default values for those parameters are as follows: *s* = 0.25, *α* = 1, *λ* = 10,000, and γ = 1. The rank *K* is determined by a stability-based method [28] (Additional file 1: Supplementary methods (*Rank selection*) and Additional file 2: Figure S17 and Figure S18). Since *H* is scaled by row, the entry of matrix *H* is less than 1. Thus, the magnitude of the third term is small and *λ* usually is large to ensure the importance of this term. The parameter *α* is set to be small because the magnitude of this term is usually relatively large, which does not mean that *W*_1_ *and W*_2_ are not important in the model. The parameters used in all the datasets are summarized in Additional file 2: Table S2. Robustness analysis on the parameter indicates that the overall performance of scAI is relatively robust to choices of parameter values within certain ranges (Additional file 1: Supplementary methods (*Robustness analysis*) and Additional file 2: Figure S19).

### Identification of cell subpopulations

From transcriptomic and epigenomic profiles, scAI projects cells into a cell loading matrix *H*, which is a low-dimensional representation of both profiles. The subpopulations are then identified by clustering through *H* using the Leiden community detection method [64]. Specifically, a shared nearest neighbor (SNN) graph is first constructed by calculating the *k*-nearest neighbors (20 by default) for each cell based on the matrix *H*. Then, the fraction of shared nearest neighbors between the cell and its neighbors is used as weights of the SNN graph. Next, we identify cell subpopulations by applying the Leiden algorithm [64] to the constructed SNN graph with a default resolution parameter setting of 1.

### Identification of cell subpopulation-specific marker genes and epigenomic features

After determining the cell subpopulations, we adopt a likelihood-ratio test for gene expression of cells in the *k*th cell subpopulation and cells not in the *k*th cell subpopulation. Genes are considered as the *k*th cell subpopulation-specific marker genes if (i) the *p* values are less than 0.05, (ii) the log fold-changes are higher than 0.25, and (iii) the percentage of cells with expression in the *k*th cell subpopulation is higher than 25%. Cell subpopulation specific-epigenomic features are identified using a similar approach.

### Visualization of cells, genes, and loci in a 2D space

scAI simultaneously decomposes gene expression matrix and accessibility or methylation matrix into a set of low-rank matrices, including the gene loading matrix *W*_1_, locus loading matrix *W*_2_, cell loading matrix *H*, and cell-cell similarity matrix *Z*. Based on these inferred low-dimensional representations, we simultaneously visualize cells, genes, and loci in a single two-dimensional space using similarity weighted nonnegative embedding [65]. Specifically, we first compute the coordinates of the inferred factors. *H* is smoothed by the similarity matrix *Z* using *H*_*s*_ = *H* × *Z*. Then, we compute pairwise similarity matrix *S* between factors (rows of *H*_*s*_) by cosine distance. The similarity matrix *S* is converted into a distance matrix *D* according to $D=\sqrt{2\left(1-S\right)}.$ The Sammon mapping method [27] is then used to project the distance matrix *D* onto a two-dimensional space (a matrix with *K* rows (*K* is the number of factors) and 2 columns). The values in this two-dimensional matrix are scaled (ranging from zero to one) to obtain the coordinates of factor C according to *C* = (*C*_*kx*_, *C*_*ky*_), where *C*_*kx*_ and *C*_*ky*_ represent the *x* and *y* coordinates of the *k*th factor.

Next, we compute the coordinates of cell *j* (*E* = (*E*_*jx*_, *E*_*jy*_)) in the two-dimensional space according to:

$$ {E}_{jx}=\frac{\sum_k{\left({H}^{kj}{C}_{kx}\right)}^{\alpha }}{\sum_k{\left({H}^{kj}\right)}^{\alpha }},{E}_{jy}=\frac{\sum_k{\left({H}^{kj}{C}_{ky}\right)}^{\alpha }}{\sum_k{\left({H}^{kj}\right)}^{\alpha }}, $$

where the parameter α controls how tight the allowed embedding is between the cells and the factors. The reasonable value range is from 1 to 2. Large values move the cells closer to the factors, while it may distort the data when α is higher than 2. α = 1.9 is used as default. The coordinates of cells *E* are further smoothed by the similarity matrix *Z* using *E*_*s*_ = *E* × *Z* and then are used for visualization.

Finally, we embed the marker genes and loci into the same two-dimensional space according to *W*_1_ and *W*_2_ as follows:

$$ {F}_{jx}^I=\frac{\sum_k{\left({W}_I^{jk}{C}_{kx}\right)}^{\alpha }}{\sum_k{\left({W}_I^{jk}\right)}^{\alpha }},{F}_{jy}^I=\frac{\sum_k{\left({W}_I^{jk}{C}_{ky}\right)}^{\alpha }}{\sum_k{\left({W}_I^{jk}\right)}^{\alpha }}, $$

where *I* = 1,2 represents the embedding of genes and loci, respectively. Accordingly, using this integrative dimension-reduction approach, the marker genes and loci that separate cell states alongside the cells can be visualized together to help interpretation of multiomics data in an intuitive way.

### Identification of factor-specific marker genes and epigenomic features

Using scAI, we obtain gene loading and locus loading matrices, *W*_1_ and *W*_2_, and the values in each column of *W*_1_ and *W*_2_ are respectively used to identify the genes and epigenomic features associated with each factor. To rank the gene *i* in factor *k*, we define a gene score:${S}_1^{ik}={W}_1^{ik}/\sum \limits_j{W}_1^{jk}$. Similarly, we rank the loci in each factor by defining a locus score based on *W*_2_.

To identify factor-specific marker genes and epigenomic features, we divide the genes and loci into two groups for each factor. The *z*-score is computed for each entry in each column of *W*_1_ and *W*_2_: ${z}_1^{ik}=\left({W}_1^{ik}-{\mu}_1^j\right)/{\sigma}_1^k$ and ${z}_2^{ik}=\left({W}_2^{ik}-{\mu}_2^j\right)/{\sigma}_2^k$, where ${\mu}_1^k,{\mu}_2^k$ are the average values of the *k*th column in *W*_1_ and *W*_2_, respectively, and ${\sigma}_1^k,{\sigma}_2^k$ are the corresponding standard deviations. Let *AG*_*k*_ and *AL*_*k*_ represent the sets of candidate genes and loci, respectively, associated with the *k*th factor if ${z}_1^{ik},{z}_2^{ik}$ are greater than *T* (0.5 by default). Smaller *T* value gives more features that might contain redundant information, whereas larger *T* value might leave key features out. We also divide the cells into two groups for each factor using the similar method. In more detail, we compute the *z*-score for each entry in each row of the cell loading matrix *H* by *z*^*kj*^ = (*H*^*kj*^ − *μ*^*k*^)/*σ*^*k*^. If *z*^*kj*^ is greater than *T*, cell *j* is assigned to ${C}_1^k$; otherwise, it is assigned to ${C}_2^k$. Next, using a Wilcoxon rank-sum test for the candidate genes in *AG*_*k*_ in cells in ${C}_1^k$ and ${C}_2^k$, we statistically test the differences of the candidate genes in the different cell groups. Candidate genes are considered as factor-specific marker genes if (i) the *p* values are less than 0.05, (ii) the log fold-changes are higher than 0.25, and (iii) the percentage of cells with expression in ${C}_1^k$ is greater than 25%. Factor-specific epigenomic features are identified using the similar approach.

### Inference of factor-specific transcriptional regulatory relationships

Once the factor-specific marker genes and loci are determined, we next infer the regulatory links between them. For factor *k*, the two sets *AG*_*k*_ and *AL*_*k*_ consist of the identified factor-specific marker genes and loci, respectively. For a gene *g*_*i*_ in *AG*_*k*_, we select a locus set ${L}_k^i\left(\subseteq A{L}_k\right)$, which includes loci within 500 kb of the transcription start site (TSS) of *g*_*i*_, as candidate regulatory regions for a gene *g*_*i*_. To determine whether the expression level of *g*_*i*_ is influenced by the accessible status of the candidate regions in ${L}_k^i$, we use a perturbation approach based on the correlations between the expression level and accessibility. In this approach, first, we compute the Pearson correlation *P*_1_ between the *g*_*i*_ expression level and the accessibility of each locus in ${L}_k^i$ in all cells. Second, we perturb the *g*_*i*_ expression levels by setting its expression in cells in cell group ${C}_1^k$ to 0 and then compute the weighted correlation *P*_2_ between the perturbed *g*_*i*_ expression level and the accessibility of ${L}_k^i$ in all cells with *H*_*k.*_ as its weight, where *H*_*k.*_ represents the *k*th row of *H*. Third, we set the accessibility of ${L}_k^i$ in cells in cell group ${C}_1^k$to 0 and then compute the weighted correlation *P*_3_ between the original *g*_*i*_ expression level and the perturbed accessibility of ${L}_k^i$ in all cells with *H*_*k.*_. Finally, we compute the differential correlation according to *dP*_1_ =  ∣ *P*_1_ − *P*_2_ ∣ , *dP*_2_ =  ∣ *P*_1_ − *P*_3_∣. The regulatory links between gene *g*_*i*_ and loci ${l}_k^{s_i}\subseteq {L}_k^i$ are indicated if the differential correlation of *dP*_1_ or *dP*_2_ is greater than the average value of *P*_1_ and the original correlation *P*_1_ is greater than the average value of *P*_1_.

For the identified regulatory links between genes and loci, to determine which transcription factors (TFs) regulate each gene *g*_*i*_, we first identified TF motifs enriched in the loci set ${l}_k^{s_i}$ using chromVAR [32]. When running chromVAR using default parameters, the raw scATAC-seq data matrix of all loci was used as an input. Then, we regressed the gene expression level ${E}_{C_1^k}^i$ of each gene across cells in ${C}_1^k$ with that of the identified TFs ${E}_{C_1^k}^{i_{TF}}$ using nonnegative least squares regression, i.e., ${\hat{\beta}}^i=\arg {\min}_{\beta^i}{\left\Vert {E}_{C_1^k}^{i_{TF}}-{E}_{C_1^k}^i{\beta}^i\right\Vert}_2^2,s.t.{\beta}^i\ge 0$. Regulatory relationships were inferred if the regression coefficients ${\hat{\beta}}^i$ of the TFs were greater than zero.

### Validation of the inferred regulatory relationships

To validate the inferred regulatory relationships in A549 dataset, we collected all TFs that regulate the marker genes (ABHD12, BASP1, CDH16, CKB, NFKBIA, NR3C1, PER1, SCNN1A, and TXNRD1) from the hTFtarget database (http://bioinfo.life.hust.edu.cn/hTFtarget/), which curated a comprehensive TF-target regulation from various ChIP-seq datasets of human TFs from NCBI Sequence Read Archive (SRA) and ENCODE databases. We take ABHD12 as an example to compute the fold enrichment of the inferred regulatory relationships in this database. Among the total 374 collected TFs in chromVAR, 92 TFs are found to regulate ABHD12 in hTFtraget. Among our identified 12 TFs of ABHD12 using chromVAR, 7 TFs are found to regulate ABHD12 in hTFtraget. Thus, the fold enrichment of our predicted regulations of ABHD12 is calculated by (7/12)/(92/374) = 2.37. A fold enrichment value greater than 1 indicates an over-representation of the inferred regulations in the database.

### Datasets and preprocessing

The kidney and A549 datasets were downloaded from GSM3271044 and GSM3271045, and GSM3271040 and GSM3271041, respectively. The preprocessed mESC dataset was obtained from a previous study [17]. The detailed description of these datasets and their preprocessing were shown in Additional file 1: Supplementary methods (*Details of datasets and preprocessing*).

### Feature selection

Two feature selection methods were used in this study. If the cell groups were known (e.g., at the time of data collection), the most informative genes were selected using a Wilcoxon rank-sum test with the same parameters as in the identification of factor-specific features. For example, for the scRNA-seq data in the A549 dataset, we identified the differentially expressed genes at different time points and used these genes as informative genes for the downstream analyses. For other datasets, the average expression of each gene and the Fano factor were first calculated. The Fano factor, defined as the variance divided by the mean, is a measure of dispersion. Next, the average expression of all genes was binned into 20 evenly sized groups, and the Fano factor within each bin was normalized using *z*-score. Then, genes with normalized Fano factors larger than 0.5 and average expressions larger than 0.01 were selected. Moreover, we also selected genes with larger Gini index values [66]. GiniClust R package was run with default parameters. Briefly, genes whose normalized Gini index is significantly above zero (*p* value < 0.0001) are labeled high Gini genes and selected for further analysis. For the kidney dataset, we selected the informative genes using the second method and loci that were within 50 kb of the TSS of these informative genes.

### Method comparisons on three datasets

We compare the performance of scAI with three other methods, including MOFA [17], Seurat (version 3) [22], and LIGER [23]. MOFA takes normalized scRNA and scATAC-seq data as inputs, then infers latent factors using a generalized PCA and assesses the proportion of variance explained by each factor in each type of data. Seurat derives a “gene activity matrix” from the peak matrix of the scATAC-seq data by simply summing all counts within the gene body + 2 kb upstream, representing a synthetic scRNA-seq dataset to leverage for integration. Seurat then co-embeds the scRNA-seq and scATAC-seq cells in the same low-dimensional space by identifying “anchors” between the ATAC-seq and RNA-seq datasets. Since LIGER does not provide specific functions for integrating scRNA-seq and scATAC-seq or DNA methylation data, we used scRNA-seq data and the inferred “gene activity matrix” from Seurat as inputs for integrative analysis. The detailed description of how these comparisons were performed is available in Additional file 1: Supplementary methods (*Details of method comparisons on three datasets*).

Based on the first two dimensions of t-SNE or UMAP, we quantify the alignment score of the scRNA-seq and scATAC-seq cells using entropy of batch mixing, and assess the separation of the cell groups using silhouette coefficient. These two evaluation metrics were defined in [55]. The detailed description is available in Additional file 1: Supplementary methods (*Details of method comparisons on three datasets*).

## Supplementary information

**Additional file 1.** Supplementary Methods.

**Additional file 2.** Supplementary Figures and Tables.

**Additional file 3.** Review history.

## Data Availability

scAI is implemented as both MATLAB and R packages, which are freely available under the GPL-3 license. Source codes as well as the workflows of simulation and real datasets have been deposited at the GitHub repository (MATLAB package: https://github.com/amsszlh/scAI [67] and R package: https://github.com/sqjin/scAI) [68]. The datasets analyzed in this study are available from the Gene Expression Omnibus (GEO) repository under the following accession numbers: GSM3271044 and GSM3271045 [6], GSM3271040 and GSM3271041 [6], and GSE74535 [3].
