# Supplementary material for: scAI: an unsupervised approach for the integrative analysis of parallel single-cell transcriptomic and epigenomic profiles
Source: Genome Biol. 2020 Feb 3;21:25. doi: 10.1186/s13059-020-1932-8 (PMC6996200; doi:10.1186/s13059-020-1932-8)
Supplement: Supplementary file 1 — Additional file 1. Supplementary Methods. [file 13059_2020_1932_MOESM1_ESM.pdf]

Additional file 1: Supplementary Methods for "scAI: an unsupervised method for the integrative analysis of parallel single-cell transcriptomic and epigenomic profiles"

This file includes the following subsections:

- Details of scAI
- Rank selection
- Robustness analysis
- Simulation datasets
- Details of datasets and preprocessing
- Details of clustering analysis on kidney dataset
- Details of method comparisons on three datasets
- Evaluation metrics
- Comparison of cell-cell similarity matrix
- Details of method comparisons on single omics data
- References

Details of scAI

In this section, we presented the multiplicative update method for solving the following optimization problem of scAI model

$$\min_{W_1,W_2,H,Z\geq 0} F = \alpha\left\|X_1 - W_1H\right\|_F^2 + \left\|X_2(Z\circ R) - W_2H\right\|_F^2 + \lambda\left\|Z - H^TH\right\|_F^2 + \gamma\sum_j\left\|H_{.j}\right\|_1^2, \quad (1)$$

where $\circ$ represents dot multiplication and α, λ, γ are the parameters that balance each part of the objective function. *R* is a binary matrix generated by a binomial distribution. $H_{.j}$ is the *j*-th column of *H*. The key ideas of proposing such an objective function are as follows: i) Cells are projected into a common space *H* from both transcriptomic (X_1) and epigenomic (X_2) data. ii) However, epigenomic (e.g., scATAC-seq) data is very sparse and nearly binary, thus aggregation of data in similar cells is needed. These similar cells are analyzed by simultaneously learning a cell-cell similarity matrix from both transcriptomic and epigenomic data using a low-rank matrix factorization model. iii) The cell-cell similarity matrix *Z* can be approximated by H^TH.

Based on linear algebra, the objective function *F* can be reformulated as

$$\begin{aligned} F = {} & \alpha Tr(X_1^TX_1 - H^TW_1^TX_1 - X_1^TW_1H + H^TW_1^TW_1H) + Tr(ZR^TX_2^TX_2ZR - H^TW_2^TX_2ZR \\ & - ZR^TX_2^TW_2H + H^TW_2^TW_2H) + \lambda Tr(Z^TZ - Z^TH^TH - H^THZ + H^THH^TH) \\ & + \gamma e^THH^Te \end{aligned} \quad (2)$$

where $ZR = Z\circ R$, *e* is a $K*1$ vector with each element being 1 and *K* is the rank of *H*.

The Lagrange function of Eq. (2) is

$$L = F + trace(\Phi_1 W_1) + trace(\Phi_2 W_2) + trace(\Psi H) + trace(\Theta Z) \tag{3}$$

where $\Phi_1, \Phi_2, \Psi, \Theta$ is the Lagrange multiplier for constraints $W_1, W_2, H, Z \geq 0$. The partial derivatives of *L* with respect to *W1*, *W2*, *H* and *Z* are

$$\frac{\partial L}{\partial W_1} = -2\alpha X_1 H^T + 2\alpha W_1 HH^T + \Phi_1 \tag{4}$$

$$\frac{\partial L}{\partial W_2} = -2X_2 ZRH^T + 2W_2 HH^T + \Phi_2 \tag{5}$$

$$\begin{aligned}\frac{\partial L}{\partial H} = & -2\alpha W_1^T X_1 + 2\alpha W_1^T W_1 H - 2W_2^T X_2 ZR + 2W_2^T W_2 H - 2\lambda H(Z + Z^T) + 4\lambda HH^T H \\ & + 2\gamma ee^T H + \Psi\end{aligned} \tag{6}$$

$$\frac{\partial L}{\partial Z} = 2(X_2^T X_2 ZR) \circ R - 2(X_2^T W_2 H) \circ R + 2\lambda Z - 2\lambda H^T H + \Theta \tag{7}$$

Based on the KKT conditions: $\frac{\partial L}{\partial W_1}, \frac{\partial L}{\partial W_2}, \frac{\partial L}{\partial H}, \frac{\partial L}{\partial Z} = 0$, $W_{ij}^1 \Phi_{ij}^1 = 0, W_{ij}^2 \Phi_{ij}^2 = 0, H_{ij} \Psi_{ij} = 0$ and $Z_{ij} \Theta_{ij} = 0$, we obtain the following equations for $W_{ij}^1, W_{ij}^2, H_{ij}$ and Θ_{ij},

$$(2X_1 H^T - 2W_1 HH^T)_{ij} W_{ij}^1 = 0 \tag{8}$$

$$(2X_2 ZRH^T - 2W_2 HH^T)_{ij} W_{ij}^2 = 0 \tag{9}$$

$$\begin{aligned}&(2\alpha W_1^T X_1 - 2\alpha W_1^T W_1 H + 2W_2^T X_2 ZR - 2W_2^T W_2 H + 2\lambda H(Z + Z^T) \\ &-4\lambda HH^T H - 2\gamma ee^T H)_{ij} H_{ij} = 0\end{aligned} \tag{10}$$

$$(-2(X_2^T X_2 ZR) \circ R + 2(X_2^T W_2 H) \circ R - 2\lambda Z + 2\lambda H^T H)_{ij} Z_{ij} = 0 \tag{11}$$

Thus, we obtain the following updating rules:

$$W_{ij}^1 \leftarrow W_{ij}^1 \frac{(X_1 H^T)_{ij}}{(W_1 HH^T)_{ij}} \tag{12}$$

$$W_{ij}^2 \leftarrow W_{ij}^2 \frac{(X_2 ZRH^T)_{ij}}{(W_2 HH^T)_{ij}} \tag{13}$$

$$H_{ij} \leftarrow H_{ij} \frac{(\alpha W_1^T X_1 + W_2^T X_2 ZR + \lambda H(Z + Z^T))_{ij}}{((\alpha W_1^T W_1 + W_2^T W_2 + 2\lambda HH^T + \gamma ee^T)H)_{ij}} \tag{14}$$

$$Z_{ij} \leftarrow Z_{ij} \frac{((X_2^T W_2 H) \circ R + \lambda H^T H)_{ij}}{((X_2^T X_2 ZR) \circ R + \lambda Z)_{ij}} \tag{15}$$

---

scAI algorithm

Step 1: Initialize *W1*, *W2*, *H*, *Z* using a 0-1 uniform distribution and generate a binary matrix *R* using a Bernoulli distribution. Set the iteration *t* to 0.

Step 2: Fix *W2*, *H* and *Z*, and then update *W1* by Eq. (12).

Step 3: Fix *W1*, *H* and *Z*, and then update *W2* by Eq. (13).

Step 4: Fix W_1, W_2 and Z, and then update H by Eq. (14).

Step 5: Fix W_1, W_2 and H, and then update Z by Eq. (15).

Step 6: Set $t \leftarrow t+1$ and repeat Step 2-5 until satisfying the stop criterion.

Here, there are two stop criterions for this algorithm. On the one hand, the stop criterion is whether the objective function tends to be invariant, that is $\frac{F(t)-F(t+1)}{F(t)} \leq 10^{-6}$, where $F(t)$ is the objective function in Eq. (2) of the t-th step. On the other hand, the algorithm will be terminated if the number of iterations t is larger than 500 steps. The computational complexity of scAI algorithm is $O(T(n^3+Kn^2+pnK+qnK+pK^2+qK^2))$, where T is the number of steps, n is the number of cells, p is the number of genes, q is the number of loci, K is the rank. The convergence of scAI algorithm on all datasets is shown in Additional file 2 Figure S16.

Rank selection

The rank K is determined by a stability-based method proposed in a previous study [1]. Briefly, for each rank K drawn from a given range, we constructed a consensus matrix A based on a set of cell loading matrices H that were obtained from 10 runs of scAI with different seeds in parallel. Specifically, cell j is assigned to the k-th cluster if H^{kj} is the maximum value of $H_{.j}$, where H^{kj} is the k-th row and j-th column of H and $H_{.j}$ is the j-th column of H. The connectivity matrix B is defined by B_{ij} and equals 1 if cells i and j are assigned to the same cluster for each run. Then the consensus matrix A is obtained by averaging the entries of B for all runs. Therefore, each entry A_{ij} for A varies from 0 to 1 and represents the probability of cells i and j being in the same cluster. Finally, we selected K based on the Cophenetic correlation coefficient (*Coph*), which is measured by the Pearson correlation between I-A and the distance between samples reordered by linkage after applying hierarchical clustering on A with average linkage. For a range of ranks, a suitable rank K is the one at which the magnitude of *Coph* begins to fall [1]. Additional file 2 Figure S17 and Figure S18 showed the evolution of consensus matrices and *Coph* with different ranks on the eight simulation datasets and three real datasets.

Robustness analysis

For the used parameters (α, λ, γ and s) in all datasets, we performed robustness analysis of the accuracy of reconstructed cell loading matrix H on the simulation data based on Area Under receiver operating characteristic Curve (AUC) and Area Under Precision-Recall curve (AUPR) as the true labels are available for simulation data. For the three real datasets, since there are no true labels, we evaluated the change of *Coph* values. Robustness analysis showed that the overall performance of scAI is relatively robust to choices of parameter values within certain ranges (Additional file 2 Figure S19). The parameter γ controls the sparseness of H. The block structures of the inferred cell-cell similarity matrix Z will become more obvious if the sparsity of H increases to some extent. From this point, scAI will do better in terms of

AUC/AUPR to some extent when increasing parameter \gamma. However, if H is too sparse, it might lead to decreased AUC/AUPR values, such as in the simulation datasets 1 and 4.

Simulation datasets

Simulation dataset 1. To show the influence of sparse level on the performance of scAI, dataset 1 was simulated by increasing sparse levels. Firstly, we set the true rank *K* to 3, and the ground truth basis matrices W_1 and W_2 were constructed as follows,

$$W_1(i,j)=\begin{cases}1,\ 1\le i\le 100\ \ j=1;\ 151\le i\le 300\ \ j=2;\ 501\le i\le 800\ \ j=3,\\ \qquad\qquad\qquad 0,\ \text{otherwise.}\end{cases}$$

$$W_2(i,j)=\begin{cases}1,\ 1\le i\le 500\ \ j=1;\ 1001\le i\le 1500\ \ j=2;\ 3001\le i\le 3800\ \ j=3,\\ \qquad\qquad\qquad 0,\ \text{otherwise}\end{cases}$$

Gaussian noises were added to W_1 and W_2 as follows, $W_1=W_1+\rho E$, $W_2=W_2+\rho E,$ where E is the Gaussian noise and ρ equals 0.5. The negative values of W_1 and W_2 were set to zero. Meanwhile, the ground truth cell loading matrix H that was used to obtain clustering information was constructed as follows,

$$H(i,j)=\begin{cases}1,\ i=1\ \ 1\le j\le 70;\ i=2\ \ 71\le j\le 130;\ i=3\ \ 131\le j\le 200\\ \qquad\qquad\qquad 0,\ \text{otherwise.}\end{cases}$$

Then the ground truth data matrices were $X_1=W_1H,\ X_2=W_2H.$ Dropouts were generated by a Bernoulli distribution on X_1 and X_2 with the probabilities p_{1i}, p_{2j}, which were defined as $p_{1i}=e^{-\lambda_1 x_i^2}$, $p_{2j}=e^{-\lambda_2 y_j^2}$, where x_i is the mean expression level of the *i*-th cluster of X_1, y_j is the mean expression level of the *j*-th cluster of X_2. Next, Gaussian noises were added to X_1 and X_2 as $X_1=X_1+\rho_1 E,\ X_2=X_2+\rho_2 E$. In dataset 1, we set ρ_1 to be 2 and ρ_2 to be 1. In order to make matrices X_1, X_2 be nonnegative, we set negative values to be 0. In addition, to simulate a binary matrix X_2, we set the values to 1 if the values in X_2 were larger than 0.7, otherwise to 0. To obtain datasets with various sparse levels, we varied λ_1 from 0.25 to 0.05 with an increment being -0.05 and λ_2 from 0.125 to 0.025 with an increment being -0.025. Finally, we obtained five data matrix pairs (data matrix X_1 and X_2) with increasing sparse levels, which varied from 92.5% to 98.2% for X_1 and from 96.9% to 99.3% for X_2.

Simulation dataset 2. To evaluate the performance of scAI with respect to the noise levels, we simulated dataset 2 using aforementioned procedures, in which $\lambda_1=0.05,\ \lambda_2=0.025,\ \rho_1$ varied from 3 to 5 with an increment 0.5, and ρ_2 varied from 0.2 to 1 with an increment 0.2.

Simulation datasets 3 and 4. To evaluate the performance of scAI in identifying cell clusters with either transcriptomic *or* epigenetic distinctions, we simulated dataset 3 and 4 by adopting the simulation strategy

in the previous study [2]. In more details, we suppose there were 2000 genes and 5000 loci across 500 cells. The ground truth of basis matrices W_1 and W_2 were generated with *coph = 0*.

$$W_{ik}^1 = \begin{cases} 1, & 1+x_k(200) \le i \le 200 + x_k(200) \\ 0, & \text{otherwise.} \end{cases}$$

$$W_{ik}^2 = \begin{cases} 1, & 1+x_k(500) \le i \le 500 + x_k(500) \\ 0, & \text{otherwise.} \end{cases}$$

where $x_j(n) = (j-1)(n - coph)$. Suppose that the rank of W_1 was K_1 and the rank of W_2 was K_2. **In simulation dataset 3**, we varied K_1 from 3 to 7 and set K_2 to be 3; that is, *some clusters that were defined from gene expression do not reflect epigenetic distinctions*. On the contrary, **in simulation dataset 4**, we set K_1 to be 3 and varied K_2 from 3 to 7; that is, *some clusters that were defined from chromatin accessibility do not reflect transcriptomic distinctions*. Similar to dataset 1 and dataset 2, Gaussian noises were added to W_1 and W_2 with ρ equaling 0.5, $W_1 = W_1 + \rho E$, $W_2 = W_2 + \rho E.$ We also set the negative values of W_1 and W_2 to be 0. Let K be the maximum value of K_1 and K_2, we expanded W_1 and W_2 to form K columns basis matrices by adding some columns with zeros. The ground truth H was simulated with *coph = 0* as follows,

$$H_{kj} = \begin{cases} 1, & 1+x_k(c) \le j \le c + x_k(c), k \le K-1 \text{ or } 1+\mathrm{x}_K(c) \le \mathrm{i} \le \mathrm{n,k=K} \\ 0, & otherwise. \end{cases}$$

Then the ground truth data matrices were $X_1 = W_1 H,\ X_2 = W_2 H.$ Next, similar to the procedures mentioned above, we added dropouts on both X_1 and X_2 with $\lambda_1 = 0.05, \lambda_2 = 0.025$ and added Gaussian noise with $\rho_1 = 2, \rho_2 = 1.$ In addition, if the values in X_2 with dropouts were greater than 0.7, we set the values to be 1, otherwise 0.

Simulation dataset 5. To recapitulate properties of real datasets, we generated simulation dataset 5 based on real datasets using a R package called MOSim [2]. Using this package, we respectively simulated bulk RNA-seq and DNase-seq with 2000 and 5000 features across 150 cells in three groups with 50 cells for each group. Then we used these bulk data to generate scRNA-seq and single cell DNase-seq data. First, we computed average expression level E_i^j for gene *i* in the *j*-th each group. Then we generated scRNA-seq without dropouts using a Possion distribution and obtained a dropout matrix using a Bernoulli distribution with the probability equaling $1/e^{\lambda_1 E_i^j / m(E_\cdot^j)}$, where $m(E_\cdot^j)$ represented median value of all gene expression level in the *j*-th group. Single cell DNase-seq data were constructed as mentioned above with dropout probability equaling $1/e^{\lambda_2 E_i^j / m(E_\cdot^j)}$. In this dataset, we varied both λ_1 and λ_2 from 0.05 to 0.01 with increment equaling -0.01.

Simulation dataset 6. Dataset 5 described three discrete cell states. In order to evaluate the performance of scAI in a continuous biological process, we simulated dataset 6. Similar to dataset 5, we simulated the bulk RNA-seq and DNase-seq across 250 cells at five time points with 50 cells for each time point. Next,

we generated single cell datasets based on these two data matrices using the same parameters of dataset 5.

Simulation dataset 7. We set the true rank of X_1 and X_2 to 2, and the ground truth basis matrices W_1 and W_2 were constructed as follows,

$$W_1(i,j)=\begin{cases}1, & 1\le i\le 200\ \ j=1;\ 401\le i\le 600\ j=2,\\ 0, & \text{otherwise.}\end{cases}$$

$$W_2(i,j)=\begin{cases}1, & 1\le i\le 2000\ \ j=1;\ 3001\le i\le 5000\ j=2,\\ 0, & \text{otherwise.}\end{cases}$$

In addition, the ground truth cell loading matrix H was constructed as follows,

$$H(i,j)=\begin{cases}1, & i=1\ 1\le j\le 100;\ i=2\ 101\le j\le 1100,\\ 0, & \text{otherwise.}\end{cases}$$

Other procedures were same as the **Simulation dataset 1**.

Simulation dataset 8. We set the true rank of X_1 and X_2 to 5 and 3, respectively, and the ground truth basis matrices W_1 and W_2 were similar to those of **Simulation dataset 3**. The ground truth H was simulated as follows,

$$H(i,j)=\begin{cases}1, & i=1\ 1\le j\le 50;\ i=2\ 51\le j\le 150,\ i=3\ 151\le j\le 350,\ i=4\ 351\le j\le 450,\ i=5\ 451\le j\le 550\\ 0, & \text{otherwise.}\end{cases}$$

The noise levels of this dataset were the same as the **Simulation dataset 1**.

All the simulated datasets were summarized in Additional file 2 Table S1.

Details of datasets and preprocessing

The kidney dataset downloaded from GEO (GSM3271044 and GSM3271045) included 11296 single cells from the mammalian kidney, in which the chromatin accessibility and gene expression in each single cell were simultaneously co-assayed using the sci-CAR protocol [3]. We merged duplicate genes with the maximum values of the corresponding transcripts and removed cells that were labeled as 'NA' in a previous study [3]. Then, cells with the number of expressed genes less than 500 and genes expressed in less than 10 cells were removed from the scRNA-seq data. Next, cells with the number of accessible loci less than 200 and loci opened in less than 10 cells were removed from the scATAC-seq data. Finally, 8837 cells were used for downstream analyses.

The A549 dataset downloaded from GEO (GSM3271040 and GSM3271041) included 3260 cells that were co-assayed using the sci-CAR protocol [3], with both scRNA-seq and scATAC-seq data available for the same cells. For the scRNA-seq data, genes expressed in less than 10 cells, and cells with expression counts less than 500 and more than 9100 were removed. For the scATAC-seq data, loci present in less than 5 cells and cells with less than 200 accessible loci were removed. Quality control resulted in a dataset with data from 2641 cells that was used for downstream analyses.

The mESC dataset was composed of 13 cells cultured in “2i” media and 64 serum-grown cells that were profiled in parallel by the single-cell methylation and transcriptome sequencing technique scM&T-seq [4]. The 2i-cultured cells were in a naïve pluripotency state, and the serum-grown cells were in a primed pluripotency state, which is poised to undergo cellular differentiation. Data were processed by an approach used in a previous study [5], which characterized DNA methylation in three different genomic contexts, including CpG islands, promoters and enhancers. In total, 15000 loci were identified in 77 cells after being matched with scRNA-seq data. The methylated levels of promoter, enhancer and CpG sites usually have negative correlations with gene expression levels. Since scAI aims at extracting similar patterns of genes and loci, the methylation data matrix was constructed in which the element was 0 if locus i was methylated in cell j or methylation information was missing; if locus i was unmethylated in cell j, the element was 1. Thus the unmethylated CpG sites were treated as 1, and the missing CpG sites were treated as 0 when applying scAI. In this way, the missing values of the input matrices (both single cell RNA and DNA methylation data) are 0.

The digital data matrices were normalized by a global method, in which the expression value of each gene (accessibility or methylation level of each locus) was divided by the total expression (accessibility or methylation level) in each cell and multiplied by a scale factor (10,000 by default). These values were then log-transformed with a pseudocount of 1. Normalized data were used for all the analyses.

Details of clustering analysis on kidney dataset

The *Coph* score and the consensus matrix were used to evaluate the stability of scAI in reconstructing the cell loading matrix *H* with different initial values for a given rank. In addition, as the entries of the consensus matrix were built based on the maximum value of columns of *H*, the rank with highest *Coph* score and clear block structures in the consensus matrix may not be the number of clusters on some datasets.

In the analysis of kidney dataset, based on the stability parameter *Coph* values, we set the rank to 20. By applying Leiden algorithm on the cell loading matrix H, we identified 17 clusters with either distinct gene expression or chromatin accessibility profiles with the default resolution parameter equaling 1. This result was mostly biologically driven and based on the differential gene expression and loci accessibility analysis. Similar to Scanpy [6] and Seurat [7], two of the most popular scRNA-seq data analysis tools, the number of subpopulations is determined by the resolution parameter in the community detection algorithm. How to determine the optimal number of clusters, in particular for large and complex datasets, still remains challenging.

Details of method comparisons on three datasets

Details of data analysis by MOFA. MOFA v1.1.1 was used for the integrative analysis of scRNA-seq and scATAC-seq data (https://github.com/bioFAM/MOFA). For A549 dataset, the same features (genes and loci) used in scAI were used as inputs of MOFA. MOFA takes normalized data as inputs, where both scRNA-

seq and scATAC-seq data were normalized using sctransform [8]. Then we run MOFA by setting the number of factors to be 3. Other parameters were used by default. The variance explained by each factor in one type of data was calculated using the function "calculateVarianceExplained". For the kidney dataset, we set the number of factors to be 20.

Similar to the observations on the simulation datasets (Fig. 2d in main text), MOFA cannot discover the biological variations in the scATAC-seq data as the variance explained by the learned factors in the scATAC-seq data was nearly zero (Additional file 2 Figure S8a-d). For the kidney dataset, although the variance explained by the inferred latent factor 6 (LF6) in the scATAC-seq data is relatively large, there is a strong correlation between the cell loadings in LF6 and the total chromatin accessibility of each cell (Additional file 2 Figure S8e), indicating that the captured variance in LF6 is not biologically relevant.

Details of data analysis by Seurat. Seurat v3.0.2 [7] was used for the integrative analysis of scRNA-seq and scATAC-seq data based on the vignette provided in https://satijalab.org/seurat/v3.0/atacseq_integration_vignette.html. Briefly, we first analyzed the scRNA-seq data using the standard workflow in Seurat (https://satijalab.org/seurat/v3.0/pbmc3k_tutorial.html). To preprocess scATAC-seq data, the "gene activity matrix" was estimated using the function "CreateGeneActivityMatrix" with default parameters. Then the Seurat object was created using "CreateSeuratObject". Anchors between the scATAC-seq dataset and the scRNA-seq dataset were identified using "FindTransferAnchors" function. Finally, the cells from scRNA-seq and scATAC-seq data were co-embeded in the same low-dimensional space using RunTSNE and RunUMAP function after imputing RNA levels in the ATAC-seq data ("TransferData" function) and merging the "RNA" and the "ATAC" Seurat objects. For A549 dataset, 12 principal components (PCs) was used for RunTSNE, which was determined using "DimHeatmap" and "ElbowPlot" functions. The highly variable genes for analyzing scRNA-seq data were the same as used in scAI. For kidney dataset, 16 PCs was used for RunUMAP. The default parameters of "FindVariableFeatures" were used for feature selection.

Details of data analysis by LIGER. LIGER v0.4.1 was used for the integrative analysis of scRNA-seq and scATAC-seq data [9] (https://github.com/MacoskoLab/liger). As LIGER does not provide specific functions for the integrative analysis of scRNA-seq and scATAC-seq data, we run LIGER workflow by taking the scRNA-seq data and the inferred "gene activity matrix" from Seurat v3 as inputs. For A549 dataset, we also used the same genes used in scAI as highly variable genes. 3 factors were used in "optimizeALS" function. For kidney dataset, 20 factors were used in "optimizeALS" function.

Evaluation metrics

We calculated the entropy of batch mixing and silhouette coefficient using the approach described in a previous study [10] and related R codes in https://github.com/MarioniLab/MNN2017.

*Entropy of batch mixing***.** The total entropy of batch mixing on the first two low-dimensions is calculated by the sum of regional mixing entropies at the location of 100 randomly chosen cells. The regional entropy of mixing for *c* different batches is defined as

$$E = \sum_{i=1}^{c} x_i \log(x_i)$$

where x_i is the proportion of cells from batch *i* in a given region, such that $\sum_{i=1}^{c} x_i = 1$. The regional proportion of cells from each batch was defined from the set of 100 nearest neighbours for each randomly chosen cell. 100 iterations with different randomly chosen cells are repeated to generate boxplots of the total entropy.

Silhouette coefficient. The silhouette coefficient for cell *i* is defined as:

$$s(i) = \begin{cases} 1 - \dfrac{a(i)}{b(i)} & \text{if } a(i) < b(i) \\ 0 & \text{if } a(i) = b(i) \\ \dfrac{b(i)}{a(i)} - 1 & \text{if } a(i) > b(i) \end{cases}$$

where *a(i)* be the average distance of cell *i* to all other cells within the same cell group as *i*, and *b(i)* be the average distance of cell *i* to all cells assigned to the neighbouring group, i.e., the group with the lowest average distance to the group of *i*. We calculated the silhouette coefficients using distance matrices computed from the coordinates of each cell on the first two dimensions of tSNE or UMAP. A larger silhouette coefficient of one cell implies that the cell is close to other cells in the same group yet distant from cells in other cell groups.

Comparison of cell-cell similarity matrix

In scAI model, *Z* is the cell-cell similarity matrix, which is first randomly generated in the algorithm, and then updated during the iterative learning process. Supplementary Figure S11 in Additional file 2 presents the evolution of the third term in Eq.1 (i.e., $\|Z - HH^T\|_F$), which quantifies the difference between the similarity matrix of projecting cells and the matrix *Z*. For both simulation and real datasets, the differences converge to zeros over the iteration, as expected.

The inferred cell-cell similarity matrix Z by scAI captures the data structures from both transcriptomic and epigenomic profiles. To show its difference with the similarity matrix that is computed using only scRNA-seq or aggregated single-cell epigenomic data, we computed the Pearson's correlations between cells using scRNA-seq or aggregated single-cell epigenomic data. As shown in Figure S12 (Additional file 2), if there are subpopulations with distinct transcriptomic profiles but similar epigenomic profiles, then the similarity matrix of cells calculated using aggregated epigenomic data should be very different from the cell-cell similarity matrix Z of projecting cells. One such example is the simulation dataset 3. If there are subpopulations with distinct epigenomic profiles but similar transcriptomic profiles, then the similarity matrix

of cells before integrating epigenomic profile should be very different from the one after integrating epigenomic profile. One such example is the simulation dataset 4. However, if all the subpopulations exhibit differences in both transcriptomic and epigenomic profiles, the two similarity matrices should be similar.

Details of method comparisons on single omics data

Details of data analysis by Seurat. Seurat v3.0.2 [7] was used for the analysis of scRNA-seq data based on the vignette provided in https://satijalab.org/seurat/v3.1/pbmc3k_tutorial.html. Briefly, to identify cell clusters, principle component analysis (PCA) was first performed on the top 2000 highly variable genes, which were determined by FindVariableFeatures function. The top 30 PCs were used for clustering with the Louvain modularity-based community detection algorithm to generate cell clusters (FindNeighbors and FindClusters functions). The resolution parameter was determined based on the number of clusters in each dataset. For all the simulation datasets and A549 dataset, the same set of genes used in scAI was used as inputs.

Details of data analysis by SC3. SC3 v1.10.1 [11] was used for the analysis of scRNA-seq data based on the vignette provided in https://github.com/hemberg-lab/SC3/blob/master/vignettes/SC3.Rmd. Default parameters were used in all the analysis. The number of clusters used in the 'sc3' function was given in each dataset. For all the simulation datasets and A549 dataset, the same set of genes used in scAI was used as inputs.

Details of data analysis by Signac. Signac is an extension of Seurat for the analysis of scATAC-seq data. Signac v0.1.6 was used for the analysis of scATAC-seq data based on the vignette provided in https://satijalab.org/signac/articles/pbmc_vignette.html. Default parameters were used in all the analysis. The resolution parameter was determined based on the number of clusters in each dataset.

Details of data analysis by scABC. scABC v0.99.0 [12] was used for the analysis of scATAC-seq data based on the vignette provided in https://github.com/SUwonglab/scABC/blob/master/vignettes/ClusteringWithCountsMatrix.Rmd. Default parameters were used in all the analysis. The number of clusters used in the 'computeLandmarks' function was given in each dataset.

Reference

1. Brunet JP, Tamayo P, Golub TR, Mesirov JP. Metagenes and molecular pattern discovery using matrix factorization. P Natl Acad Sci USA 2004;101:4164-9.
2. Martínez-Mira C, Conesa A, Tarazona S. MOSim: Multi-Omics Simulation in R. bioRxiv 2018;421834.
3. Cao J, Cusanovich D, Ramani V, Aghamirzaie D, Pliner H, Hill AJ, Daza R, McFaline-Figueroa J, Packer J, Christiansen L, et al. Joint profiling of chromatin accessibility and gene expression in thousands of single cells. Science 2018;361:1380-5.
4. Angermueller C, Clark SJ, Lee HJ, Macaulay IC, Teng MJ, Hu TX, Krueger F, Smallwood S, Ponting CP, Voet T, et al. Parallel single-cell sequencing links transcriptional and epigenetic heterogeneity. Nat Methods 2016;13:229-32.

5. Argelaguet R, Velten B, Arnol D, Dietrich S, Zenz T, Marioni JC, Buettner F, Huber W, Stegle O. Multi-Omics Factor Analysis-a framework for unsupervised integration of multi-omics data sets. Mol Syst Biol 2018;14:e8124.
6. Wolf FA, Angerer P, Theis FJ. SCANPY: large-scale single-cell gene expression data analysis. Genome Biol 2018;19:15.
7. Stuart T, Butler A, Hoffman P, Hafemeister C, Papalexi E, Mauck WM, 3rd, Hao Y, Stoeckius M, Smibert P, Satija R. Comprehensive integration of single-cell data. Cell 2019;177:1888-902.
8. Hafemeister C, Satija R. Normalization and variance stabilization of single-cell RNA-seq data using regularized negative binomial regression. bioRxiv 2019:576827.
9. Welch JD, Kozareva V, Ferreira A, Vanderburg C, Martin C, Macosko EZ. Single-cell multi-omic integration compares and contrasts features of brain cell identity. Cell 2019;177:1873-87.
10. Haghverdi L, Lun ATL, Morgan MD, Marioni JC. Batch effects in single-cell RNA-sequencing data are corrected by matching mutual nearest neighbors. Nat Biotechnol 2018;36:421-7.
11. Kiselev VY, Kirschner K, Schaub MT, Andrews T, Yiu A, Chandra T, Natarajan KN, Reik W, Barahona M, Green AR, Hemberg M. SC3: consensus clustering of single-cell RNA-seq data. Nat Methods 2017;14:483-6.
12. Zamanighomi M, Lin ZX, Daley T, Chen X, Duren Z, Schep A, Greenleaf WJ, Wong WH. Unsupervised clustering and epigenetic classification of single cells. Nat Commun 2018;9:2410.
