# Supplementary material for: scAI: an unsupervised approach for the integrative analysis of parallel single-cell transcriptomic and epigenomic profiles
Source: Genome Biol. 2020 Feb 3;21:25. doi: 10.1186/s13059-020-1932-8 (PMC6996200; doi:10.1186/s13059-020-1932-8)
Supplement: Supplementary file 2 — Additional file 2. Supplementary Figures and Tables. [file 13059_2020_1932_MOESM2_ESM.pdf]

Additional file 2: Supplementary Figures and Tables for "scAI: an unsupervised method for the integrative analysis of parallel single-cell transcriptomic and epigenomic profiles"

This file includes Supplementary Figures S1-S19 and Tables S1-S2.

Supplementary Figures

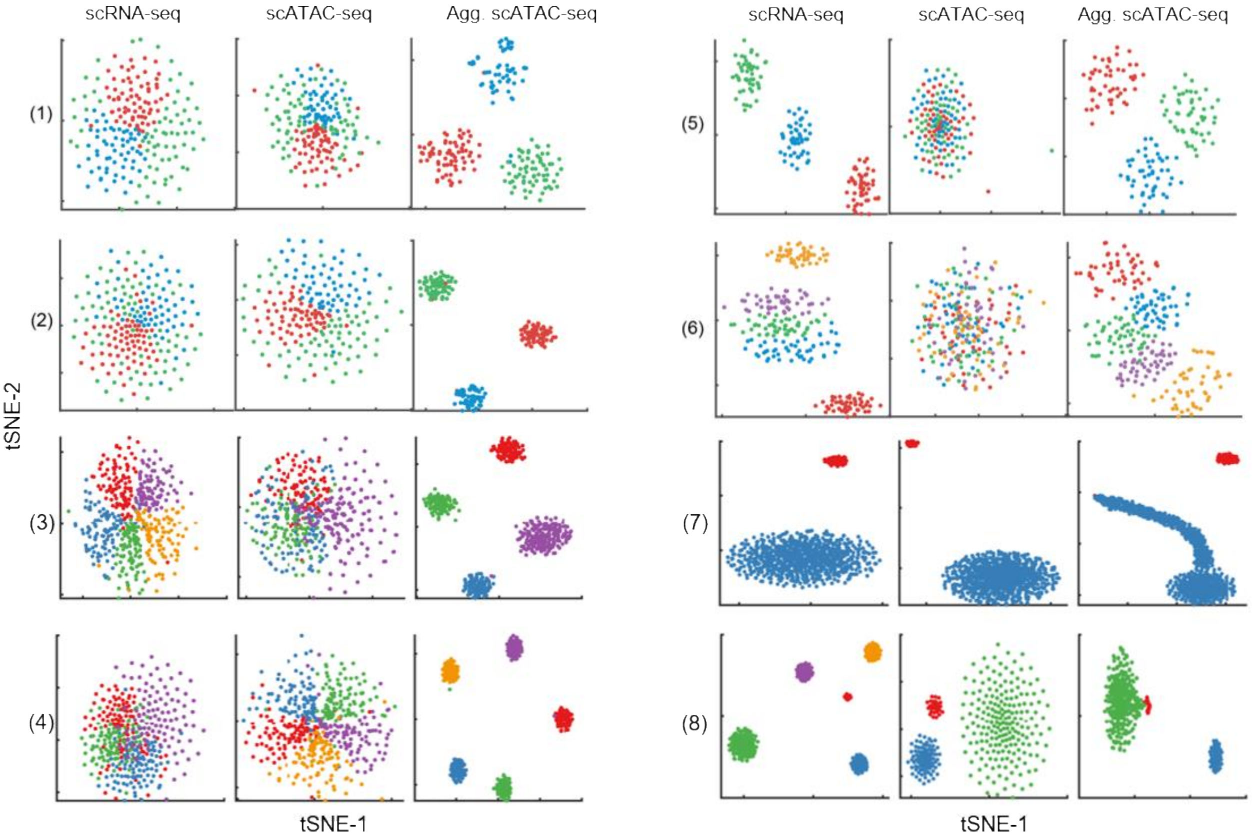

Figure S1. 2D visualization of cells by applying tSNE to scRNA-seq, scATAC-seq, and aggregated scATAC-seq data obtained from scAI. Each number shows one example of each scenario from the simulated datasets. Cells are colored based on their true labels.

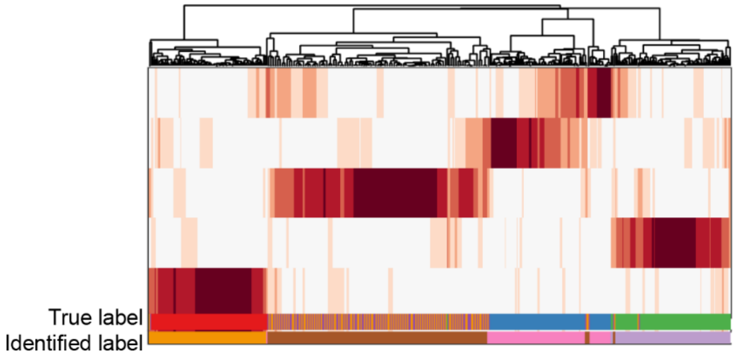

Figure S2. Clustering analysis of scRNA-seq data from simulation dataset 4. Heatmap of the cell loading matrix *H* obtained from the typical nonnegative matrix factorization of scRNA-seq data from simulation dataset 4. Cells are ordered based on hierarchical clustering. Bottom bars: cells are colored by the simulated true labels (five clusters) and the identified cell clusters by performing Leiden algorithm on *H*. This result shows that only four clusters exhibit distinct gene expression patterns.

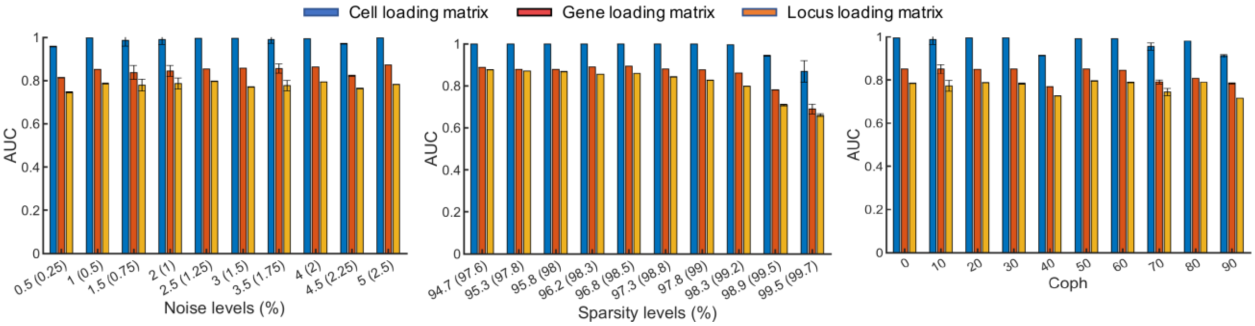

Figure S3. The performance of scAI with different noise, sparsity and discriminative levels on simulation dataset 8. Here we evaluated the performance of scAI in terms of the accuracy of reconstructed cell loading (blue color), gene loading (orange color) and locus loading (yellow color) matrices, which was quantified by AUC. We made the initial data less discriminative among clusters by increasing the parameter value *Coph*.

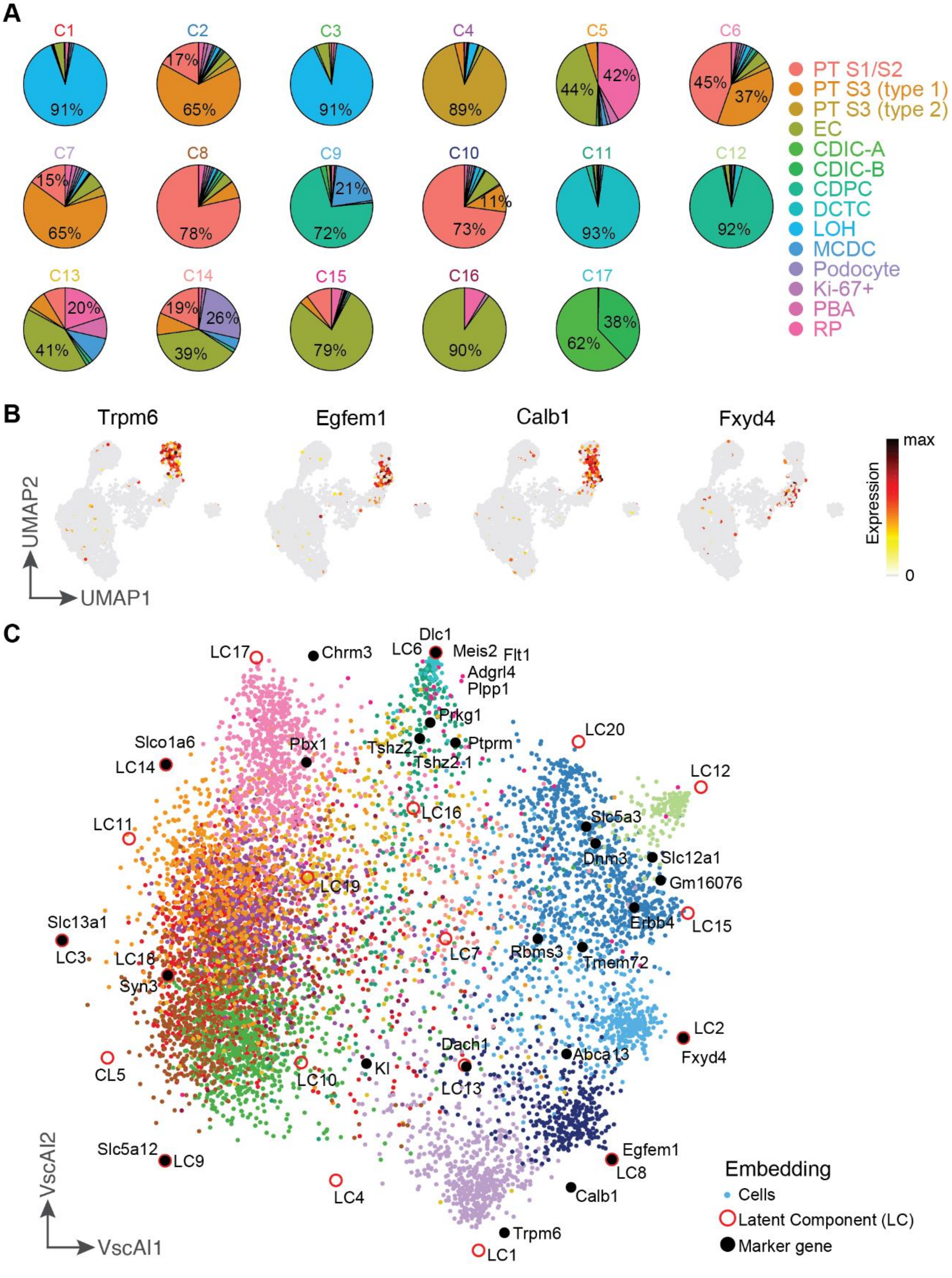

Figure S4. Application of scAI to the transcription and chromatin accessibility data from mammalian kidney. (a) Distribution of the assigned cell labels from the original study [1] in each identified cluster with scAI (each pie chart). The percentage of major enriched cell labels in each cell cluster is indicated. **(b)** VscAI visualization of cells, marker genes and inferred factors by embedding them into a two-dimensional space. Cells are colored based on the identified clusters with scAI. **(c)** Feature plots showing the expression

distribution for marker genes of DCTC cell type (captured by factor 1) and two subpopulations of CDPC cell type (captured by factors 8 and 2 respectively). Expression levels for each cell are color-coded and overlaid onto the UMAP plot. Cells with the highest expression level are colored dark red.

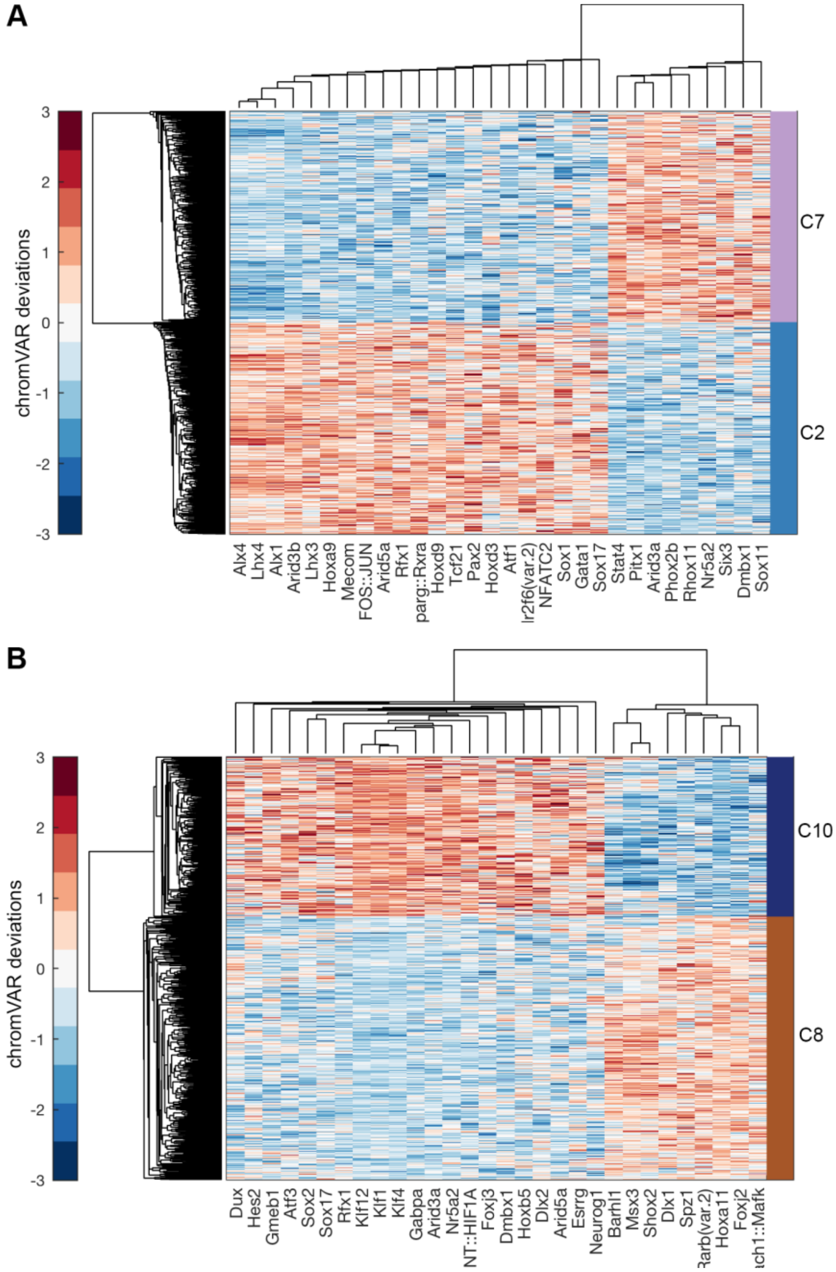

Figure S5. Validation of the differential accessibility patterns identified by scAI using chromVAR on Kidney dataset. Hierarchical clustering of chromVAR deviations for the top 30 most variable TFs (columns) (based on the variability scores) and cells (rows), which were calculated using the differential accessible loci of C2 and C7 (a), and C8 and C10 (b). Hierarchical clustering analysis of deviations divides motifs into two groups, each specific to just one cluster.

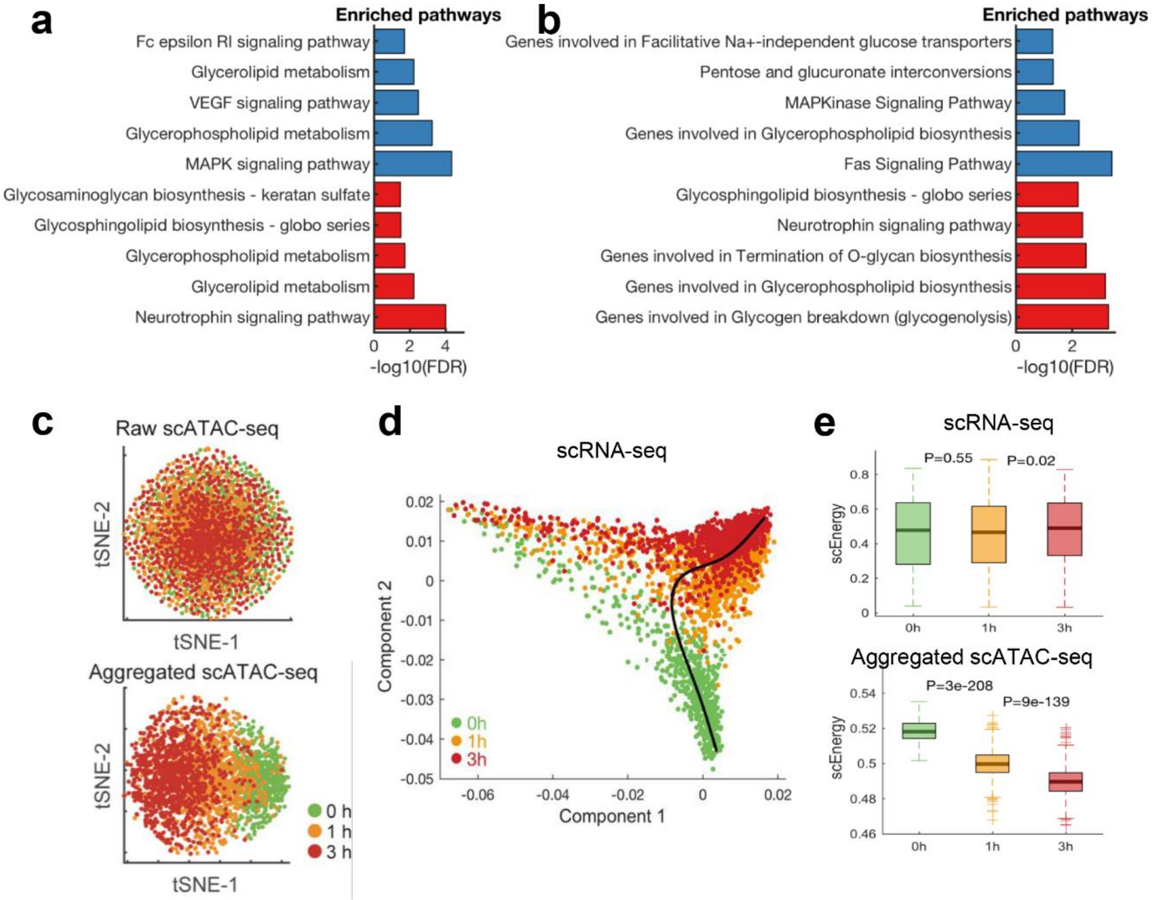

Figure S6. Application of scAI to the transcription and chromatin accessibility data in dexamethasone-treated A549 cells. (a) Top enriched biological pathways of the factor specific genes using MSigDB. Red color represents factor 1, while blue color represents factor 2. **(b)** Top enriched biological pathways of the nearby genes of the factor specific loci using GREAT. **(c)** t-SNE plot of scATAC-seq data (top) and aggregated scATAC-seq obtained with scAI (bottom). Cells are colored by the experimental time. **(d)** The inferred pseudotemporal trajectories of cells by applying scEpath to scRNA-seq data. Cells were projected onto the low-dimensional space generated by diffusion maps. Black line is the fitted principal curve. **(e)** Comparison of scEnergy of different cell groups calculated from scRNA-seq data (top) and aggregated scATAC-seq data (bottom). P-values are from rank sum test.

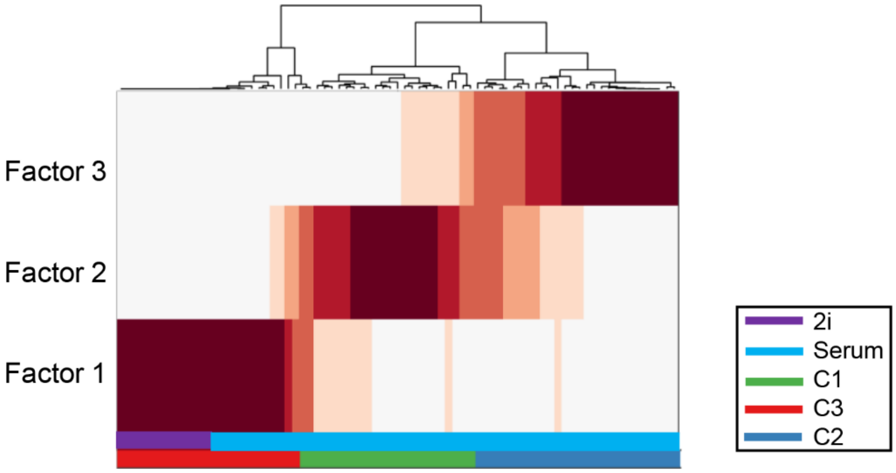

Figure S7. Correspondence between the identified cell clusters and factors in mESC dataset. Heatmap of the cell loading matrix H obtained from scAI on mESC dataset. Cells are ordered based on hierarchical clustering analysis. Bottom bars: The color of cells is labeled by cultured conditions and cell clusters identified by scAI.

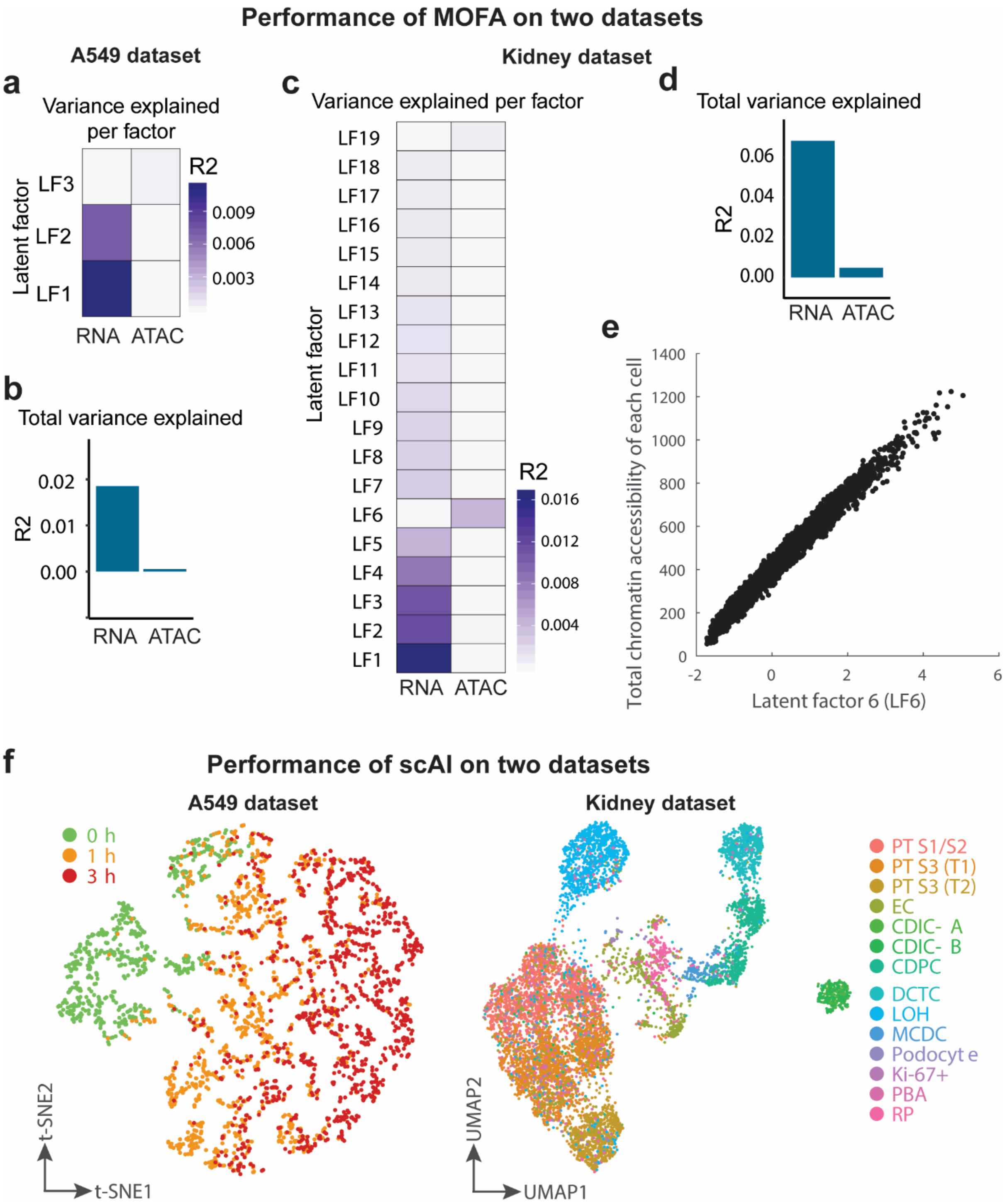

Figure S8. Performance of MOFA and scAI on the two datasets. (a-b) Performance of MOFA on the A549 dataset. The variance explained by each inferred factor and the total variance explained in each type of data are shown. The variance explained in the scATAC-seq data is nearly zero. **(c-e)** Performance of MOFA on the kidney dataset. Although the variance explained by the inferred latent factor 6 (LF6) in the scATAC-seq data is relatively large, there is a strong correlation between the cell loadings in LF6 and the total chromatin accessibility of each cell, indicating that the captured variance in LF6 is not biologically relevant. **(f)** Performance of scAI on the A549 dataset and kidney dataset. **Left:** A549 cells were projected into the first two t-SNE dimensions based on the inferred cell loading matrix *H* from scAI. Cells are colored

by collected time points. **Right:** Kidney cells were projected into the first two UMAP dimensions based on the inferred cell loading matrix *H* from scAI. Cells are colored by published cell labels from the original study [1].

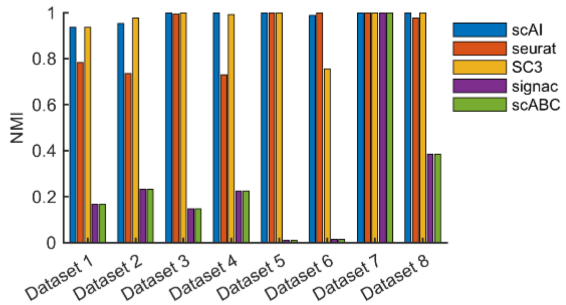

Figure S9. Comparison of scAI with other methods in identifying cell clusters using single omics data on simulation datasets.

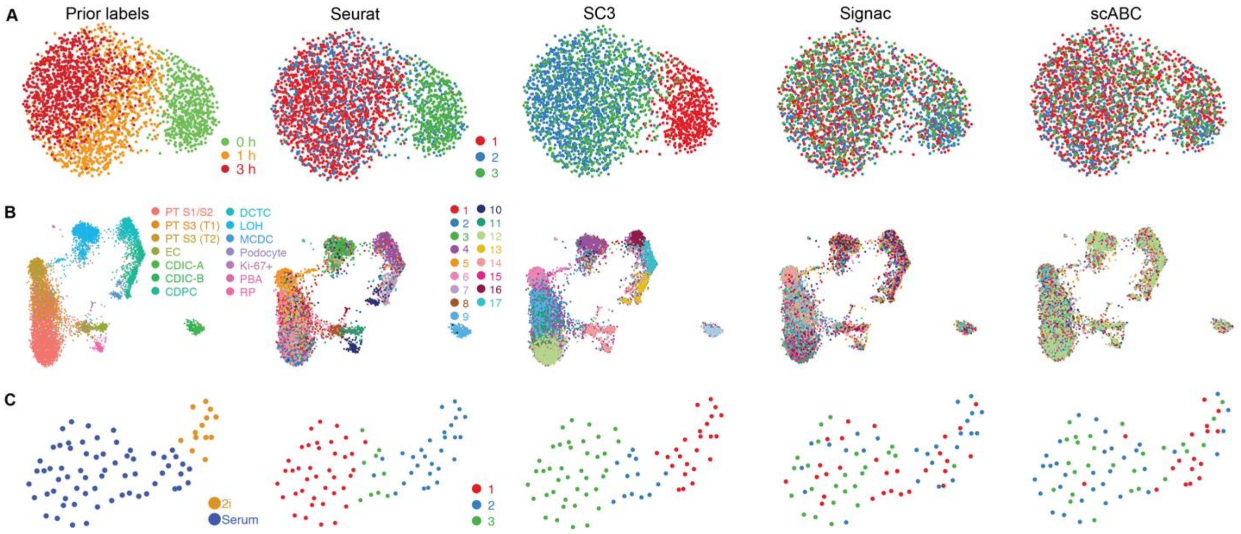

Figure S10. Comparison of scAI with other methods using single omics data on real datasets. (a) UMAP visualization of cells from A549 dataset. Cells were colored by experimental time information, and the clustering results from Seurat, SC3, Singac and scABC respectively. As scATAC-seq data was extremely sparse, all the UMAPs were performed using scRNA-seq data. **(b)** UMAP visualization of cells from kidney dataset. Cells were colored by the clustering results from the original study [1], Seurat, SC3, Singac and scABC respectively. **(c)** UMAP visualization of cells from mESC dataset. Cells were colored by cultured conditions, and the clustering results from Seurat, SC3, Singac and scABC respectively.

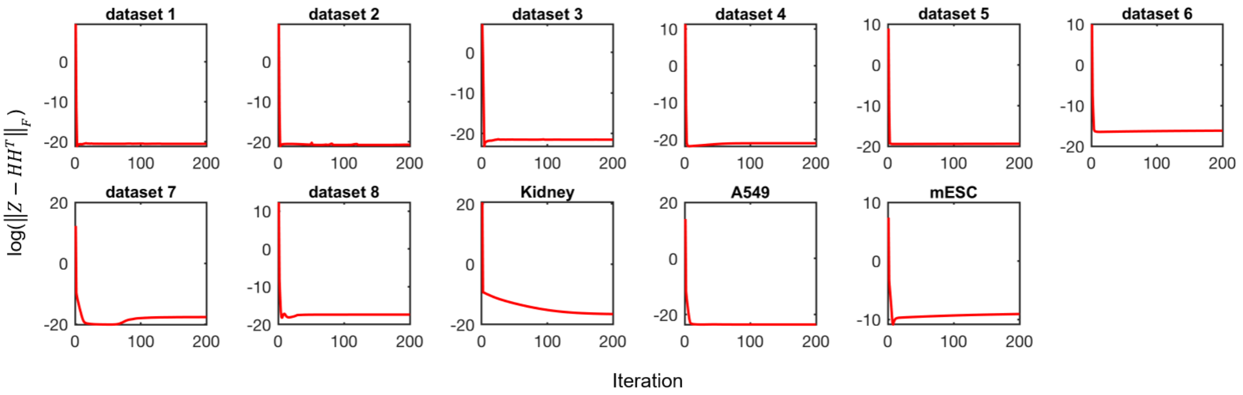

Figure S11. The evolution of the third term in Eq.1 (i.e., $\|\boldsymbol{Z} - \boldsymbol{H}\boldsymbol{H}^T\|_F$) on simulation and real datasets over the iterations. y-axis is the log-transformed $\|Z - HH^T\|_F$.

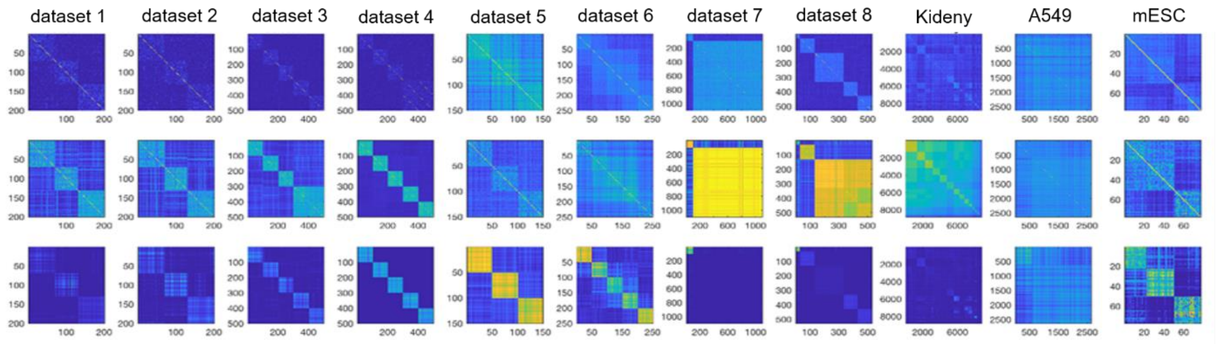

Figure S12. Heatmap of cell-cell similarity matrix based on scRNA-seq data (top row), similarity matrix based on aggregated single-cell epigenomic data (middle row), and the inferred similarity matrix *Z* by scAI (bottom row). The similarity matrices based on scRNA-seq data or aggregated single-cell epigenomic data were computed by Pearson's correlations.

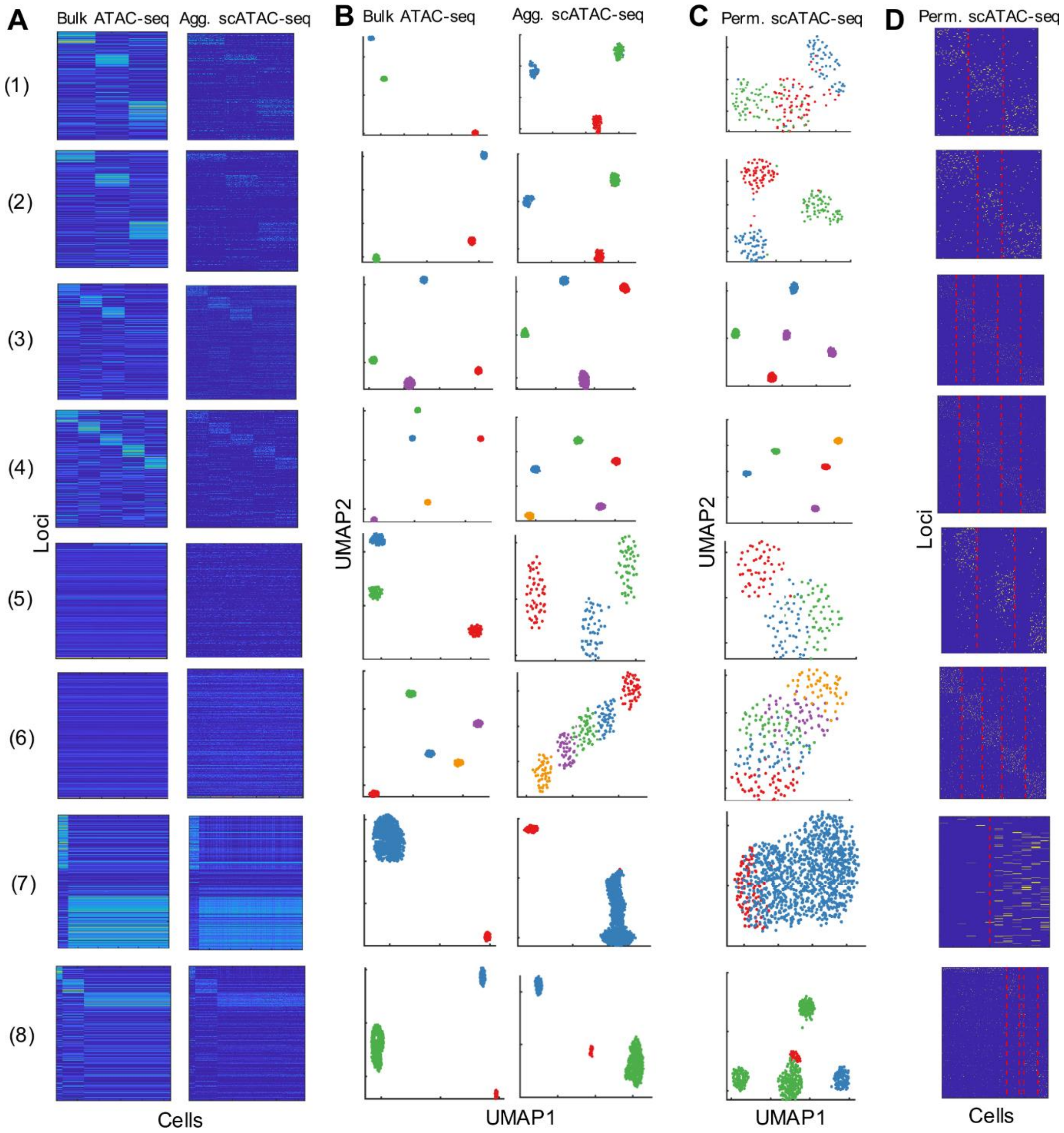

Figure S13. Aggregation performance of scAI on simulation datasets. (a) Heatmap of the bulk ATAC-seq data (i.e., raw ATAC-seq data prior to making them sparse and binarization (left), and the aggregated scATAC-seq data obtained from scAI (right). Rows are all the loci and columns are cells. In dataset 5 and dataset 6, no block structures were shown was because we do not know the ground truth of the loci loading matrix. **(b)** UMAP visualization of bulk ATAC-seq data (left) and aggregated scATAC-seq data obtained by applying scAI to scRNA-seq and scATAC-seq data (right). **(c)** UMAP visualization of aggregated scATAC-seq data obtained by applying scAI to scRNA-seq and permuted scATAC-seq. **(d)** Heatmap of differential accessible loci in the permuted scATAC-seq data *without* aggregation. Rows are the differential accessible loci and columns are cells.

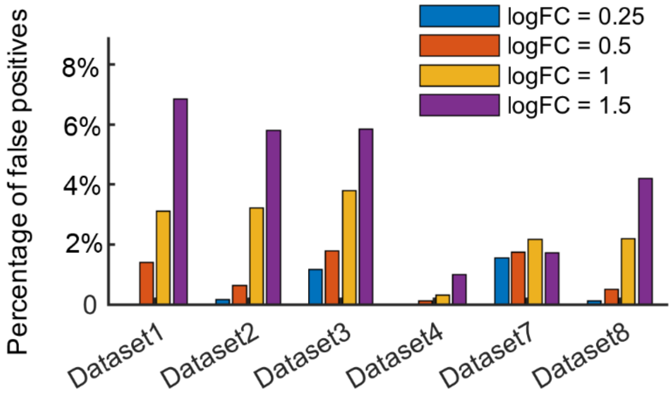

Figure S14. The false positive percentages of differential accessible loci on simulation datasets. Different colors represent different cases by varying the threshold of log fold change when identifying differential accessible loci. We calculated the percentage of false positive differential accessible loci based on the aggregated scATAC-seq data by comparing to the identified differential accessible loci based on the bulk ATAC-seq data. Specifically, the percentage of false positives was defined as the percentage of differential accessible loci that were not in the set of differential accessible loci based on the bulk ATAC-seq data.

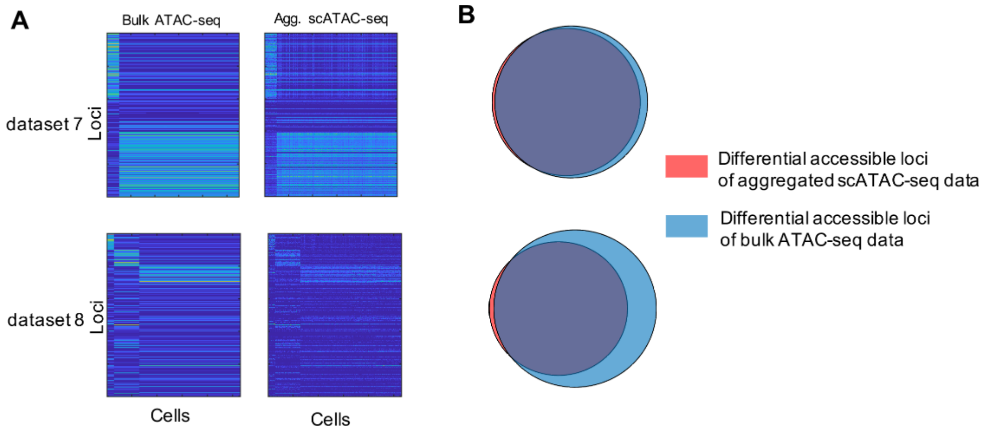

Figure S15. Performance of scAI on the two simulation datasets with imbalanced cluster sizes. (a) Heatmap of original scATAC-seq data prior to making them sparse and binarization (termed as bulk ATAC-seq) (left), and aggregated scATAC-seq data obtained from scAI (right). **(b)** Venn diagram showing the overlap of differential accessible loci of aggregated scATAC-seq data (red color) and differential accessible loci of bulk ATAC-seq data (blue color).

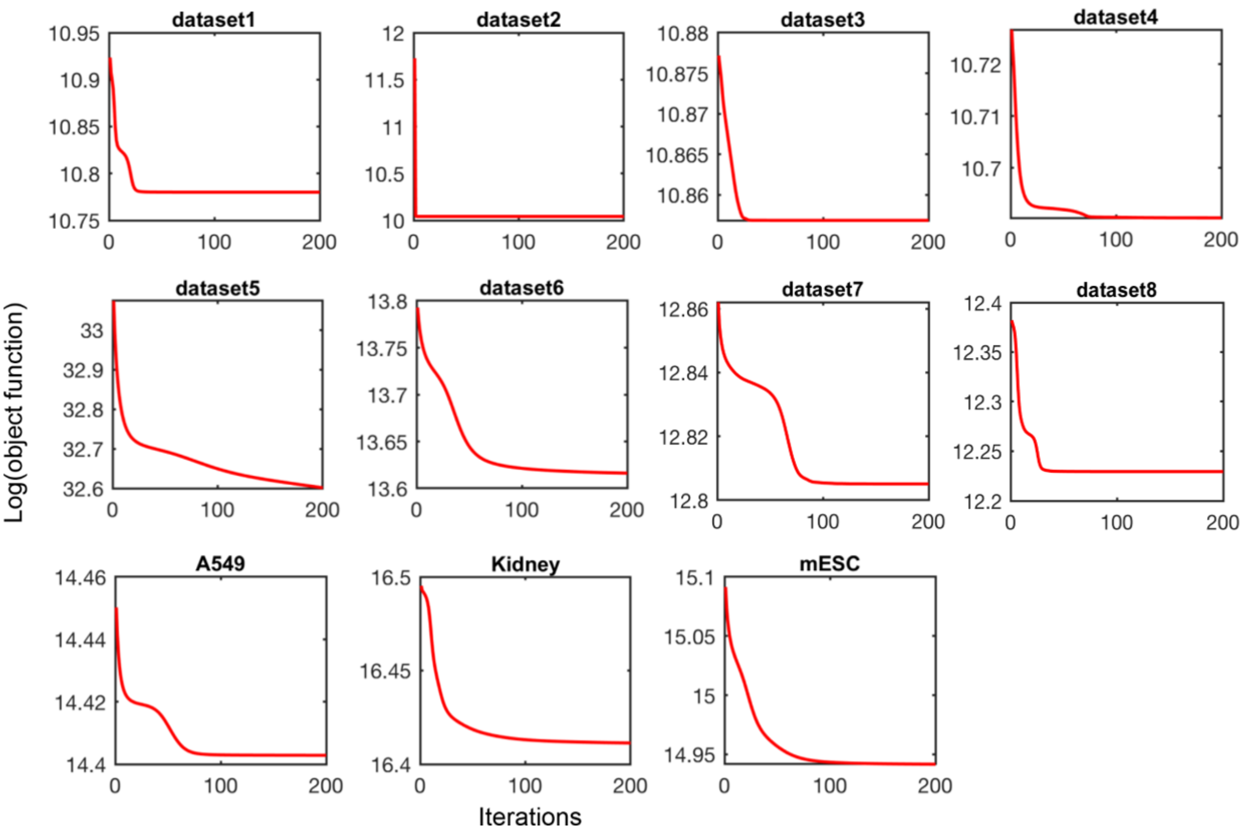

Figure S16. The evolution of objective function values with respect to the iterations of scAI algorithm on each dataset. y-axis is the log-transformed objective function.

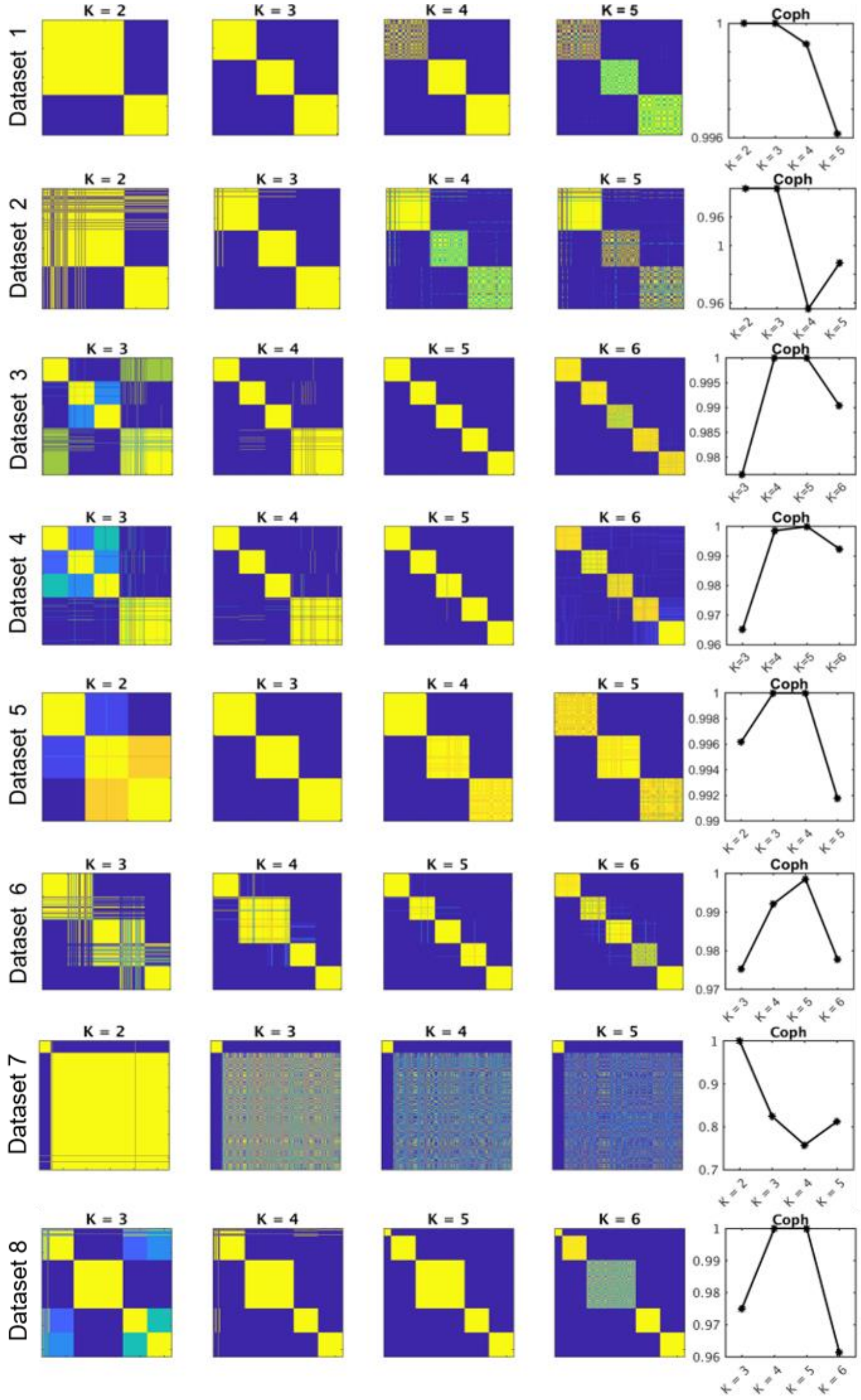

Figure S17. Rank selection of scAI on each simulation dataset based on the stability score *Coph*.

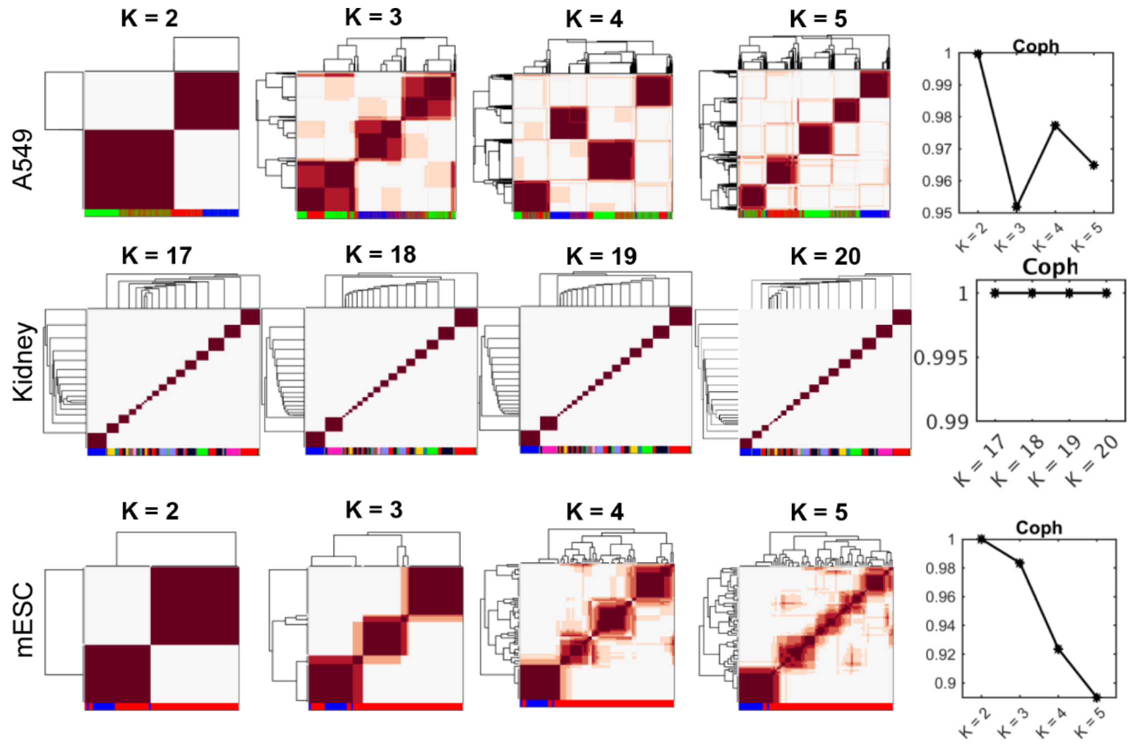

Figure S18. Rank selection of scAI on real datasets based on the stability score *Coph*. The colors at the bottom of the heatmap represent the prior/independent information of the cells, such as the experimental time (A549), cell labels assigned in the original study (kidney), and experimental conditions (mESC), respectively.

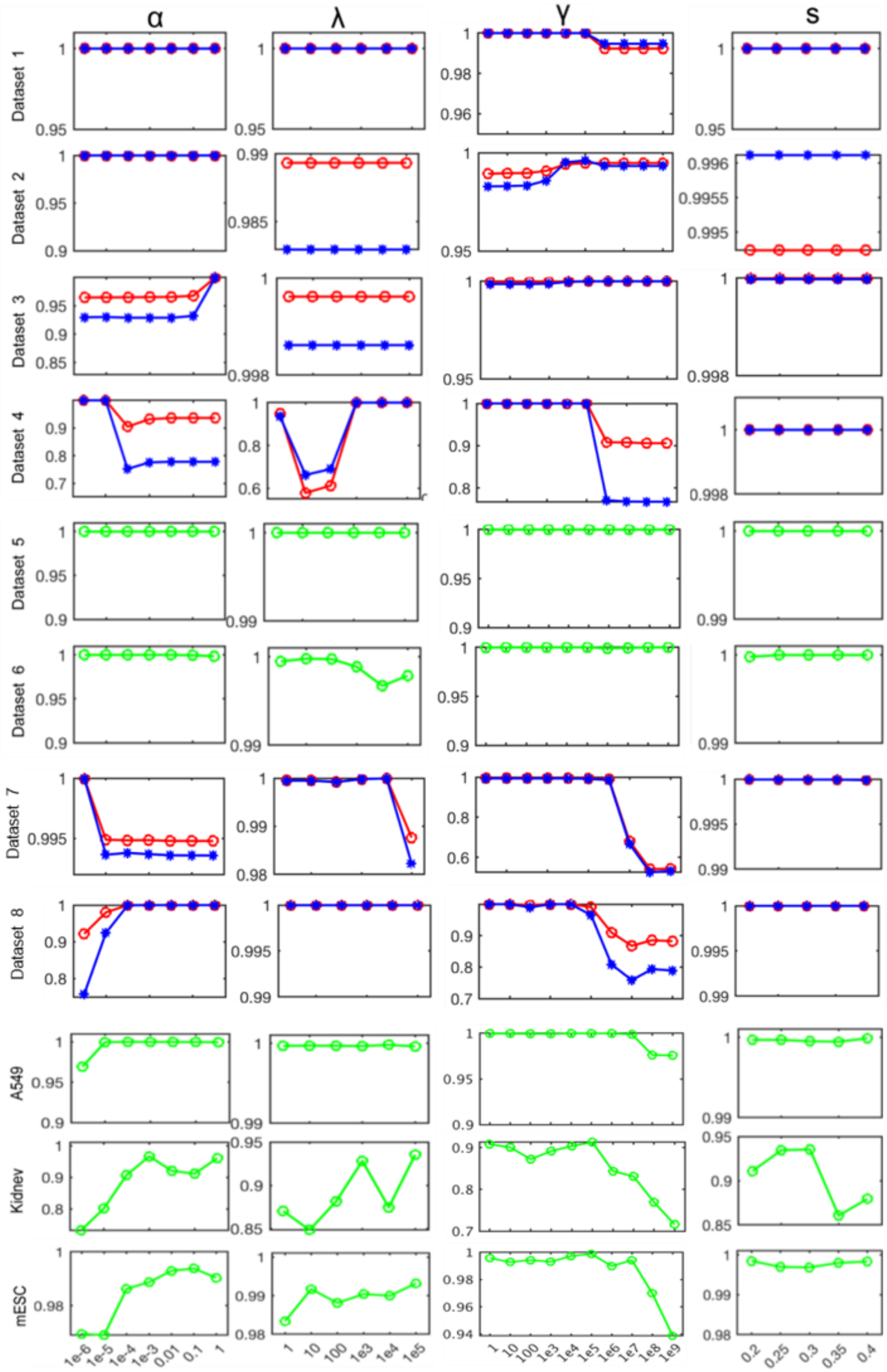

Figure S19. The performance of scAI when varying parameters on each dataset. Each column represents one parameter. The red, blue and green colors represent AUC, AUPR and *Coph*, respectively. Area Under receiver operating characteristic Curve (AUC) and Area Under Precision-Recall curve (AUPR) were calculated by comparing the reconstructed cell loading matrix and its ground truth.

Supplementary Tables

Table S1. Summary of simulation datasets.

Datasets	Data size	Note
Dataset 1	1000*200 (5000*200)	Different sparsity levels
Dataset 2	1000*200 (5000*200)	Different noise levels
Dataset 3	2000*500 (5000*500)	Some clusters that are defined from transcriptomic differences do not reflect epigenetic distinctions
Dataset 4	2000*500 (5000*500)	Some clusters that are defined from epigenetic differences do not reflect transcriptomic distinctions
Dataset 5	2500*150 (5000*150)	Three discrete clusters simulated from real dataset
Dataset 6	2500*250 (5000*250)	A continuous process simulated from real dataset
Dataset 7	1000*1100 (5000*1100)	Two imbalanced cell clusters in both scRNA-seq and scATAC-seq data, where one cluster has 100 cells and the other has 1000 cells.
Dataset 8	2000*530 (5000*530)	Five imbalanced cell clusters with five clusters in scRNA-seq data with 30,100,200,100,100 cells in each cluster and three clusters in scATAC-seq with 30,100,400 cells in each cluster.

These simulation datasets include eight scenarios with different transcriptomic/epigenomic properties. The numbers outside and inside brackets represent the size/dimensions of the simulated scRNA-seq and scATAC-seq data, respectively.

Table S2. Summary of parameters used in this study.

		Parameters	A549	Kidney	mESC
Data preprocessing	scRNA-seq	minCells	10	10	Null
		minCounts	500	500	
		maxCounts	9100	10e6 (default)	
		minFeatures	0 (default)	0 (default)	
		libararyflag	1 (default)	1 (default)	
		logNormalize	1 (default)	1 (default)	
	scATAC-seq	minCells	5	5	Null
		minCounts	200	200	
		maxCounts	10e6 (default)	10e6(default)	
		minFeatures	0 (default)	0 (default)	
		libararyflag	1 (default)	1(default)	
		logNormalize	1 (default)	1(default)	
Feature selection	scRNA-seq	condition	time	Null	Null
		sustem_used	Mac' (default)	Mac' (default)	
		r	0.05	0.25 (default)	
		fc	0.1	0.25 (default)	
		cutoff	0.05 (default)	0.05 (default)	
		flag	0 (default)	2	
		low_mu	0.01 (default)	0.01 (default)	
		high_mu	3.5 (default)	3.5 (default)	
		low_F	0.5 (default)	0.5 (default)	
	scATAC-seq	bin	null (default)	50kb	
Parameters in optimization model		K	2	20	3
		alpha	1 (default)	1 (default)	0.01
		lambda	100000	100000	1000
		gamma	1 (default)	1 (default)	100000
		s	0.25 (default)	0.25 (default)	0.25 (default)
		stop_rule	1 (default)	1 (default)	1 (default)
		repeat	10 (default)	1	10 (default)
		Inits	null (default)	null (default)	null (default)
		seeds	1~repeat (default)	1~repeat (default)	1~repeat (default)
Identification of factor/cluster-specific features	scRNA-seq	cutoff1	0.5 (default)	0.5 (default)	0.5 (default)
		cutoff2	0.5 (default)	0.5 (default)	0.5 (default)
		r	0.25 (default)	0.25 (default)	0.25 (default)
		fc	0.25 (default)	0.25 (default)	0.25 (default)
		cutoff	0.05 (default)	0.05 (default)	0.05 (default)
		n	10 (default)	10 (default)	10 (default)
	scATAC-seq	cutoff1	0.5 (default)	0.5 (default)	0.5 (default)
		cutoff2	0.5 (default)	0.5 (default)	0.5 (default)
		r	0.05	0.25 (default)	0.25 (default)
		fc	0.25 (default)	0.25 (default)	0.25 (default)
		cutoff	0.05 (default)	0.05 (default)	0.05 (default)
		n	10 (default)	10 (default)	10 (default)
Regulatory link inference		H_cutoff	0.1	Null	Null
		bin	500kb (default)		

References

1. Cao J, Cusanovich D, Ramani V, Aghamirzaie D, Pliner H, Hill AJ, Daza R, McFaline-Figueroa J, Packer J, Christiansen L, et al. Joint profiling of chromatin accessibility and gene expression in thousands of single cells. Science 2018;361:1380-5.
