# Supplementary material for: scAI: an unsupervised approach for the integrative analysis of parallel single-cell transcriptomic and epigenomic profiles
Source: Genome Biol. 2020 Feb 3;21:25. doi: 10.1186/s13059-020-1932-8 (PMC6996200; doi:10.1186/s13059-020-1932-8)
Supplement: Supplementary file 3 — Additional file 3. Review history. [file 13059_2020_1932_MOESM3_ESM.docx]

Review History

**First round of review**

**Reviewer 1**

**Were you able to assess all statistics in the manuscript, including the appropriateness of statistical tests used?**

No

**Were you able to directly test the methods?**

No

**Comments to author:**

In this manuscript, Lihua Zhang et al. proposed an unsupervised approach named scAI to integrate transcriptomic and epigenomic profiles from the same cell to deconvolute cellular heterogeneity of single cells. This manuscript is well structured and clearly written. The iterative learning methods has a solid mathematical derivation and its performance was validated by simulation and experimental data.

Understanding how to combine transcriptomic and epigenomic profiles is important for the understanding of cellular regulation of transcriptomes. The core question posed is relevant and timely. Overall, I think this work is suitable for the publication on GB. But there are still several issues need to be addressed:

1. A interesting question is how different between the similarity matrix of projecting cells after integrate epigenomic and the origin matrix Z? In addition, I also want to see a comparison with the methods use only scATAC-seq or scRNA-seq, like scABC, to fully evaluate the gain of such parallel profiling over single omics.

2. Data pre-processing has not been clearly described. And the processed data should be provided at the level of gene and cell type, not only links to original previously published datasets. It's better to give a detailed description on how to generate the import data for scAI from raw data. We all know that the pre-processing always influences the final results.

3. The authors should give some more detailed analysis of the hyper-parameters in equation (1). Why is much bigger than , does this mean that the influence of loading values of gene/locus, ie, W, is not important in the model?

4. On page 11 line 12, C2 and C7, and C8 and C10, show similar gene expression profiles, but strong differential accessibility pattern. And the following paragraph gives the explanation such as binding motifs. Can the authors try other software like chromVAR, which was specially design for scATAC-seq data, to compare the loci or related TFs?

5. The manuscript would benefit from language polishing and careful proofreading.

**Reviewer 2**

**Were you able to assess all statistics in the manuscript, including the appropriateness of statistical tests used?**

Yes

**Were you able to directly test the methods?**

No

**Comments to author:**

\## Summary of the paper

The authors proposed an unsupervised approach scAI, a matrix factorisation model, to integrate parallel transcriptomic and epigenomic profiles and to overcome the data sparsity by sharing epigenetic information across similar cells. The authors argue that the aggregation of epigenetic data enhances the signal and allows a coherent integration with expression data. This approach enables them to identify sub-populations based on multi-omics measurements. They also propose a visualisation strategy, VscAI, that simultaneously projects cells, genes and marker loci in a common 2D space. Generally, the paper is well written and motivated with a detailed discussion and comparison with related methods. The major contribution of this work is the introduction of the cell-cell similarity matrix Z which smooths the epigenetic data, however, I believe the simulation strategy could be performed in a more clear way and should capture edge cases of the proposed model, for instance highly imbalanced clusters sizes.

\## Major comments

1. In addition to inferring low-dimensional representations W1, W2 and H, the authors introduced a cell-cell similarity matrix Z to perform a weighted combination of epigenomic signals from similar cells (e.g. belonging to the same cluster). Although the idea is elegant, I wonder how will imbalanced cluster sizes will affect the proposed method, and whether the authors should have a scaled version of H. For instance, imagine that we have locus $i$ which has value 1 (i.e. open) on two cell subpopulations C1 (n_cells = 10) and C2 (n_cells = 100), and cells within clusters do not vary that much; so Z has two clear blocks, one much larger than the other. Wouldn't then this weighted average artificially make this locus as differentially accessible, e.g. the aggregated counts $X_{2}Z$ for locus $i$, could become $i_c1 = 10$ and $i_c2 = 100$? As I understand the R matrix does not solve this issue, it is mostly introduced to subsample the number of cells in total. When reading the simulation study details, the number of cells in each cluster are really similar. I believe the authors should provide a more realistic simulation study where the number of cells in each cluster varies, as we would expect in real single cell datasets.

2. Following on the previous remark, I wonder whether the proposed method makes the data look more distinct than they originally are? This mostly can be seen from the 3rd column of Figure 2a, where the aggregated scATAC-seq data are completely distinct across clusters, which cannot be explained even by the scRNA-seq data. If this is the case, I am concerned that this might introduce lots of false negative hits, e.g. when identifying differentially accessible regions across sub-populations. One quick way to visualise if clusters become artificially more distinct is to show the raw scATAC-seq data prior to making them sparse (and possibly prior to binarization). When visualised, how do the aggregated scATAC-seq data compare to them? Another approach would be to permute the data and test whether the method overfits or creates artificial clusters. Also, the choice of tSNE for visualisation of synthetic data might not be optimal (tSNE focuses mostly on retaining local distances), see my next point.

3. During the whole paper there is a mix of t-SNE and UMAP plots, which I believe make the whole paper confusing. Why the authors do not decide to show results using only one dimensionality reduction method, e.g. UMAP which also preserves global distances between clusters?

4. From Figure 2c it seems that the method performs perfect clustering in all simulation scenarios. I wonder whether more noise or the initial data should become less discriminative in each cluster, e.g. in Additional File 1 page 4, to add genes/loci that are expressed in more than one cluster. Also it would be nice to show a measure of false positives for the identifies marker genes (orange colour) and loci (yellow colour). Controlling for false positives is important, especially if the method is to be used to identify potential marker genes/loci!

\## Minor comments

1. Kidney dataset analysis: you mention that the scAI method identified 17 subpopulations. However, from Figure S3, 2nd row the Coph score and the consensus matrix show that potentially there exist more clusters, always according to your proposed method. Could the authors explain the reasoning behind this choice, was it mostly biologically driven, so it was easier to match with the previous study?

2. A549 cells analysis: The VscAI plots provide an intuitive way to interpret the data. However, in Figure 4d (right subfigure) the same loci are shown in two opposite positions in the plot (e.g. SMAD3 and NR3C1). Could the authors explain what would this mean?

3. For mESCs dataset, how did the authors treated missing values for the methylation data? Compared to scRNA-seq and scATAC-seq a 0 in scBS-seq data mean unmethylated CpG sites and not missing values. For instance, probabilistic approaches (such as MOFA) would not include those regions when computing the likelihood. How does scAI cope with this during optimisation?

4. On page 3 line 59, this sentence seems incomplete, and does not really explain the reason why the existing methods will have bad performance when aligning multi-omics datasets.

5. In Figure S4, when increasing the \lambda parameter (which controls the sparseness of H) the performance of scAI in terms of AUC/AUPR increases as well. Wouldn't the opposite trend make more sense, or do you believe this has to do with overfitting of your model?

6. We thank the authors for making their code publicly available on github. From the github page I could see only two examples available. For reproducibility purposes it would be desirable to provide all details/code the authors used in the paper. Although I understand these details could be added after the paper has passed the review process.

\## Responses questions

- Are the methods appropriate to the aims of the study, are they well described, and are necessary controls included?

Yes

- Are the conclusions adequately supported by the data shown?

Mostly yes, although the simulation study should be conducted with more realistic scenarios, such as different cluster sizes.

- Are sufficient details provided to allow replication and comparison with related analyses that may have been performed?

Not for all analyses performed in the paper.

- Is the method likely to be of broad utility? Is any software component easy to install and use? Please indicate briefly the novel features and/or advantages of the method, and/or please reference the relevant publications and which methods, if any, it should be compared with.

The method will likely find adoption in the community, since it is one of the first tailored on parallel single cell multi-omics (e.g. scMT and sciCAR). The software is available on github and has walkthrough examples on how to use the software. The novel feature of the method is that it performs aggregation to increase signal of epigenetic data and all required quantities are inferred in a joint model. The authors also introduce a novel approach to visualise genes, loci and cells in the same 2D space, which might be really useful for interpreting multi-omics data.

- Is the paper of broad interest to others in the field, or of outstanding interest to a broad audience of biologists?

Yes, as the field progresses in profiling multiple molecular layers from the same single cell, integrative methods such as scAI will be relevant.

**Authors Response**

**Point-by-point responses to the reviewers’ comments:**

Reviewer reports

Reviewer #1

In this manuscript, Lihua Zhang et al. proposed an unsupervised approach named scAI to integrate transcriptomic and epigenomic profiles from the same cell to deconvolute cellular heterogeneity of single cells. This manuscript is well structured and clearly written. The iterative learning methods has a solid mathematical derivation and its performance was validated by simulation and experimental data.

Understanding how to combine transcriptomic and epigenomic profiles is important for the understanding of cellular regulation of transcriptomes. The core question posed is relevant and timely. Overall, I think this work is suitable for the publication on GB. But there are still several issues need to be addressed:

*Response: We thank the reviewer very much for the careful reading of our manuscript and the insightful suggestions. Below please find our detailed responses to the reviewer’s comments. Multiple minor changes were introduced throughout the manuscript and all changes were highlighted in red in the revised manuscript, including both main text and Additional files.*

1. An interesting question is how different between the similarity matrix of projecting cells after integrate epigenomic and the origin matrix Z? In addition, I also want to see a comparison with the methods use only scATAC-seq or scRNA-seq, like scABC, to fully evaluate the gain of such parallel profiling over single omics.

*Response: Thanks for the nice suggestion.*

*For the point one, on the difference between the similarity matrix of projecting cells after integrating epigenomic profile and the origin matrix Z, we would like to clarify that the original matrix Z is first randomly generated in the algorithm, and then it is updated during the iterative learning process. In the revised manuscript, we added a new Supplementary Figure S15 in Additional file 2 to present evolution of the third term in Eq.1 (i.e., ‖Z-〖HH〗^T ‖_F), which quantifies the difference between the similarity matrix of projecting cells and the matrix Z. For both simulation and real datasets, the differences converge to zeros over the iteration, as expected.*

*Secondly, if there are subpopulations with distinct transcriptomic profiles but similar epigenomic profiles, then the similarity matrix of cells calculated using aggregated epigenomic data should be very different from the cell-cell similarity matrix Z of projecting cells. One such example is the simulation dataset 3. If there are subpopulations with distinct epigenomic profiles but similar transcriptomic profiles, then the similarity matrix of cells before integrating epigenomic profile should be very different from the one after integrating epigenomic profile. One such example is the simulation dataset 4. However, if all the subpopulations exhibit differences in both transcriptomic and epigenomic profiles, the two similarity matrices should be similar. To study this point, in the revised manuscript, we computed the Pearson’s correlations between cells using only scRNA-seq or aggregated epigenomic data and compared them with the cell-cell similarity matrix Z of projecting cells after integrating epigenomic data, as shown in a new Supplementary Figure S16 in Additional file 2. In the revised manuscript, we added this point in the second paragraph in Discussion (page 25) and the subsection “Comparison of cell-cell similarity matrix” in Additional file 1 (page 9).*

*In the light of the reviewer’s suggestion, in the revision we performed comparisons with methods designed only for scRNA-seq or scATAC-seq data. Specifically, we first compared scAI with two methods on scRNA-seq data, including Seurat and SC3. Seurat uses a community detection method and SC3 uses a consensus approach to identify cell clusters. We then compared scAI with two methods on scATAC-seq data, including Signac (https://satijalab.org/signac/)) and scABC (https://github.com/SUwonglab/scABC). Signac is an extension of Seurat for the analysis of scATAC-seq data. scABC uses a statistical approach to analyze scATAC-seq data. On simulation datasets, we evaluated the performance of cell clustering using normalized mutual information (NMI). On real datasets, we compared the clustering based on those four methods with prior labels using UMAP.*

*On simulation datasets, we observed comparable NMI values between scAI and SC3, but slightly lower values of Seurat (Additional file 2 Figure S13). For the clustering of scATAC-seq data, both Signac and scABC showed significantly lower NMI values compared to those by scAI using both scRNA-seq and scATAC-seq data.*

*On A549 real datasets (new Supplementary Figure S14), by visualizing cells in the UMAP space, we found that both Seurat and SC3 were unable to detect the transition stage and distinguish cells from 1 and 3 hours by performing cell clustering. Cell clusters identified by Signac and scABC were completely mixed in the UMAP space. On Kidney dataset, Seurat was unable to distinguish the DCTC cells and CDPC cells, and Signac and scABC were unable to reveal the underlying data structure, which produced incorrect cell clusters. On mESC dataset, while both Seurat and SC3 correctly identified the cell clusters, the clusters identified by Signac and scABC were still mixed together in the UMAP space.*

*Overall scAI is able to consistently identify the expected clusters and also the clusters with subtle transcriptomic differences but strong chromatin accessibility differences (as shown in Kidney dataset). Taken together, these comparison results demonstrated the importance of integrating parallel single cell multiomics data. These results have been added in a new subsection of the Result entitled “Comparison with methods using single omics data” on page 24 with two new Supplementary Figures S13 and S14. The detailed description of how these comparisons were performed has been added in “Details of method comparisons on single omics data” in Additional file 1 (page 10).*

2. Data pre-processing has not been clearly described. And the processed data should be provided at the level of gene and cell type, not only links to original previously published datasets. It's better to give a detailed description on how to generate the import data for scAI from raw data. We all know that the pre-processing always influences the final results.

*Response: In original manuscript, the detailed description of data pre-processing was shown in Supplemental Methods in Additional File 1 (page 6) and the parameters used in the pre-processing procedure were summarized in Supplementary Table S2 in Additional File 2. To make our computational work even more transparent, in the revision we added the workflow of running scAI on all three real datasets, including the processed data, which now be accessed at Github (https://github.com/sqjin/scAI and https://github.com/amsszlh/scAI).*

3. The authors should give some more detailed analysis of the hyper-parameters in equation (1). Why \lamba is much bigger than \alpha, does this mean that the influence of loading values of gene/locus, ie, W, is not important in the model?

*Response: We would like to thank the reviewer for raising this important point. The different magnitudes of parameter values are dependent on the magnitudes of different terms in Equation (1). Since H is scaled by row, the entry of matrix H is less than 1. Thus, the magnitude of this term is small. \lambda usually is large to ensure the importance of this term. The parameter \alpha is set to be small because the magnitude of this term is usually relatively large. That is not mean W is not important in the model. In the revised manuscript, we added these explanations in Method section entitled “Optimization algorithm for scAI” on page 29.*

4. On page 11 line 12, C2 and C7, and C8 and C10, show similar gene expression profiles, but strong differential accessibility pattern. And the following paragraph gives the explanation such as binding motifs. Can the authors try other software like chromVAR, which was specially design for scATAC-seq data, to compare the loci or related TFs?

*Response: We thank the reviewer for this great suggestion. In the revision, we applied chromVAR to analyzing differential accessible loci between C2 and C7, and C8 and C10, respectively. chromVAR calculates the bias corrected deviations in accessibility. For each motif, there is a value for each cell, which measures how different the accessibility for loci with that motif is from the expected accessibility based on the average of all the cells. By performing hierarchical clustering of the calculated deviations of top 30 most variable TFs, we found that these TFs were divided into two clusters, and each TF cluster was specific to one particular cell subpopulation, which was found to be consistent with the clustering by scAI (new Additional file 2 Figure S12). In the revised manuscript, we have added these results in the Results on page 12 with new Supplementary Figure S12 in Additional File 2.*

5. The manuscript would benefit from language polishing and careful proofreading.

*Response: We apologize for not being more careful in proofreading and language polishing. In the revision, we have made significant effort in these two areas.*

Reviewer #2

\## Summary of the paper

The authors proposed an unsupervised approach scAI, a matrix factorisation model, to integrate parallel transcriptomic and epigenomic profiles and to overcome the data sparsity by sharing epigenetic information across similar cells. The authors argue that the aggregation of epigenetic data enhances the signal and allows a coherent integration with expression data. This approach enables them to identify sub-populations based on multi-omics measurements. They also propose a visualisation strategy, VscAI, that simultaneously projects cells, genes and marker loci in a common 2D space. Generally, the paper is well written and motivated with a detailed discussion and comparison with related methods. The major contribution of this work is the introduction of the cell-cell similarity matrix Z which smooths the epigenetic data, however, I believe the simulation strategy could be performed in a more clear way and should capture edge cases of the proposed model, for instance highly imbalanced clusters sizes.

*Response: We thank the reviewer very much for the careful reading of our manuscript and the insightful suggestions. Below please find our detailed responses to the reviewer’s comments. All changes were highlighted in red in the revised manuscript, including both main text and Additional files.*

\## Major comments

1. In addition to inferring low-dimensional representations W1, W2 and H, the authors introduced a cell-cell similarity matrix Z to perform a weighted combination of epigenomic signals from similar cells (e.g. belonging to the same cluster). Although the idea is elegant, I wonder how will imbalanced cluster sizes will affect the proposed method, and whether the authors should have a scaled version of H. For instance, imagine that we have locus $i$ which has value 1 (i.e. open) on two cell subpopulations C1 (n_cells = 10) and C2 (n_cells = 100), and cells within clusters do not vary that much; so Z has two clear blocks, one much larger than the other. Wouldn't then this weighted average artificially make this locus as differentially accessible, e.g. the aggregated counts $X_{2}

Z$ for locus $i$, could become $i_c1 = 10$ and $i_c2 = 100$? As I understand the R matrix does not solve this issue, it is mostly introduced to subsample the number of cells in total. When reading the simulation study details, the number of cells in each cluster are really similar. I believe the authors should provide a more realistic simulation study where the number of cells in each cluster varies, as we would expect in real single cell datasets.

*Response: We thank the reviewer for these insightful comments and suggestions. In implementing scAI in our original manuscript, we actually scaled H with the sum of each row equaling 1 in each iteration step. In addition, we generated aggregated scATAC-seq profile after normalizing Z◦R with the sum of each column equaling 1. These two strategies would likely make the solution not affected by imbalanced cluster sizes. We now clarify this important information in the Results entitled “Overview of scAI” (page 5) and Methods entitled “Optimization algorithm for scAI” (page 29).*

*In the revision we have provided two additional simulation datasets with imbalanced cluster sizes to further illustrate the performance of scAI. One dataset (referred as simulation dataset 7) has two clusters in both scRNA-seq and scATAC-seq data, where one cluster has 100 cells and the other has 1000 cells. The other dataset (referred as simulation dataset 8) has five clusters in scRNA-seq data with 30, 100, 200, 100, 100 cells in each cluster, and three clusters in scATAC-seq data with 30, 100, 400 cells in each cluster. In addition, different sparsity levels and noise levels have been used to evaluate the performance. The detailed description of the simulations was added in Supplementary Method section in Additional File 1 (page 6) and Supplementary Table S1 in Additional File 2 (page 16).*

*For these two new datasets with imbalanced cluster sizes, we performed the same analyses as other simulation datasets and added the results in the updated Figure 2. The new analyses further demonstrate the effectiveness of scAI, including the low-dimensional representation and visualization and the identification of cell clusters. Specially, (1) by visualizing cells in the UMAP space using the aggregated scATAC-seq data obtained from scAI, we clearly observed two clusters in dataset 7 and three clusters in dataset 8 (the last two rows of the updated Figure 2a). In particular for dataset 8, three cell clusters were observed in the UMAP space of both scATAC-seq and aggregated scATAC-seq data (the eighth row of Fig. 2a). However, five cell clusters were well distinguished after integrating with scRNA-seq data, as shown in the VscAI space (the eighth row of Fig. 2b). (2) By quantifying the accuracy of the reconstructed low-dimensional representations, including cell loading, gene loading and locus loading matrices, scAI was shown to be able to accurately reconstruct the high-dimensional data (the last two rows of the updated Figure 2c). These accurate reconstructions were found robust to different sparsity levels as seen in the dataset 7 and different noise levels as seen in the dataset 8. The accuracy of low-dimensional representations of the high-dimensional data is crucial to the downstream analysis, such as cell clustering based on the cell loading matrix H and important genes/loci identification based on the gene/locus loading matrix W1/W2. (3) Applying MOFA to these two datasets, we found that both scAI and MOFA accurately identified the cell clusters on dataset 7, but scAI performed better than MOFA on dataset 8. These results were added in the Results entitled “Model validation and comparison using simulated data” (page 9) with the updated Figure 2. In addition, we also updated some other Supplementary figures related with simulation datasets to add the corresponding results of these two new datasets, including Figure S1, Figure S2, Figure S3 and Figure S4.*

*Moreover, we also compared the heatmap of the aggregated scATAC-seq data and the raw ATAC-seq data prior to making them sparse and binarization (termed as bulk ATAC-seq data hereafter), and observed the same loci patterns (new Supplementary Figure S19a). Furthermore, we identified differential accessible loci with Wilcoxon rank sum test (adjusted p-values < 0.01, log fold-changes > 0.5) on bulk ATAC-seq and aggregated scATAC-seq, respectively. These two sets of differential accessible loci were found to be highly overlapped (new Supplementary Figure S19b). These results showed that the aggregation of scAI would not artificially make loci as differentially accessible in a high proportion. The investigate of this concern was further detailed in the response to comments 2 and 4. These results were added in a new paragraph in Discussion (page 26) with one new Supplementary Figure S19 in Additional File 2.*

2. Following on the previous remark, I wonder whether the proposed method makes the data look more distinct than they originally are? This mostly can be seen from the 3rd column of Figure 2a, where the aggregated scATAC-seq data are completely distinct across clusters, which cannot be explained even by the scRNA-seq data. If this is the case, I am concerned that this might introduce lots of false negative hits, e.g. when identifying differentially accessible regions across sub-populations. One quick way to visualise if clusters become artificially more distinct is to show the raw scATAC-seq data prior to making them sparse (and possibly prior to binarization). When visualised, how do the aggregated scATAC-seq data compare to them? Another approach would be to permute the data and test whether the method overfits or creates artificial clusters. Also, the choice of tSNE for visualisation of synthetic data might not be optimal (tSNE focuses mostly on retaining local distances), see my next point.

*Response: We would like to thank the reviewer for raising this important question. We guess the “false negatives” mentioned here are actually meant to be the “false positives”. The 3rd column of original Figure 2a is the tSNE plot of aggregated scATAC-seq data. In the revision, we also used UMAP plot for the aggregated scATAC-seq (updated Figure 2a), and we also found that the aggregated scATAC-seq data are distinct across clusters.*

*We compared the aggregated scATAC-seq data to the raw ATAC-seq data prior to making them sparse and binarization (termed as bulk ATAC-seq data hereafter) in two ways: the direct visualization of loci patterns using heatmap and the low-dimensional visualization of cells using UMAP. As shown in the heatmap (new Supplementary Figure S17a), the bulk ATAC-seq data and the aggregated scATAC-seq data exhibit the same loci patterns. As shown in the UMAP space (new Supplementary Figure S17b), both bulk ATAC-seq and aggregated scATAC-seq data were found to be distinct across clusters. These observations were consistent across all the simulation datasets including the original six datasets and additional two new datasets with imbalanced cluster sizes.*

*To follow reviewer’s suggestion, we also randomly permuted scATAC-seq data across all cells. Then we applied scAI to the scRNA-seq data and the permuted scATAC-seq data. We found that the aggregated permuted scATAC-seq data were still distinct across clusters in some cases in the UMAP space (new Supplementary Figure S17 (c)), partly because there were still differential accessibility patterns across these clusters after permutation without aggregation, as clearly indicated in the heatmap of differential accessible loci in the permuted scATAC-seq data without aggregation in Supplementary Figure S17 (d). We also considered an extreme case where all the values of scATAC-seq data are equal and found scAI didn’t produce any artificial clusters. This is very likely caused by our normalization strategy, in which scAI aggregates scATAC-seq profile after normalizing Z◦R with the sum of each column equaling 1. On the other hand, scAI is able to identify cell clusters with high accuracy on all simulation datasets (Figure 2e). Our analysis suggests scAI robustly maintains cellular heterogeneity within and between different subpopulations when it enhances epigenomic signals.*

*In the revised manuscript, we added one new Supplementary Figure S17 in Additional file 2 to address this point, and added one new paragraph on this issue in the Discussion (page 26).*

3. During the whole paper there is a mix of t-SNE and UMAP plots, which I believe make the whole paper confusing. Why the authors do not decide to show results using only one dimensionality reduction method, e.g. UMAP which also preserves global distances between clusters?

*Response: In the light of the reviewer’s suggestion, we replaced all the tSNE plot by the UMAP plot in the main text. For the purpose of comparisons, we moved the tSNE plots in Figure 2 to a new Supplementary Figure S10.*

4. From Figure 2c it seems that the method performs perfect clustering in all simulation scenarios. I wonder whether more noise or the initial data should become less discriminative in each cluster, e.g. in Additional File 1 page 4, to add genes/loci that are expressed in more than one cluster. Also it would be nice to show a measure of false positives for the identifies marker genes (orange colour) and loci (yellow colour). Controlling for false positives is important, especially if the method is to be used to identify potential marker genes/loci!

*Response: We thank the reviewer for the nice suggestions. First, we apologize for the confusion for the Figure 2c legend. In Figure 2c, the AUC values measure the accuracy of the reconstructed cell loading (blue color), gene loading (orange color) and locus loading (yellow color) matrices, respectively. The accuracy of these reconstructed low-rank matrices is crucial to the downstream analysis, such as cell clustering based on the cell loading matrix and factor-specific genes/loci identification based on the gene/locus loading matrix. Thus, the figure legend in Figure 2c should be the reconstructed cell loading (blue color), gene loading (orange color) and locus loading (yellow color) matrices. We corrected this in the revised manuscript.*

*Moreover, to study whether stronger noise or the initial data with less discriminative patterns have effects on the performance of scAI, we added stronger noise and sparsity levels, and also made the initial data less discriminative among clusters by increasing the parameter value coph, on the new simulation dataset 8 with imbalanced cluster sizes. Of note, dataset 8 has five clusters in scRNA-seq data and three clusters in scATAC-seq data. We found that the noise levels and parameter coph values have little effects on the reconstructed cell loading, gene loading and locus loading matrices, as shown in the new Supplementary Figure S11. The sparsity level affects the performance if it is larger than some threshold (e.g., the sparsity of scRNA-seq and scATAC-seq data is larger than 98.9% and 99.5% respectively), as shown in the new Supplementary Figure S11. We added these results in the Results (page 9).*

*We agree that false positive control is important for differential expression/accessibility analysis. As shown in the new Figure S17a, scAI does not create any artificial clusters and does not make the data look more distinct than they originally are in the bulk ATAC-seq data. Thus we believe that scAI does not introduce high portion of false positives when performing differential accessibility analysis using aggregated scATAC-seq data. To further quantify the induced false positives, we calculated the percentage of false positive differential accessible loci based on the aggregated scATAC-seq data by comparing to the identified differential accessible loci based on the bulk ATAC-seq data. Specifically, the percentage of false positives was defined as the percentage of differential accessible loci that were not in the set of differential accessible loci based on the bulk ATAC-seq data. Such calculation provides an unbiased way to test whether the aggregation of scAI will induce false positives, regardless of the methods used for differential accessibility analysis. We adopt the Wilcoxon rank sum test for accessibility of cells in each cell subpopulation and the remaining cells. We found that the percentage of false positive differential accessible loci is less than 2% under typical parameters (adjusted p-values < 0.01, log fold-changes > 0.25). When increasing the threshold of log fold-changes from 0.25 to 1.5 in the differential accessibility analysis of both aggregated scATAC-seq data and bulk ATAC-seq data, the percentage of false positives ranged from 0% to 7% correspondingly, as shown in new Supplementary Figure S18. This analysis indicates that the aggregation strategy of scAI has a good control of false positives for the further differential accessibility analysis. We added these results in the Discussion (page 26) with a new Supplementary Figure S18 in Additional file 2.*

\## Minor comments

1. Kidney dataset analysis: you mention that the scAI method identified 17 subpopulations. However, from Figure S3, 2nd row the Coph score and the consensus matrix show that potentially there exist more clusters, always according to your proposed method. Could the authors explain the reasoning behind this choice, was it mostly biologically driven, so it was easier to match with the previous study?

*Response: Thanks for raising this point. The Coph score and the consensus matrix were used to evaluate the stability of scAI in reconstructing cell loading matrix H with different initial values for a given rank. In addition, as the entries of the consensus matrix were built based on the maximum value of columns of H, the rank with highest Coph score and clear block structures in the consensus matrix may not be the number of clusters on some datasets. Based on the stability parameter Coph, we set the rank to 20. By applying Leiden algorithm on the cell loading matrix H, we identified 17 clusters with the default resolution parameter equaling 1. This result was mostly biologically driven and based on the differential gene expression and loci accessibility analysis. Similar to Scanpy (Wolf et al., Genome Biology, 2018) and Seurat (Stuart et al., Cell, 2019), two of the most popular scRNA-seq data analysis tools, the number of subpopulations is determined by the resolution parameter in the community detection algorithm. How to determine the optimal number of clusters, in particular for large and complex datasets, still remains challenging. We clarified these details in a new section in the Additional file 1 entitled “Details of clustering analysis on kidney dataset” on page 7.*

2. A549 cells analysis: The VscAI plots provide an intuitive way to interpret the data. However, in Figure 4d (right subfigure) the same loci are shown in two opposite positions in the plot (e.g. SMAD3 and NR3C1). Could the authors explain what would this mean?

*Response: Thanks for pointing this out. In the left subfigure of Figure 4d, the selected marker genes were shown. Correspondingly, the loci should be shown in the right subfigure. Here we used the motifs instead of individual loci for easier understanding. The presented loci were the loci that were located within 10kb of marker genes’ regulatory regions, which were extracted from the database (http://bioinfo.life.hust.edu.cn/hTFtarget/) in lung tissue. Therefore, the same motifs such as SMAD3 and NR3C1 were shown in two opposite positions as they are enriched in different loci. We added this explanation in the figure legend of Figure 4d (page 18).*

3. For mESCs dataset, how did the authors treated missing values for the methylation data? Compared to scRNA-seq and scATAC-seq a 0 in scBS-seq data mean unmethylated CpG sites and not missing values. For instance, probabilistic approaches (such as MOFA) would not include those regions when computing the likelihood. How does scAI cope with this during optimisation?

*Response: The methylated levels of promoter, enhancer and CpG sites usually have negative correlations with gene expression levels. Since scAI aims at extracting similar patterns of genes and loci, the methylation data matrix was constructed in which the element was 0 if locus i was methylated in cell j or methylation information was missing; if locus i was unmethylated in cell j, the element was 1. Thus the unmethylated CpG sites were treated as 1, and the missing CpG sites were treated as 0 when applying scAI. In this way, the missing values of the input matrices (both single cell RNA and DNA methylation data) are 0. We added these details in the Supplemental Methods section in Additional file 1 (page 7).*

4. On page 3 line 59, this sentence seems incomplete, and does not really explain the reason why the existing methods will have bad performance when aligning multi-omics datasets.

*Response: Thanks for pointing it out. It now reads as “Recently, Seurat (version 3) [20] and LIGER [21] were developed for integrating scRNA-seq and single-cell epigenomic data. Both of these methods first transform the epigenomic data into a synthetic scRNA-seq data through estimating a "gene activity matrix", and then identify ‘anchors’ between this synthetic data and scRNA-seq data through aligning them in a low-dimensional space. The gene activity matrix is created by simply summing all counts within the gene body +2kb upstream. Such strategy may introduce improper synthetic data due to complex transcriptional regulatory mechanisms between gene expression and chromatin accessibility. The improper synthetic data may further lead to imperfect alignment when they are applied to parallel transcriptomic and epigenomic profiles, and likely affect downstream analysis.” We added these sentences in the Background section (page 3).*

5. In Figure S4, when increasing the \lambda parameter (which controls the sparseness of H) the performance of scAI in terms of AUC/AUPR increases as well. Wouldn't the opposite trend make more sense, or do you believe this has to do with overfitting of your model?

*Response: We guess the reviewer meant to say ‘\gamma’ parameter. The parameter \gamma controls the sparseness of H. The block structures of the inferred cell-cell similarity matrix Z will become more obvious if the sparsity of H increases to some extent. From this point, scAI will do better in terms of AUC/AUPR to some extent when increasing parameter \gamma. However, if H is too sparse, it might lead to decreased AUC/AUPR values, such as in the simulation datasets 1 and 4, as shown in the updated Supplementary Figure S4. In the revised manuscript, we updated the Supplementary Figure S4 using a large range of \gamma in Additional file 2. We also added this explanation in Additional file 1 (page 3).*

6. We thank the authors for making their code publicly available on github. From the github page I could see only two examples available. For reproducibility purposes it would be desirable to provide all details/code the authors used in the paper. Although I understand these details could be added after the paper has passed the review process.

*Response: Thank you for bringing this up. In the revision we have now added more walkthroughs of the simulation and real datasets in Github repositories (R package: https://github.com/sqjin/scAI and MATLAB package: https://github.com/amsszlh/scAI). We would like to mention that all the figures presented in the manuscript were generated in MATLAB environment. For reproducibility purposes, we used MATLAB live script in a single interactive environment, which stores and displays output alongside the code that creates it. The results can also be reproduced in the R package.*

\## Responses questions

- Are the methods appropriate to the aims of the study, are they well described, and are necessary controls included?

Yes

- Are the conclusions adequately supported by the data shown?

Mostly yes, although the simulation study should be conducted with more realistic scenarios, such as different cluster sizes.

- Are sufficient details provided to allow replication and comparison with related analyses that may have been performed?

Not for all analyses performed in the paper.

- Is the method likely to be of broad utility? Is any software component easy to install and use? Please indicate briefly the novel features and/or advantages of the method, and/or please reference the relevant publications and which methods, if any, it should be compared with.

The method will likely find adoption in the community, since it is one of the first tailored on parallel single cell multi-omics (e.g. scMT and sciCAR). The software is available on github and has walkthrough examples on how to use the software. The novel feature of the method is that it performs aggregation to increase signal of epigenetic data and all required quantities are inferred in a joint model. The authors also introduce a novel approach to visualise genes, loci and cells in the same 2D space, which might be really useful for interpreting multi-omics data.

- Is the paper of broad interest to others in the field, or of outstanding interest to a broad audience of biologists?

Yes, as the field progresses in profiling multiple molecular layers from the same single cell, integrative methods such as scAI will be relevant.

*Response: We appreciate the reviewer’s positive comments.*

**Second round of review**

**Reviewer 1**

This paper does focus on a crucial aspect of the transcriptomic and epigenomic integration analysis. It provides a matrix factorization model which delivers consistent improvements for various datasets. My concerns has been properly addressed in the revision. I don't have further comments.

**Reviewer 2**

\# scAI: an unsupervised approach for the integrative analysis of parallel single-cell transcriptomic and epigenomic profiles

In general the authors replied to all the concerns raised in the original submission. They also provided additional examples and detailed explanation of their method and results.

A minor concern is the usage of their method for single cell methylation data. I am not convinced the strategy for considering methylated CpGs and missing values with the same encoding is appropriate. The methylation data (e.g. scBS-seq) give you more information since they can clearly distinguish between the three states: methylated, unmethylated and missing. And to apply the scAI method one would need to throw some of this information away; that is why I believe one should be cautious when using it for scM&T-seq and related methods.

**Authors Response**

**Point-by-point responses to the reviewers’ comments:**

Reviewer #1

This paper does focus on a crucial aspect of the transcriptomic and epigenomic integration analysis. It provides a matrix factorization model which delivers consistent improvements for various datasets. My concerns has been properly addressed in the revision. I don't have further comments.

*Response: We thank the reviewer for the insightful comments and suggestions to help us improve the manuscript.*

Reviewer #2

1. In general the authors replied to all the concerns raised in the original submission. They also provided additional examples and detailed explanation of their method and results.

*Response: We thank the reviewer for the insightful comments and suggestions to help us improve the manuscript.*

2. A minor concern is the usage of their method for single cell methylation data. I am not convinced the strategy for considering methylated CpGs and missing values with the same encoding is appropriate. The methylation data (e.g. scBS-seq) give you more information since they can clearly distinguish between the three states: methylated, unmethylated and missing. And to apply the scAI method one would need to throw some of this information away; that is why I believe one should be cautious when using it for scM&T-seq and related methods.

*Response: Thanks for raising this point. We now have added the following paragraph in Discussion on page 20.*

*The single-cell multiomics data are sparse and have large amounts of missing values. The scRNA-seq data have two states: non-zero and zero values. The zero values might be either non-expressed values or due to dropout events [59]. The single-cell methylation data have three states: methylated, unmethylated and missing values. While replacing missing values by zeros and adopting a model that can potentially impute the missing values, a strategy used in scAI, might improve downstream analysis due to the fact that the large portions of missing values contain true zero values, such approach likely has several limitations. First, it might introduce false signals when the missing values might actually correspond to non-zero signals. Second, such approach cannot distinguish methylated and missing states for the DNA methylation data. One way to address such difficulty is to throw away the missing values, which is particularly useful for the methylation data (e.g. scM&T-seq [3]) because it allows to distinguish methylated and missing values. One powerful approach is to use probabilistic models, such as MOFA [17] and its successor MOFA+ [19], which do not include those missing value regions when computing the likelihood. In principle, we can throw away the missing values in scAI by incorporating a binary matrix into the second term of our model (Eq.(1)), an approach similar to incomplete nonnegative matrix factorization model [60]).*
